# Insights into the Origin of Nematode Chemosensory GPCRs: Putative Orthologs of the Srw Family Are Found across Several Phyla of Protostomes

**DOI:** 10.1371/journal.pone.0093048

**Published:** 2014-03-24

**Authors:** Arunkumar Krishnan, Markus Sällman Almén, Robert Fredriksson, Helgi B Schiöth

**Affiliations:** Department of Neuroscience, Functional Pharmacology, Uppsala University, Uppsala, Sweden; University of Geneva, Switzerland

## Abstract

Nematode chemosensory GPCRs in *Caenorhabditis elegans* (NemChRs) are classified into 19 gene families, and are initially thought to have split from the ancestral *Rhodopsin* family of GPCRs. However, earlier studies have shown that among all 19 NemChR gene families, only the srw family has a clear sequence relationship to the ancestral *Rhodopsin* GPCR family. Yet, the phylogenetic relationships between the srw family of NemChRs and the *Rhodopsin* subfamilies are not fully understood. Also, a widespread search was not previously performed to check for the presence of putative srw family-like sequences or the other 18 NemChR families in several new protostome species outside the nematode lineage. In this study, we have investigated for the presence of 19 NemChR families across 26 eukaryotic species, covering basal eukaryotic branches and provide the first evidence that the srw family of NemChRs is indeed present across several phyla of protostomes. We could identify 29 putative orthologs of the srw family in insects (15 genes), molluscs (11 genes) and *Schistosoma mansoni* (3 genes). Furthermore, using HMM-HMM profile based comparisons and phylogenetic analysis we show that among all *Rhodopsin* subfamilies, the peptide and SOG (somatostatin/opioid/galanin) subfamilies are phylogenetically the closest relatives to the srw family of NemChRs. Taken together, we demonstrate that the srw family split from the large *Rhodopsin* family, possibly from the peptide and/or SOG subfamilies, well before the split of the nematode lineage, somewhere close to the divergence of the common ancestor of protostomes. Our analysis also suggests that the srsx family of NemChRs shares a clear sequence homology with the *Rhodopsin* subfamilies, as well as with few of the vertebrate olfactory receptors. Overall, this study provides further insights into the evolutionary events that shaped the GPCR chemosensory system in protostome species.

## Introduction

All animals recognize and respond to chemosensory information in their environment. In most multicellular animals, the ability to sense the environment relies largely on the membrane bound chemosensory receptors, which detect environmental chemical stimuli and convert it into intracellular responses [1], [2]. Also, in most eukaryotes, the chemosensory receptors belong to the superfamily of G protein-coupled receptors (GPCRs), which are crucial for many physiological processes and constitute the dominant signaling system in metazoans [2], [3], [4], [5]. Chemosensory GPCR families include vertebrate olfactory receptors (ORs) [6], trace amine-associated receptors (TAAR) [7], vomeronasal receptors type 1 and 2 (VR 1 & 2) [8], [9], [10], taste receptors type 1 and 2 (TR 1 & 2) [11], [12], and a large group of nematode chemosensory receptors (NemChRs) [13].

Chemosensory GPCRs in the nematode worm *Caenorhabditis elegans* (NemChRs) are classified into 19 gene families based on sequence similarities and monophyletic clustering of genes [14]. Similarly, 15 of these 19 gene families were grouped into three major superfamilies named *Sra*, *Str* and *Srg*, which contain four (sra, srab, srb and sre), five (srd, srh, sri, srj and str), and six (srg, srt, sru, srv, srx and srxa) families, respectively [14]. The other four families, srbc, srsx, srw and srz were instead classified as “others” based on sharing low sequence similarity with all other NemChR families [14]. Similar to NemChR families, ORs have undergone expansions in many mammalian species and are by far the largest mammalian gene family [2], [15]. Although, ORs appear to have undergone large expansions, only a small fraction of them were found in the deuterostome invertebrates [16], [17], [18]. On the other hand, the pheromone receptors VR type 1 and 2 and TR type 1 (sweet) and 2 (bitter) are found to be specific to vertebrates [19], [20], [21]. Considering these observations, it is apparent that the chemosensory GPCRs appears to have evolved multiple times independently, as chemosensory GPCRs in deuterostomes (vertebrate OR, TAAR, VR 1 & 2 and TR 1 & 2) are not closely related to those found in protostomes (NemChRs).

Interestingly, all chemosensory GPCR gene families are suggested to have a common origin despite their low sequence similarity and the diverse nature of their signaling molecules. Moreover, earlier studies have suggested that the chemosensory GPCRs might have evolved from the ancient *Rhodopsin* family of GPCRs [22]. Likewise, whole genome studies as well as previous GPCR mining studies in the basal eumetazoan lineages suggested that the diversifications of the large *Rhodopsin* family into subfamilies, like amines and peptides, as well as the olfactory receptors have occurred before the protostome and deuterostome split [22], [23], [24], [25]. To support this further, a recent study also showed that the cnidarian *Nematostella vectensis* has 35 full length chordate like OR genes [26]. This suggests that the common ancestor of the cnidarians and bilateral animals had chordate like OR genes that expanded greatly in deuterostomes. However, these chordate like OR genes were subsequently lost in all protostomes that evolved a differential chemosensory system, which includes NemChRs.

Although earlier studies support the fact that the chemosensory GPCRs split from the large *Rhodopsin* family, little is known about the relationships between the NemChR families and the *Rhodopsin* like GPCRs. Intriguingly, among all 19 NemChR gene families, only the srw family have been identified to have a clear sequence relationship with the subfamilies of the *Rhodopsin* (7tm_1) superfamily [14], [22]. Yet, the phylogenetic relationships between the srw family of NemChRs and the *Rhodopsin* subfamilies are not fully understood. Furthermore, the presence of putative homologs of these 19 NemChR families in species other than nematodes is not thoroughly examined. In the current study, we have investigated the presence of NemChRs in 26 genomes that comprise all eukaryotic supergroups. We demonstrate that the srw family of NemChRs is found across several protostome phyla and it split from the large *Rhodopsin* family, possibly from the peptide and the SOG subfamilies, well before the split of the nematode lineage, somewhere close to the divergence of the common ancestor of protostomes.

## Materials and Methods

### Proteome dataset

Proteomes were downloaded from Ensembl Metazoa (http://metazoa.ensembl.org) for Anopheles gambiae, Acyrthosiphon pisum, Apis mellifera, Pediculus humanus, Daphnia pulex, Pristionchus pacificus and Schistosoma mansoni; Oryza sativa and Arabidopsis thaliana proteomes were downloaded from Ensembl Plants (http://plants.ensembl.org); Trypanosoma brucei and Tetrahymena thermophila proteomes were downloaded from Ensembl Protists (http://protists.ensembl.org); Homo sapiens, Mus musculus, Gallus gallus, Xenopus tropicalis, Danio rerio and Petromyzon marinus proteomes were downloaded from Ensembl (http://www.ensembl.org); the C. elegans proteome was downloaded from WormBase (http://www.wormbase.org); N. vectensis, Trichoplax adhaerens, Phytophtera sojae, Thalassiosira pseudonana, Lottia gigantea and Monosiga brevicollis proteomes were downloaded from the Joint Genome Institute (http://genome.jgi.doe.gov/); Drosophila melanogaster and Drosophila willistoni proteomes were downloaded from FlyBase (http://flybase.org); Dictyostelium discoideum and Dictyostelium fasciculatum proteomes were downloaded from dictyBase (www.dictybase.org); the Entamoeba histolytica proteome was downloaded from amoebaDB (http://amoebadb.org); the Paramecium tetraurelia proteome was downloaded from NCBI (http://www.ncbi.nlm.nih.gov/); the Trichomonas vaginalis proteome was downloaded from TrichDB (http://trichdb.org), and the Giardia lamblia proteome was downloaded from GiardiaDB (http://giardiadb.org). Furthermore, the fungal proteome dataset was downloaded from UniProt database (http://www.uniprot.org/).

### Identification of nematode chemosensory GPCRs (NemChRs)

The proteomes analyzed in this study were searched against a local installation of the Pfam database version 26, which has 13672 families, with sensitive HMM models built using HMMER3 software [27]. We utilized the Pfam_scan.pl script, obtained from the Pfam ftp-site, to align each of the13672 HMM profiles with our proteome dataset. The script Pfam_scan.pl uses homology criterion set by the Pfam database, which is based on a manually curated gathering threshold for each model. The gathering threshold of a Pfam model makes sure that any sequence must attain a score greater than or equal to the threshold to be deemed significant and included in a Pfam-A full alignment. This ensures that there are no false positive sequences in the Pfam-A alignments and thereby increases the accuracy of the Pfam-A models. About 79.9% of the proteins contained in the SWISS-PROT and TrEMBL databases have at least one match to a Pfam-A family. Utilizing these accurate HMM models, we retrieved all the sequences that were assigned to the following Pfam domains (HMM profiles) in the Pfam-search. We searched for the following 19 NemChR family domains, 7TM_GPCR_Sra (PF02117), 7TM_GPCR_Srab (PF10292), 7TM_GPCR_Srb (PF02175), sre (PF03125), srg (PF02118), 7TM_GPCR_Srt (PF10321), 7TM_GPCR_Sru (PF10322), 7TM_GPCR_Srv (PF10323), 7TM_GPCR_Srx (PF10328), srxa (Serpentine_r_xa; PF03383), 7TM_GPCR_Srd (PF10317), 7TM_GPCR_Srh (PF10318), 7TM_GPCR_Sri (PF10327), 7TM_GPCR_Srj (PF10319), 7TM_GPCR_Str (PF10326), 7TM_GPCR_Srbc (PF10316), 7TM_GPCR_Srsx (PF10320), 7TM_GPCR_Srw (PF10324) and 7TM_GPCR_Srz (PF10325). Also, we included 7tm_4 (PF13853), a new Pfam domain exclusive to the vertebrate like olfactory receptors included in the latest Pfam release 26. The separation of the olfactory receptors from the conventional 7tm_1 (*Rhodopsin*) domain facilitates a direct identification of novel members of the olfactory receptor family.

### Identification and categorization of the *Rhodopsin* (7tm_1) family receptors

Using the same procedure mentioned above, we searched in the proteomes of *N. vectensis*, *T. adhaerens*, *D. melanogaster* and *C. elegans* for the sequences containing 7tm_1 (PF00001) as their HMM profile with highest scoring alignment. In order to assign subfamily level classification for the identified *Rhodopsin* family receptors, we performed a standalone BLASTP search against the human GPCRs. We utilized standard default settings for the BLASTP searches, with a word size of 3 and BLOSUM62 scoring matrices. We downloaded the *Rhodopsin* family GPCRs from our human GPCR repertoire [28] and tagged them with their subfamily categorization. Afterwards, the *Rhodopsin* family receptors from all four species (*N. vectensis*, *T. adhaerens*, *D. melanogaster* and *C. elegans*) were searched against a database consisting of these tagged human *Rhodopsin* family of GPCRs. To categorize the sequences into subfamilies, the classification criteria were that they must have at least four of the five best hits from the same subfamily in the BLASTP search. Thereafter, each family in four species was separately aligned using the MAFFT program, with option E-INS-I and BLOSUM62 as the scoring matrix. Each alignment was thereafter examined and refined in Jalview 2.5.1, i.e. the sequences were trimmed and well conserved and aligned regions were kept. From the alignments, Hidden Markov Models (HMMs) were constructed using the HMMER3 package. The models were constructed using the HMMbuild program with default settings.

### HMM-HMM profile comparisons

To compare two HMM profiles, we utilized the HHsearch program with default options as implemented in the HH-suite software package. HHsearch is considered to be one of the most sensitive methods for protein homology detection [29]. It is widely used by the Pfam database to determine the relationships between families and to assign a Pfam clan where the homologous families are grouped together [27], [30]. The HHsearch program describes a probability score for homologous relationship, which is considered as an appropriate measure to decide whether a hit is a true homolog to the query and can be considered a more intuitive measurement than the commonly used E-value for evaluating the significance of the search result. According to the HHsearch/HH-suite user guide (ftp://toolkit.genzentrum.lmu.de/pub/HH-suite/hhsuite-userguide.pdf) a probability of >95% is considered a homology that is nearly certain [29].

### Consensus sequences

Consensus sequences of each gene family used in this study were generated from their corresponding HMM profiles. Each HMM profile that corresponds to a particular family serves as an input for the HMMEMIT program and thus a consensus sequence was obtained using option ‘-C’ as implemented in the HMMER3 package. The consensus sequence is formed using a plurality rule that selects the maximum probability residue at each match state from the HMM profiles.

### Multiple sequence alignment and phylogenetic tree construction

Multiple sequence alignments analyzed in this study were generated using MAFFT version 6 (http://mafft.cbrc.jp/alignment/server/), with BLOSUM62 as the scoring matrix and using option E-INS-I (recommended for sequences with conserved motifs and carrying multiple domains) [31], [32]. Thereafter alignments were manually inspected and trimmed to 7TM regions using Jalview software [33]. The phylogenetic analysis was performed using the Bayesian approach implemented in MrBayes version 3.2 [34]. Markov Chain Monte Carlo (MCMC) analysis was used to estimate the posterior probabilities and branch lengths of the trees. To determine the best amino acid substitution model, a mixed option (aamodelpr = mixed) was used. Gamma shaped model was used to estimate the variation of evolutionary rates across sites (lset rates = gamma). All Bayesian analyses conducted in this study included two independent MCMC runs, where each MCMC run uses 4 parallel chains composed of three heated and one cold chain. Each Markov chain was started from a random tree and was set to run for 3,000,000 generations and every hundredth tree was sampled. To test the convergence of the two entirely independent runs starting from different random trees, diagnostic frequency (diagnfreq) generations were performed and diagnostics were calculated for every 1000 generations (diagnfreq  = 1000). To determine when to terminate the MCMC generations, a stop rule was applied (standard deviation of split frequencies <0.01). In order to ensure that the parameter estimates were only made from data drawn from distributions derived after the MCMCs had converged, we discard the first 25% of the sampled trees using the “relburnin” setting (relburnin  =  yes and burninfrac  = 0.25). After discarding the “burn-in” samples, MCMC runs were summarized and further investigated for convergence of all parameters, using *sump* and *sumt* commands in MrBayes software. Thereafter a consensus tree was built from the remaining 75% of the sampled trees with the MrBayes *sumt* command using the 50% majority rule method. The *sump* command was used to assure that an adequate sample of the posterior probability distribution was reached during the MCMC procedure. The phylogenetic tree was drawn in FigTree 1.3.1 (http://tree.bio.ed.ac.uk/software/figtree/). The topology of all the Bayesian phylogenetic trees supported by the posterior probability (PP) was cross verified with bootstrap analysis (500 replicates) using the maximum likelihood (ML) approach implemented in the PhyML (version 3.0) program [35]. Bootstrap values were indicated as percentage for the nodes that received good support in ML approach.

## Results

### Search for novel members of the NemChR families across several eukaryotes

**Figure 1:**
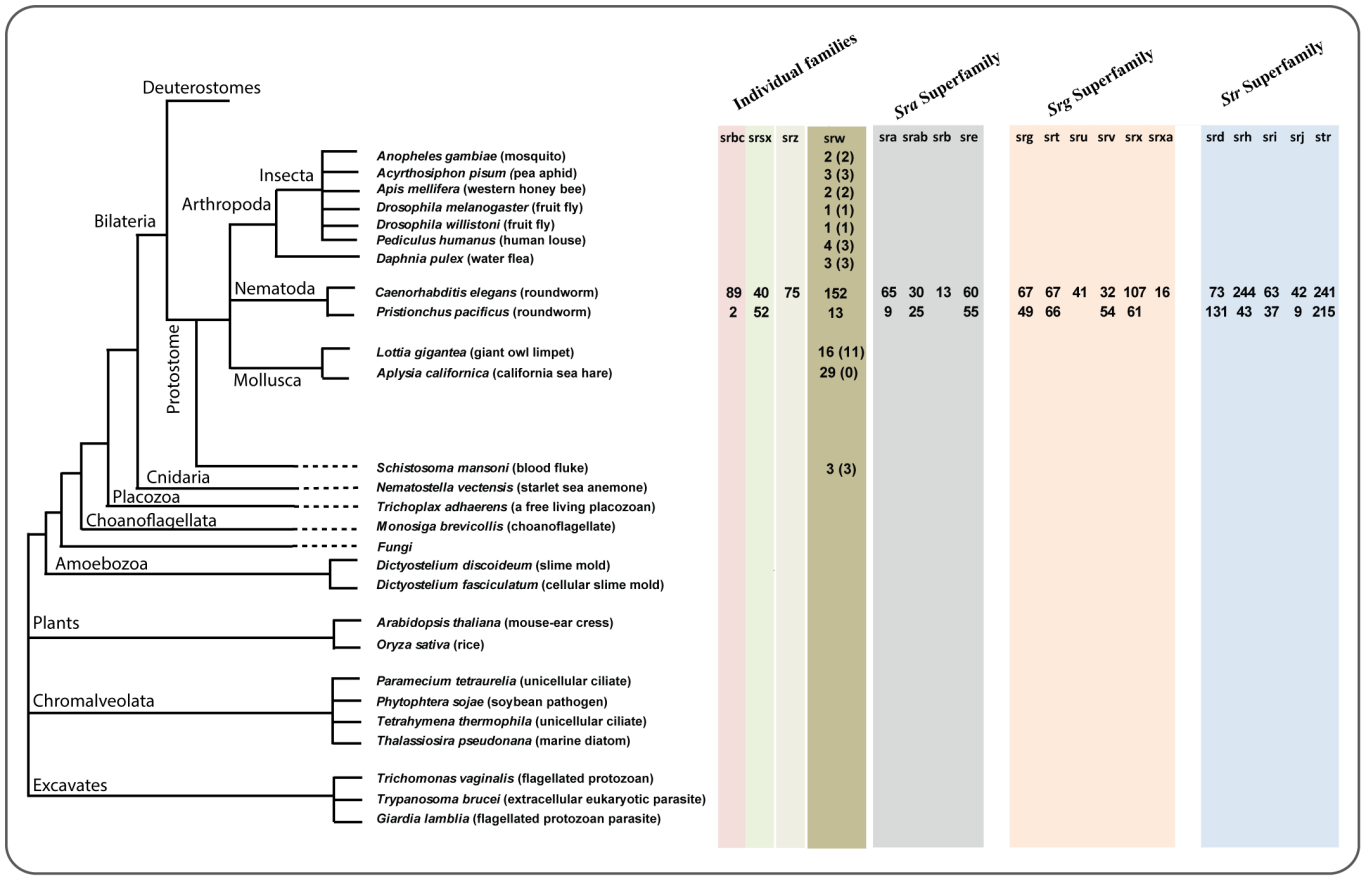
Schematic diagram shows the nematode chemosensory gene families (NemChRs) across the analyzed taxa. Numbers within the parenthesis in the srw column represent the actual number of the srw like sequences that clustered with the annotated srw family members from nematodes (*C. elegans* and *P. pacificus*).

We performed Pfam HMM profile searches and manual inspection of conserved amino acid motifs to check for the presence of NemChR families sequences across 26 species from several different phyla of eukaryotes. Interestingly, among 19 NemChR families, we identified putative srw (PF10324) family-like sequences across several phyla of protostomes. However, we failed to identify full length srw family-like sequences in eukaryotes other than the species analyzed from the superphylum of protostomes. Overall, we identified 35 novel genes encoding the 7tm_GPCR_srw (PF10324) domain in the analyzed genomes of insects (16 genes from 7 species), molluscs (16 genes from *L. gigantea*) and *S. mansoni* (3 genes) (see Figure 1, Table S1 and Dataset S1). Furthermore, we examined a total of 90 chemosensory like genes identified in the mollusc *Aplysia californica* [36] and found that 29 of these sequences had 7tm_GPCR_srw as their Pfam domain with highest scoring alignment (Figure 1, Table S1 and Dataset S1). In addition, our Pfam search identified fragments in the cnidarian *N. vectensis* (Nv_ 210893) and amoebozoa *D. fasciculatum* (Df_F4PUK6) that had the highest alignment score against the 7tm_GPCR_srw domain. Also, our analysis found sequence fragments in *N. vectensis* (Nv_205247), as well as in *T. adhaerens* (Ta_58780), which had 7TM_GPCR_Srsx (PF10320) domain as their significant Pfam match. All putative NemChR family members identified using initial Pfam search was subsequently examined whether they are true positives hits using phylogenetic analysis (described in the next sections).

### Phylogenetic verification of srw family-like sequences identified in the Pfam search

**Figure 2:**
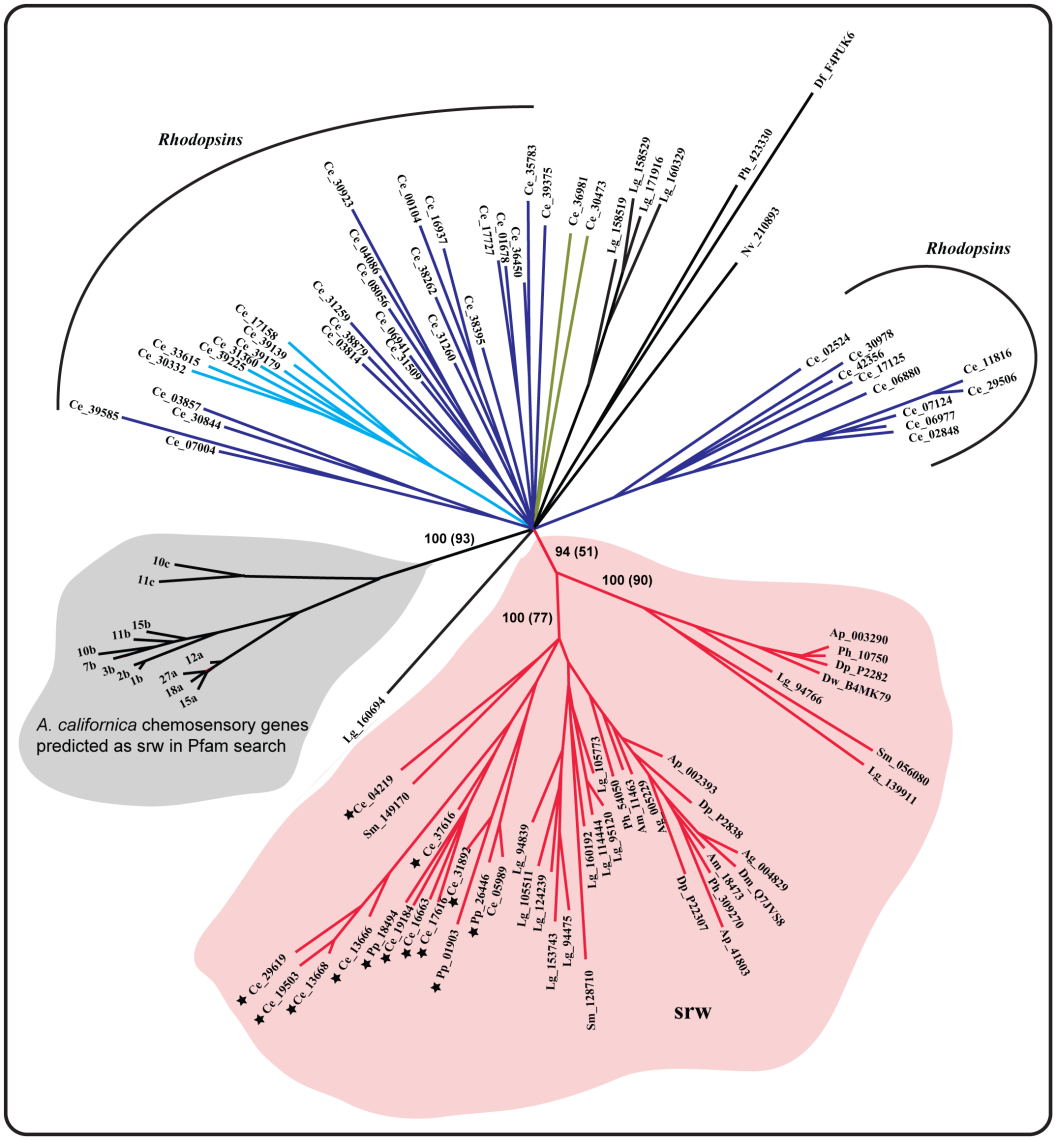
Phylogenetic relationships of the *Rhodopsin* subfamilies, the srw family from *C. elegans* and the srw like sequences identified from several protostome species. The tree topology was inferred from Bayesian analysis with a gamma correction using MrBayes software. The *Rhodopsin* subfamilies from *C. elegans* included the peptide (violet), amine (light blue) and the SOG (olive green) subfamilies, among others. The major clade that clustered the novel srw like sequences with the known srw family members from nematodes (*C. elegans* and *P. pacificus*) is highlighted in red (the conserved 7TM regions of the sequences from srw clade are shown in Figure S2). Previously known srw family members from nematodes (*C. elegans* and *P. pacificus*) are marked with a star symbol. Sequences that had the highest alignment score against the 7tm_GPCR_srw domain, but failed to cluster within the srw clade are marked with black edges. Similarly, the chemosensory genes in *A. californica* that had the highest alignment score against the 7tm_GPCR_srw domain in our Pfam search, but clustered separately from the srw family members from nematode is highlighted in grey. We followed the same renaming for the *A. californica* genes as previously used [36] for easy cross verification. Abbreviations used in the figure includes, Ap (*Acyrthosiphon pisum*), Ag (*Anopheles gambiae*), Am (*Apis mellifera*), Ce (*Caenorhabditis elegans*), Df (*Dictyostelium fasciculatum*), Dm (*Drosophila melanogaster*), Dp (*Daphnia pulex*), Dw (*Drosophila willistoni*), Lg (*Lottia gigantea*), Ph (*Pediculus humanus*), Pp (*Pristionchus pacificus*), Sm (*Schistosoma mansoni*) and Nv (*Nematostella vectensis*). Posterior probabilities and bootstrap replicates (within parenthesis) are shown as a percentage for the major nodes.

**Figure 3:**
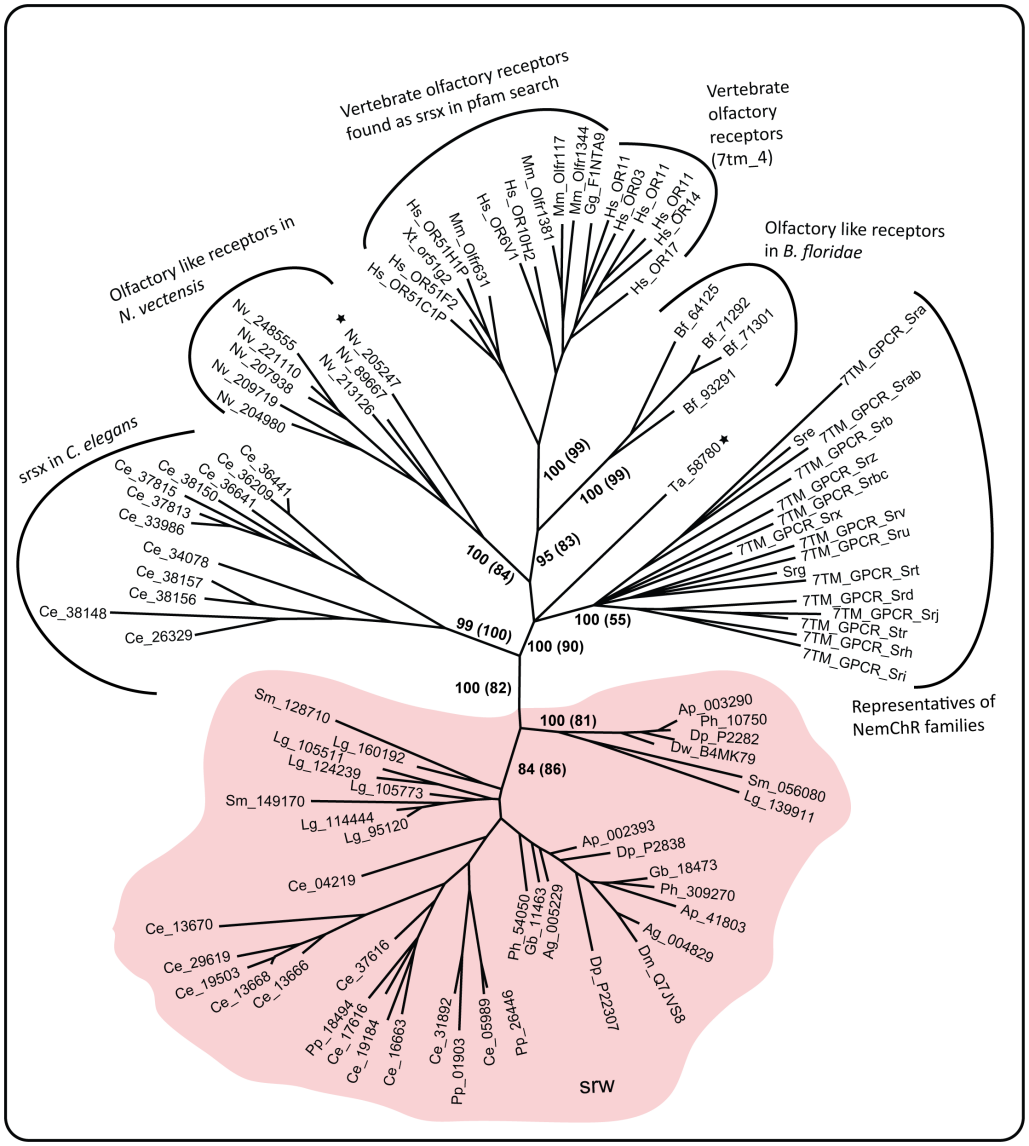
Phylogenetic relationships between the srsx, srw and the olfactory like receptors. The phylogenetic tree includes 1) representatives of srsx family in *C. elegans*, 2) previously known srw family members from nematodes (*C. elegans* and *P. pacificus*), 3) srw like sequences identified in this study, 4) vertebrate olfactory genes (7tm_4), 5) olfactory like receptors in *B. floridae*, 6) olfactory like receptors in *N. vectensis* [26], 7) vertebrate olfactory sequences that had significant alignment score against the 7tm_GPCR_srsx domain (see Table S2), 8) consensus representative for each NemChR families, and 9) the sequence fragments from *N. vectensis* (Nv_205247) and *T. adhaerens* (Ta_58780) that had the highest scoring alignment against the 7tm_GPCR_Srsx domain (indicated with a star symbol). Posterior probabilities and bootstrap replicates (within parenthesis) are shown as a percentage for the major nodes.

**Figure 4:**
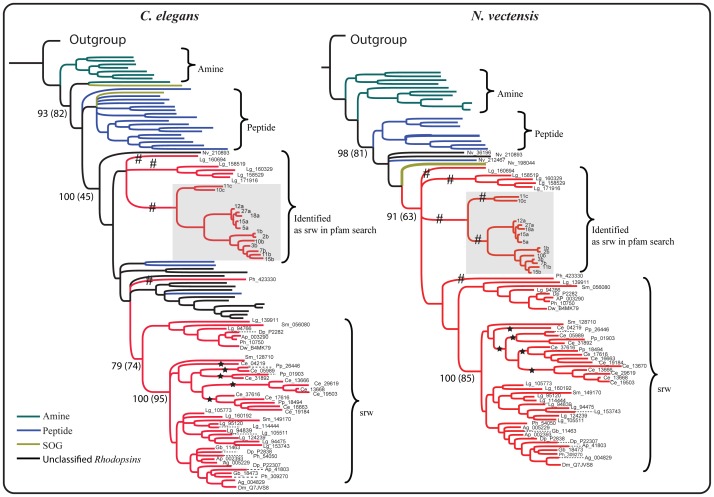
Phylogenetic trees showing closest related *Rhodopsin* subfamilies to the srw family. Olfactory like genes identified in *N. vectensis* is used as outgroup and subsequently rooted. The *Rhodopsin* subfamily sequences included in the trees were obtained using srw family sequences as queries in BLASTP searches against the *Rhodopsin* family repertoire of *C. elegans* and *N. vectensis*. The top hits included sequences from peptide (violet), SOG (olive green), amine (dark green) subfamilies and some unclassified *Rhodopsin* family sequences (black) that did not have at least four of the five best hits from the same subfamily in a BLASTP search against human GPCR repertoire (see methods). The topology of trees shows that the peptide (violet) and SOG (olive green) subfamilies are placed basal to the srw clade. Posterior probabilities and the number of bootstrap replicates (within parenthesis) are shown as a percentage for the major nodes. The branches that contain srw family members from nematodes (*C. elegans* and *P. pacificus*) are indicated with a star symbol. The branches that contain sequences that are predicted to be srw in the Pfam search and yet separated from the node clustering srw family members from nematodes are indicated with hash (#) symbol. *A. californica* chemosensory genes that had the highest alignment score against the 7tm_GPCR_srw domain in our Pfam search is highlighted in grey box. See supplementary Figure S1 to view the phylogenetic relationships of the *Rhodopsin* subfamilies and the srw family in all four analyzed species (*C. elegans*, *N. vectensis*, *D. melanogaster* and *T. adhaerens*).

**Figure 5:**
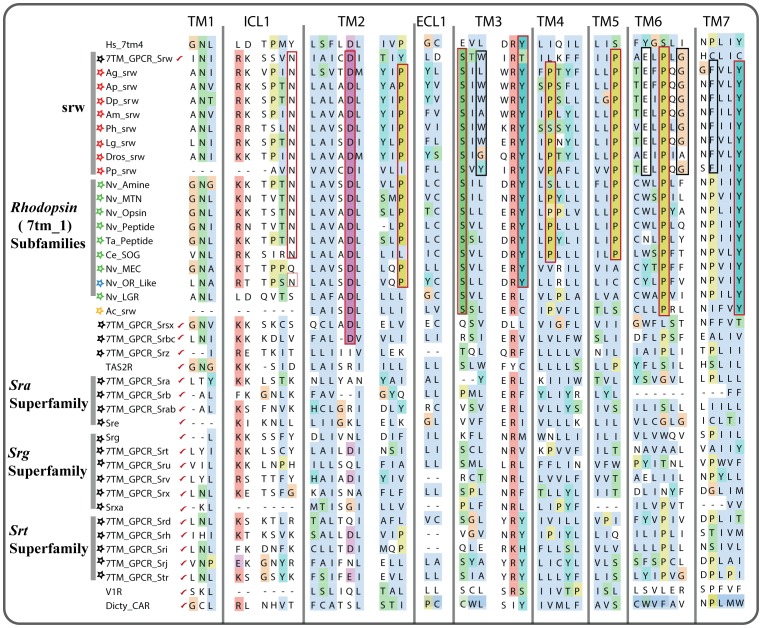
Multiple alignments of chemosensory GPCR families and *Rhodopsin* like GPCR subfamilies. Every sequence in the alignment is a consensus sequence obtained from the HMM models of the respective family using the HMMEMIT program (see materials and methods). We include consensus sequence for 1) vertebrate olfactory receptor family members from human (Hs_7tm_4), 2) srw like sequences identified in the genomes of insects, mollusk and *S. mansoni* (red stars), 3) *Rhodopsin* subfamilies from *C. elegans*, *N. vectensis* and *T. adhaerens* (green stars), 4) Olfactory like genes identified in *N. vectensis* (blue star), 5) srw like sequences identified in *A. californica* (yellow star), 6) 19 nematode chemosensory GPCR families from *C. elegans* (black stars), 7) mammalian taste receptor family (TAS2R), 8) vomeronasal receptors (V1R) and 9) the ancient *Dictyostelium* Cyclic AMP receptors (Dicty_CAR). The consensus sequences that are marked with a red tick symbol are obtained from Pfam HMM models downloaded from the Pfam database, while other sequences are obtained from the HMM models constructed using the multiple alignments of our sequence datasets. The red rectangular boxes indicate residues that are conserved across the srws and the *Rhodopsin* subgroups, whereas the black rectangular boxes indicate residues that are quite specific to the srw family.

In order to verify the list of putative srw family like sequences (35) identified in the Pfam search (mentioned above), we combined those 35 sequences on the list with a dataset consisting of known and annotated srw family sequences from the nematodes, *C. elegans* and *P. pacificus* (obtained from database searches and earlier studies[14]). Phylogenetic analysis on the dataset demonstrated that 29 of 35 novel srw like sequences identified in the genomes of insects, *L. gigantea* and *S. mansoni* clustered with the annotated srw family sequences (94% posterior probability (PP), 51% of ML bootstrap support, see Figure 2) from the nematodes *C. elegans* and *P. pacificus* (also see Figure 3). Furthermore, these sequences were clearly separated from the *Rhodopsin* family members (see Figure 2). This topology is consistent in our analysis to identify the most closely related *Rhodopsin* subfamily to the srw family (see Figure 4 and Figure S1). Furthermore, the list of novel srw members has several residues conserved with the srw sequences from *C. elegans*, when included in the multiple sequence alignments (Figure 5 and Figure S2). In contrast, the chemosensory receptor like genes in the mollusc *A. californica* (which had 7tm_GPCR_srw domain as their as their highest scoring alignment), fall into a cluster separate from the srw family representatives from *C. elegans* (Figure 2). In addition, the putative fragments in *N. vectensis* and *D. fasciculatum* (which contains the 7tm_GPCR_srw domain as their best hit) clustered separately from the srw cluster. This suggests that they are divergent or false positives from the Pfam search (Figure 2). Taken together, we consider only those 29 sequences from protostomes that clustered with the known srw family sequences in *C. elegans* and *P. pacificus* to be putative orthologs of the srw family (Figure 2). However, we describe these 29 sequences from protostomes as putative orthologs merely on the basis of the phylogenetic clustering and not based on functions. Because the chemosensory GPCRs in *C. elegans* are classified largely based on the sequence analysis and indeed none of the srw genes were experimentally verified to function as chemosensory receptors [13], [14], [37].

### Phylogenetic clarification of putative srsx family like sequences identified in the Pfam search

Besides identifying novel srw family members from protostomes, our analysis also found sequence fragments in *N. vectensis* (Nv_205247), as well as in *T. adhaerens* (Ta_58780), which had 7TM_GPCR_Srsx (PF10320) domain as their significant Pfam match with E-values (E) 8.3e−05 and 1.5e−08, respectively. However, Pfam search showed that the fragment identified in *T. adhaerens* (Ta_58780) can also be readily aligned with the 7tm_1 (PF00001) domain (residues 85 to 281, E = 2.9e−23), as well as the srsx domain (residues 40 to 99, E = 1.5e−08) (see Table S2). Similarly, our Pfam search also identified a few vertebrate olfactory receptors that had two significant Pfam HMM profile hits corresponding to srsx domain (PF10320) and 7tm_4 domain (PF13853), within their transmembrane spanning regions. Such sequences, which had Pfam family overlaps within the same sequence regions were found in human (13 genes), mouse (43 genes), chicken (1 gene) and frog (1 gene) (Table S2). Although these sequences had two significant Pfam HMM profile hits within their transmembrane regions, in each and every instance the 7tm_4 domain had better E-values when compared to the srsx domain (Table S2). However, according to the Pfam documentation [27], if the same region of a sequence matches two Pfam families, it should be considered a false positive in one of them, but if the domain hits are from the same Pfam clan then the overlap is believed to reflect an evolutionary relationship between two Pfam families.

In order to clarify these Pfam predictions and the relationships between the srsx family and vertebrate olfactory receptors (7tm_4), we performed phylogenetic analysis on a comprehensive dataset. The dataset included 1) srsx family sequences from *C. elegans*; 2) vertebrate olfactory sequences that had srsx domain (PF10320) as a significant hit; 3) functional olfactory family sequences from humans that encodes the 7tm_4 (olfactory) domain; 4) vertebrate like olfactory genes identified in *N. vectensis* [26]; 5) srsx like fragments identified in *T. adhaerens* (Ta_58780) and *N. vectensis* (Nv_205247); 6) all novel srw like sequences and 7) consensus sequences for other NEMCHR families. The overall unrooted tree shows a topology where the srw and srsx family sequences from *C. elegans* fall into two distinct and separate clusters (Figure 3). Furthermore, the vertebrate olfactory receptors, which unusually showed high similarity to the srsx domain in the Pfam search, clustered with the olfactory receptors (100% PP, 99%) that had the 7tm_4 (olfactory domain) as their highest scoring alignment. In addition, the fragment Nv_205247clustered (100% PP, 84%) with the vertebrate like olfactory genes identified in *N. vectensis*, indicating that it is likely a member of the olfactory receptor family, and not srsx (Figure 3). Similarly, the fragment Ta_58780 in *T. adhaerens*, which had two significant Pfam HMM profile hits corresponding to 7tm_1 and srsx domains, clustered separately from the other srsx proteins (Figure 3). This suggests that although Ta_58780 bears resemblance to srsx, it is more likely a member of the 7tm_1 family (Figure 3).

### Identification of the closest related *Rhodopsin* subfamily to the srw family

In order to find the closest related *Rhodopsin* subfamily to the srw family, we compared each *Rhodopsin* subfamily from four different species with the srw family. We included two species that diverged before the protostome-deuterostome split, *T. adhaerens* and *N. vectensis*, which contain large and diverse repertoires of *Rhodopsin* family sequences. Also, *T. adhaerens* and *N. vectensis* represent the most distant lineages where the vertebrate like *Rhodopsin* subfamilies have been identified [23], [24], [25]. Additionally, we included *D. melanogaster* and *C. elegans* from the protostome superphylum. Our analysis strategy included two independent ways to identify the most closely related *Rhodopsin* subfamily to the srw family. First, the HMM-HMM profile based comparisons were performed using the HHsearch program. We compared the HMM profiles of the srw family from different species against a database that contained HMM profiles of the *Rhodopsin* subfamilies from *T. adhaerens*, *N. vectensis*, *D. melanogaster* and *C. elegans*. The database also included HMM profiles of families belonging to the GPCR_A Pfam clan. Thereafter, phylogenetic analysis was performed on a dataset that included representatives of all *Rhodopsin* subfamilies (peptides, amines, SOG, etc) and the entire set of srw family members identified in this study. A flowchart describing the strategy was illustrated in Figure S3.

Interestingly, from the HMM-HMM profile based comparisons, we found that the peptide receptor subfamily was the most closely related to the srw family (see Figure S4). HMM profiles of the srw family form 12 different species served as separate queries against our database of concatenated HMM profiles (see above). For at least nine of these 12 srw HMM profiles as queries, the HMM profile of peptide receptor subfamily was the top hit with greater than 95% probability (see methods for the explanation of HHsearch probability score). Moreover, our results showed that, among the other *Rhodopsin* subfamilies, the SOG (somatostatin/opioid/galanin) subfamily was found to be second closest to the srw family (Figure S4). Also, amine subfamily was among the top five hits, but, with relatively low probability score (around 90%), when compared to the peptide and SOG subfamilies.

In order to verify the HMM-HMM profile comparison results (shown above), we performed phylogenetic analysis to compare the *Rhodopsin* subfamilies from four different species (*T. adhaerens*, *N. vectensis*, *C. elegans* and *D. melanogaster*), with the entire set of srw family members. Phylogenetic trees representing each species were constructed and in all of them the vertebrate like OR genes identified in the cnidarians *N. vectensis* was used as outgroup and subsequently rooted. The phylogenetic grouping in all four species indicated that among all *Rhodopsin* subfamily members, the peptide subfamily was the most closely related to the srw family (Figure 4 and Figure S1). We observed that in all four phylogenetic trees, the peptide subfamily sequences were consistently placed basal to the srw clade, despite the inclusion of representative members from different *Rhodopsin* subfamilies (Figure 4 and Figure S1). The confidence measures (PP and bootstrap) were high in the trees corresponding to *C. elegans* (93%, 82%), *N. vectensis* (98%, 81%) and *T. adhaerens* (93%) (Figure 4 and Figure S1). Moreover, the phylogeny also supports the notion that the SOG subfamily was also closely related to the srw family. The SOG subfamily members were placed on the same branch as the peptide subfamily members in the trees representing *C. elegans*, *N. vectensis* and *D. melanogaster*, whereas in *T. adhaerens* they clustered basal to the peptide subfamily (Figure 4). Overall, this was consistent with the HMM-HMM profile based comparisons, which suggested that the peptide receptor family is the most closely related *Rhodopsin* subfamily to the srw family, followed by the SOG family of receptors. (Figure S4).

### Pairwise HMM-HMM comparisons between the 19 NemChR families

In order to detect the relationships between the 19 NemChR families, we utilized the Pfam HMM profiles of all 19 families and performed HMM-HMM comparisons using the HHsearch program. HHM-HMM comparisons demonstrated that all the families belonging to the *Str* superfamily (srd, srh, sri, srj and str families) shared a HHsearch probability score of around 95% between them (Figure S4). However, the families belonging to the *Sra* superfamily (sra, srab, srb, and sre) and *Srg* superfamily (srg, sru, srv, srx, srt, srxa) did not exhibit greater than 95% probability in all pairwise HMM-HMM alignments. For example, in the *Sra* superfamily, alignment between the HMMs of the sra and sre families had a low probability score of 28%. Similarly, in the *Srg* superfamily, pairwise alignments between the HMMs of the srxa and srx families and the HMMs of the srt and srv families shared very low probability scores around 18.4% and 13.2%, respectively. In the case of individual families (srbc, srx, srw and srz) none of them had reliable hits when compared with the other NemChR families. Instead, the srw and srsx families shared greater than 95% probability with the peptide receptor subfamily belonging to the *Rhodopsin* family of GPCRs.

## Discussion

Nematode chemosensory GPCRs (NemChRs) in *C*. *elegans* that are crucial for sensing various environmental cues, are classified into 19 gene families based on sequence similarities and phylogenetic analysis [14]. These 19 gene families are known to be specific to the nematode lineage [14], [37] and are initially thought to have split from the ancestral *Rhodopsin* family of GPCRs [22]. However, their relationship with the large *Rhodopsin* family of GPCRs is still obscure as the NemChR families does not share a clear sequence relationship with the *Rhodopsin* family of GPCRs, except for the srw family [14]. Also, the presence of putative homologs of these 19 NemChR families in species other than nematodes is not thoroughly examined. In this study, we examined 26 eukaryotic genomes and provide the first evidence for the presence of the nematode chemosensory gene family, srw (7tm_GPCR_srw; PF10324) [14], across several phyla of protostomes (Figure 1 and Figure 2). Furthermore, based on phylogenetic analysis and HMM-HMM comparisons we clarify the relationships between the srw family of NemChRs and the *Rhodopsin* subfamilies of GPCRs.

Here, we investigated 26 eukaryotic species, covering four basal eukaryotic branches, and found that the srw family has 29 putative orthologs across the insect, mollusc and *S. mansoni* genomes. These 29 novel srw members unambiguously clustered with the previously known and annotated srw family from nematodes (*C*. *elegans* and *P. pacificus* with 94% PP) (Figure 2). Furthermore, these novel srw members share several common motifs with the previously known and annotated srw sequences from *C. elegans* (see Figure 5 and Figure S2). Based on these findings, we demonstrate that the srw family emerged much earlier than the other 18 nematode chemosensory families, as we could not find other 18 families in species that diverged earlier than the nematodes. However, during the course of protostome evolution, the srw family has also undergone species specific losses within the mollusc lineage, as we could identify srw members in *L. gigantea*, but not in *A. californica*.

**Figure 6:**
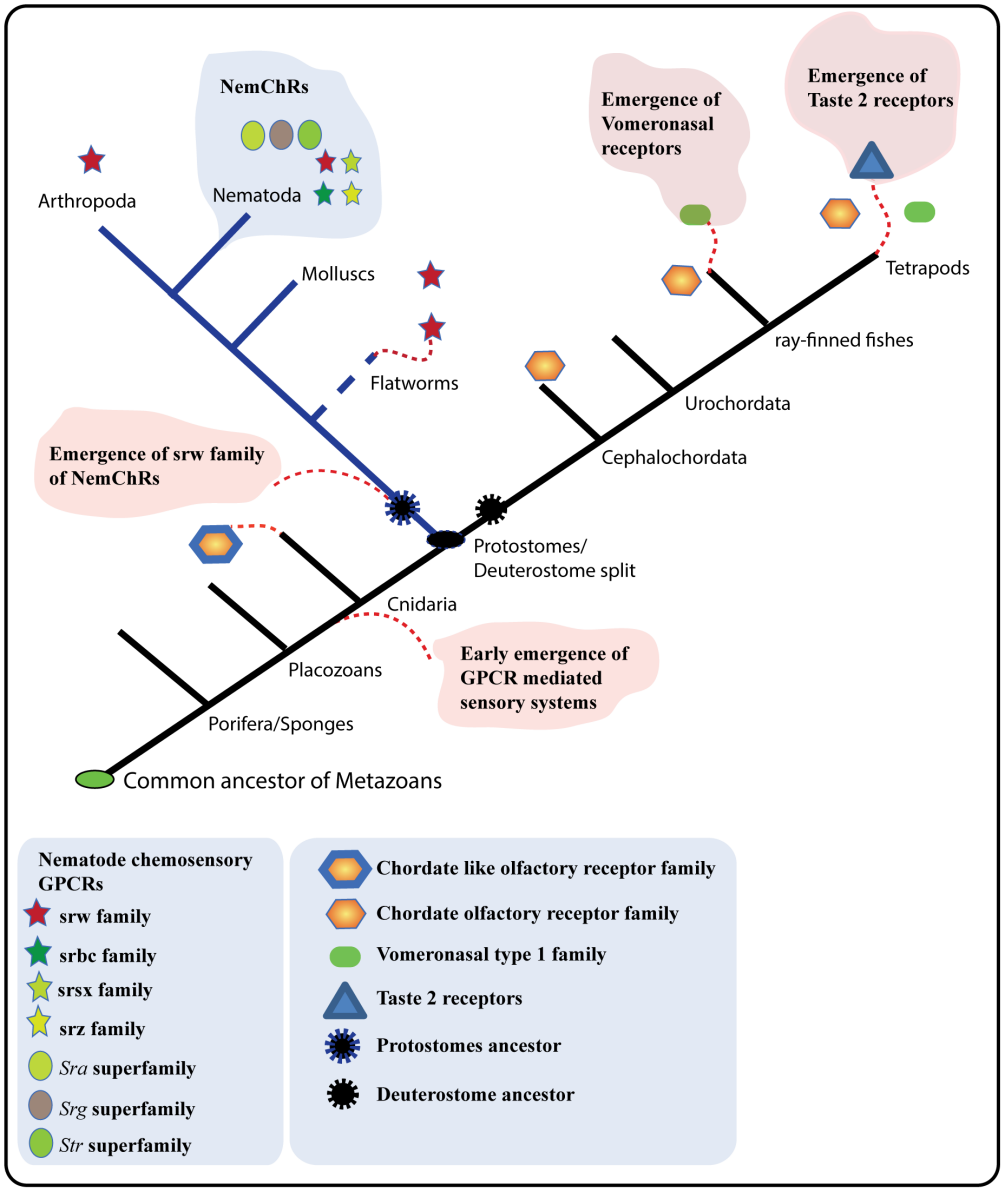
A schematic presentation of the evolution of GPCR mediated chemosensory gene families. The eukaryotic evolutionary tree is constructed with references from the tree of life web project (http://tolweb.org/tree/phylogeny.html). Each gene family is represented with colored symbols and their presence and absence were mapped onto eukaryotic branches. A red arc represents the hypothetical origin of the gene families. The hypothetical origin of these gene families including, chordate like olfactory receptor family, vomeronasal type 1 family and taste 2 receptors were obtained from previous studies [2], [16], [18], [22], [24]. Branch lengths are not drawn to represent actual evolutionary distances.

Through further analysis using HMM-HMM comparisons, we sought to delineate the relationships between the srw family and the other 18 nematode chemosensory families, and as well as with the *Rhodopsin* subfamilies. However, the results show that it is quite difficult to identify a reliable similarity between the srw family and the other 18 NemChRs, even through sensitive HMM-HMM profile based comparisons. This might be due to high divergence within and between these families. Therefore, it is still unclear whether some of the other NemChR families could have split from the srw family. In contrast, we found strong sequence similarity between the srw family and the *Rhodopsin* subfamilies (Figure S4). To further explore this, we performed comprehensive phylogenetic analysis (Figure 4) on the srw family and the *Rhodopsin* subfamilies. We observed that the peptide and the SOG subfamilies of the large *Rhodopsin* family are the closest relatives to the srw gene family. This is also supported by BLASTP searches using the srw sequences as queries against the NCBI nr database, which had peptide receptors among the top hits. Based on the evidence from the phylogenetic analysis and HMM-HMM comparisons, we suggest that the srw family originally split from the large *Rhodopsin* family, possibly from the peptide and/or SOG subfamilies, somewhere close to the divergence of the common ancestor of protostomes (Figure 6).

In addition to the srw family, our HMM-HMM profile comparisons results suggest that the srsx family shares greater than 95% probability (see methods for definition of HHsearch probability score) when compared with the HMMs of *Rhodopsin* subfamilies (Figure S4). The HHsearch results demonstrate that the srsx family shares around 95.7% probability with the peptide receptor subgroup of *C. elegans*. This suggests that the srsx family may have duplicated from the *Rhodopsin* subfamilies, within the nematode lineage. Interestingly, our results also show that the srsx family shares significant similarity to some vertebrate olfactory receptors, as some of the sequences in vertebrates show similarity to both 7tm_GPCR_Srsx domain and 7tm_4 (olfactory) domain, within their transmembrane spanning regions (see Table S2). As the 7tm_GPCR_Srsx domain and 7tm_4 (olfactory) domain belong to the same Pfam clan (GPCR_A clan), we suggest that both srsx family and the 7tm_4 family perhaps shares a common origin, somewhere before the split of protostomes and deuterostomes. Furthermore, from a previous study [26], as well as from our phylogenetic analysis (Figure 3), it seems evident that *N. vectensis* have chordate like olfactory genes that have expanded within the cnidarian lineage, similar to the expansion of this family in deuterostomes. In contrast, the chordate-like olfactory receptors were lost in all protostomes, which may reflect the expansion of NemChRs in this lineage. Taken together, these results suggest that the common ancestor of the protostomes and deuterostomes may have had ancestral representatives for most of the deuterostome chemosensory gene families and the srw gene family (Figure 6). This suggestion is supported by our previous study, which argues that most of the chemosensory GPCRs and the *Rhodopsin* family GPCRs share a common origin, somewhere close to the divergence of the cnidarians from the eumetazoans [22].

In summary, we have performed a detailed mining of the nematode chemosensory gene families across 26 eukaryotic genomes and found that the srw gene family has putative orthologs across several phyla of protostomes. Furthermore, we here show that the srw gene family split from the large *Rhodopsin* family, possibly from the peptide and/or SOG subfamilies, somewhere close to divergence of the common ancestor of protostomes. Our results provide important insights into the evolutionary events of the GPCR genes that are responsible for sensing the environment.

## Supporting Information

Figure S1 **Phylogenetic trees showing closest related** ***Rhodopsin*** **subfamilies to the srw family in all four analyzed species (** ***C. elegans*** **,** ***N. vectensis*** **,** ***D. melanogaster*** **and** ***T. adhaerens*** **).** Olfactory like genes identified in *N. vectensis* is used as outgroup and subsequently rooted in all the trees. Posterior probabilities were shown for the major nodes in percentage. The *Rhodopsin* subfamily sequences included in the trees 1 to 4 were obtained using srw family sequences as queries in a BLASTP search against the *Rhodopsin* family repertoire in *C. elegans*, *D. melanogaster*, *N. vectensis* and *T. adhaerens*, respectively.
(PDF)

Figure S2 **Multiple alignment showing conserved regions between the novel srw members and the srws from** ***C. elegans*** **and** ***P. pacificus*** **.** The protein IDs of novel srw like sequences identified in the genomes of insects, mollusk and *S. mansoni* are highlighted in red.
(PDF)

Figure S3 **Flowchart describing the sequence analysis strategy used to identify the closest** ***Rhodopsin*** **subfamily to the srw family.**
(PDF)

Figure S4 **Schematic representation of HMM-HMM profile comparison scores between the** ***Rhodopsin*** **and NemChR subfamilies.** The scores within the parenthesis are HHsearch probability score in percentage and E-values between the query and the respective hit. Among the hits, peptide (pep), SOG and amines (amin) are colored. Hits with HHsearch probability scores that are less than 80% (relatively less homology) are highlighted in red. Empty cells indicate no further hits were found. The 19 NemChR HMM profiles were downloaded from the Pfam database. HMM models constructed using the multiple alignments of our sequence datasets include Ag_srw (*Anopheles gambiae*), Ac_srw (*Aplysia californica*), Ap_srw (*Acyrthosiphon pisum*), Dp_srw (*Daphnia pulex*), Am_srw (*Apis mellifera*), Lg_srw (*Lottia gigantea*), Ph_srw (*Pediculus humanus*), Pp_srw (*Pristionchus pacificus*), Sm_srw (*Schistosoma mansoni*) and Dros_srw (*Drosophila melanogaster* and *Drosophila willistoni*).
(PDF)

Table S1 **List of sequences identified with the highest alignment score against the 7tm_GPCR_srw (PF10324) domain.**
(DOCX)

Table S2 **List of sequences identified with srsx (7TM_GPCR_Srsx) domain.** The table contains a list of vertebrate olfactory receptors that had two significant Pfam HMM profile hits corresponding to 7TM_GPCR_Srsx domain (PF10320) and 7tm_4 domain (PF13853), within their transmembrane spanning regions. The domain spanning or envelope regions, i.e., residue coordinates on the sequence where the Pfam match has been probabilistically determined to lie is given for all the hits.
(DOCX)

Dataset S1 **List of all sequences identified in this study in FASTA format.**
(PDF)

## References

[1] Bargmann CI (2006) Comparative chemosensation from receptors to ecology. Nature 444: 295–301. 17108953 10.1038/nature05402

[2] Nei M, Niimura Y, Nozawa M (2008) The evolution of animal chemosensory receptor gene repertoires: roles of chance and necessity. Nat Rev Genet 9: 951–963. 19002141 10.1038/nrg2480

[3] Dryer L, Berghard A (1999) Odorant receptors: a plethora of G-protein-coupled receptors. Trends Pharmacol Sci 20: 413–417. 10498954 10.1016/s0165-6147(99)01381-4

[4] Mombaerts P (1999) Seven-transmembrane proteins as odorant and chemosensory receptors. Science 286: 707–711. 10531047 10.1126/science.286.5440.707

[5] Gaillard I, Rouquier S, Giorgi D (2004) Olfactory receptors. Cell Mol Life Sci 61: 456–469. 14999405 10.1007/s00018-003-3273-7 PMC11138504

[6] Buck L, Axel R (1991) A novel multigene family may encode odorant receptors: a molecular basis for odor recognition. Cell 65: 175–187. 1840504 10.1016/0092-8674(91)90418-x

[7] Liberles SD, Buck LB (2006) A second class of chemosensory receptors in the olfactory epithelium. Nature 442: 645–650. 16878137 10.1038/nature05066

[8] Pantages E, Dulac C (2000) A novel family of candidate pheromone receptors in mammals. Neuron 28: 835–845. 11163270 10.1016/s0896-6273(00)00157-4

[9] Dulac C, Axel R (1995) A novel family of genes encoding putative pheromone receptors in mammals. Cell 83: 195–206. 7585937 10.1016/0092-8674(95)90161-2

[10] Herrada G, Dulac C (1997) A novel family of putative pheromone receptors in mammals with a topographically organized and sexually dimorphic distribution. Cell 90: 763–773. 9288755 10.1016/s0092-8674(00)80536-x

[11] Zhao GQ, Zhang Y, Hoon MA, Chandrashekar J, Erlenbach I, et al (2003) The receptors for mammalian sweet and umami taste. Cell 115: 255–266. 14636554 10.1016/s0092-8674(03)00844-4

[12] Adler E, Hoon MA, Mueller KL, Chandrashekar J, Ryba NJ, et al (2000) A novel family of mammalian taste receptors. Cell 100: 693–702. 10761934 10.1016/s0092-8674(00)80705-9

[13] Bargmann CI (2006) Chemosensation in C. elegans. WormBook: 1–29. 10.1895/wormbook.1.123.1 PMC4781564 18050433

[14] Thomas JH, Robertson HM (2008) The Caenorhabditis chemoreceptor gene families. BMC Biol 6: 42. 18837995 10.1186/1741-7007-6-42 PMC2576165

[15] Rouquier S, Giorgi D (2007) Olfactory receptor gene repertoires in mammals. Mutat Res 616: 95–102. 17166524 10.1016/j.mrfmmm.2006.11.012

[16] Churcher AM, Taylor JS (2009) Amphioxus (Branchiostoma floridae) has orthologs of vertebrate odorant receptors. BMC Evol Biol 9: 242. 19804645 10.1186/1471-2148-9-242 PMC2764704

[17] Libants S, Carr K, Wu H, Teeter JH, Chung-Davidson YW, et al (2009) The sea lamprey Petromyzon marinus genome reveals the early origin of several chemosensory receptor families in the vertebrate lineage. BMC Evol Biol 9: 180. 19646260 10.1186/1471-2148-9-180 PMC2728731

[18] Raible F, Tessmar-Raible K, Arboleda E, Kaller T, Bork P, et al (2006) Opsins and clusters of sensory G-protein-coupled receptors in the sea urchin genome. Dev Biol 300: 461–475. 17067569 10.1016/j.ydbio.2006.08.070

[19] Dong D, Jones G, Zhang S (2009) Dynamic evolution of bitter taste receptor genes in vertebrates. BMC Evol Biol 9: 12. 19144204 10.1186/1471-2148-9-12 PMC2646699

[20] Johnstone KA, Ciborowski KL, Lubieniecki KP, Chow W, Phillips RB, et al (2009) Genomic organization and evolution of the vomeronasal type 2 receptor-like (OlfC) gene clusters in Atlantic salmon, Salmo salar. Mol Biol Evol 26: 1117–1125. 19221009 10.1093/molbev/msp027 PMC2668830

[21] Hashiguchi Y, Nishida M (2006) Evolution and origin of vomeronasal-type odorant receptor gene repertoire in fishes. BMC Evol Biol 6: 76. 17014738 10.1186/1471-2148-6-76 PMC1601972

[22] Nordstrom KJ, Sallman Almen M, Edstam MM, Fredriksson R, Schioth HB (2011) Independent HHsearch, Needleman—Wunsch-based, and motif analyses reveal the overall hierarchy for most of the G protein-coupled receptor families. Mol Biol Evol 28: 2471–2480. 21402729 10.1093/molbev/msr061

[23] Jekely G (2013) Global view of the evolution and diversity of metazoan neuropeptide signaling. Proc Natl Acad Sci U S A 110: 8702–8707. 23637342 10.1073/pnas.1221833110 PMC3666674

[24] Putnam NH, Srivastava M, Hellsten U, Dirks B, Chapman J, et al (2007) Sea anemone genome reveals ancestral eumetazoan gene repertoire and genomic organization. Science 317: 86–94. 17615350 10.1126/science.1139158

[25] Srivastava M, Begovic E, Chapman J, Putnam NH, Hellsten U, et al (2008) The Trichoplax genome and the nature of placozoans. Nature 454: 955–960. 18719581 10.1038/nature07191

[26] Churcher AM, Taylor JS (2011) The antiquity of chordate odorant receptors is revealed by the discovery of orthologs in the cnidarian Nematostella vectensis. Genome Biol Evol 3: 36–43. 21123836 10.1093/gbe/evq079 PMC3017388

[27] Punta M, Coggill PC, Eberhardt RY, Mistry J, Tate J, et al (2012) The Pfam protein families database. Nucleic Acids Res 40: D290–301. 22127870 10.1093/nar/gkr1065 PMC3245129

[28] Fredriksson R, Lagerstrom MC, Lundin LG, Schioth HB (2003) The G-protein-coupled receptors in the human genome form five main families. Phylogenetic analysis, paralogon groups, and fingerprints. Mol Pharmacol 63: 1256–1272. 12761335 10.1124/mol.63.6.1256

[29] Soding J (2005) Protein homology detection by HMM-HMM comparison. Bioinformatics 21: 951–960. 15531603 10.1093/bioinformatics/bti125

[30] Finn RD, Mistry J, Tate J, Coggill P, Heger A, et al (2010) The Pfam protein families database. Nucleic Acids Res 38: D211–222. 19920124 10.1093/nar/gkp985 PMC2808889

[31] Katoh K, Misawa K, Kuma K, Miyata T (2002) MAFFT: a novel method for rapid multiple sequence alignment based on fast Fourier transform. Nucleic Acids Res 30: 3059–3066. 12136088 10.1093/nar/gkf436 PMC135756

[32] Katoh K, Toh H (2008) Recent developments in the MAFFT multiple sequence alignment program. Brief Bioinform 9: 286–298. 18372315 10.1093/bib/bbn013

[33] Waterhouse AM, Procter JB, Martin DM, Clamp M, Barton GJ (2009) Jalview Version 2—a multiple sequence alignment editor and analysis workbench. Bioinformatics 25: 1189–1191. 19151095 10.1093/bioinformatics/btp033 PMC2672624

[34] Ronquist F, Teslenko M, van der Mark P, Ayres DL, Darling A, et al (2012) MrBayes 3.2: efficient Bayesian phylogenetic inference and model choice across a large model space. Syst Biol 61: 539–542. 22357727 10.1093/sysbio/sys029 PMC3329765

[35] Guindon S, Dufayard JF, Lefort V, Anisimova M, Hordijk W, et al (2010) New algorithms and methods to estimate maximum-likelihood phylogenies: assessing the performance of PhyML 3.0. Syst Biol 59: 307–321. 20525638 10.1093/sysbio/syq010

[36] Cummins SF, Erpenbeck D, Zou Z, Claudianos C, Moroz LL, et al (2009) Candidate chemoreceptor subfamilies differentially expressed in the chemosensory organs of the mollusc Aplysia. BMC Biol 7: 28. 19493360 10.1186/1741-7007-7-28 PMC2700072

[37] Robertson HM, Thomas JH (2006) The putative chemoreceptor families of C. elegans. WormBook: 1–12. 10.1895/wormbook.1.66.1 PMC4781013 18050473

