# Supplementary figures and images for: Insights into the Origin of Nematode Chemosensory GPCRs: Putative Orthologs of the Srw Family Are Found across Several Phyla of Protostomes
Source: PLoS One. 2014 Mar 24;9(3):e93048. doi: 10.1371/journal.pone.0093048 (PMC3963977; doi:10.1371/journal.pone.0093048)

## Figure S1

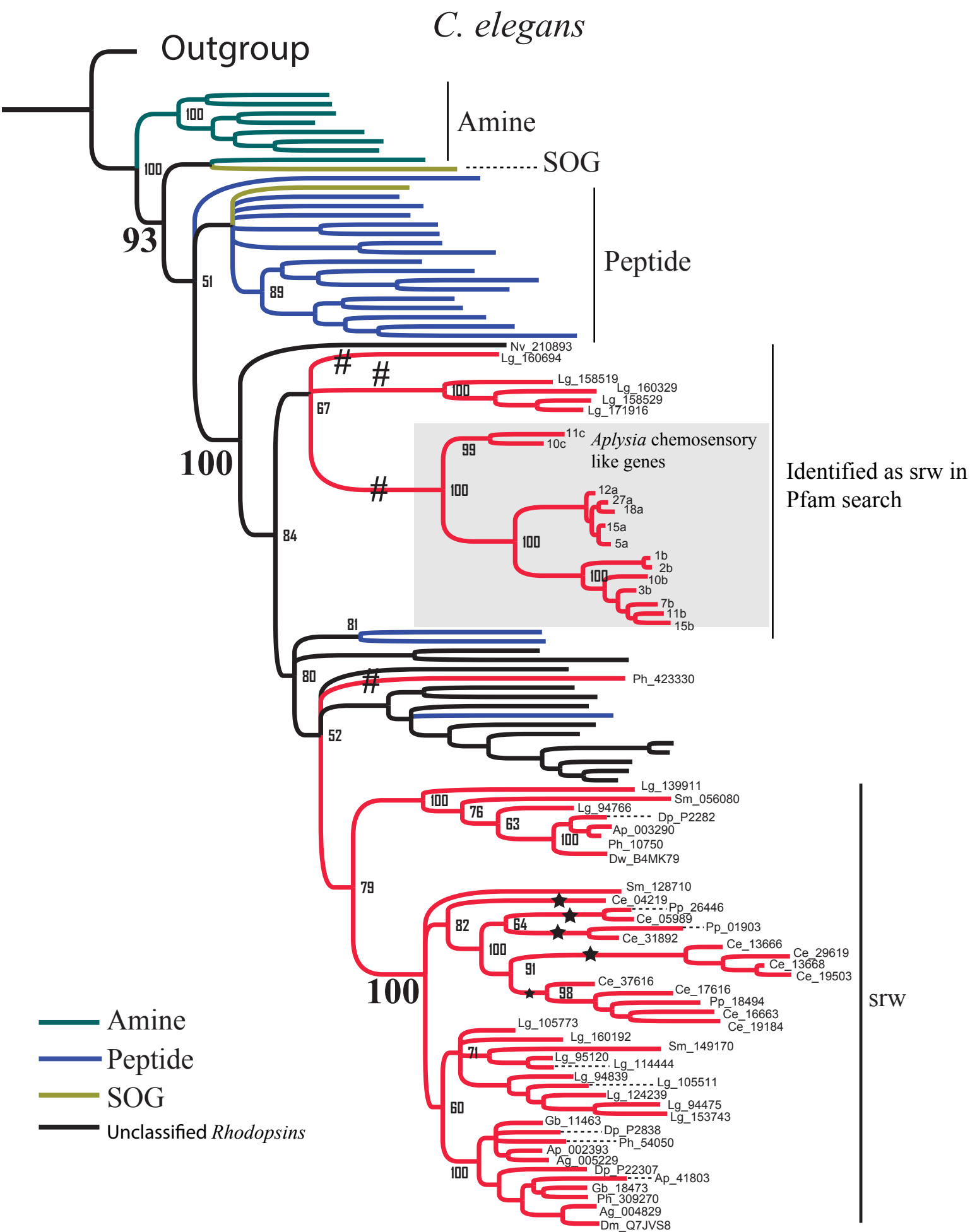

Figure S1 cont

*D. melanogaster*

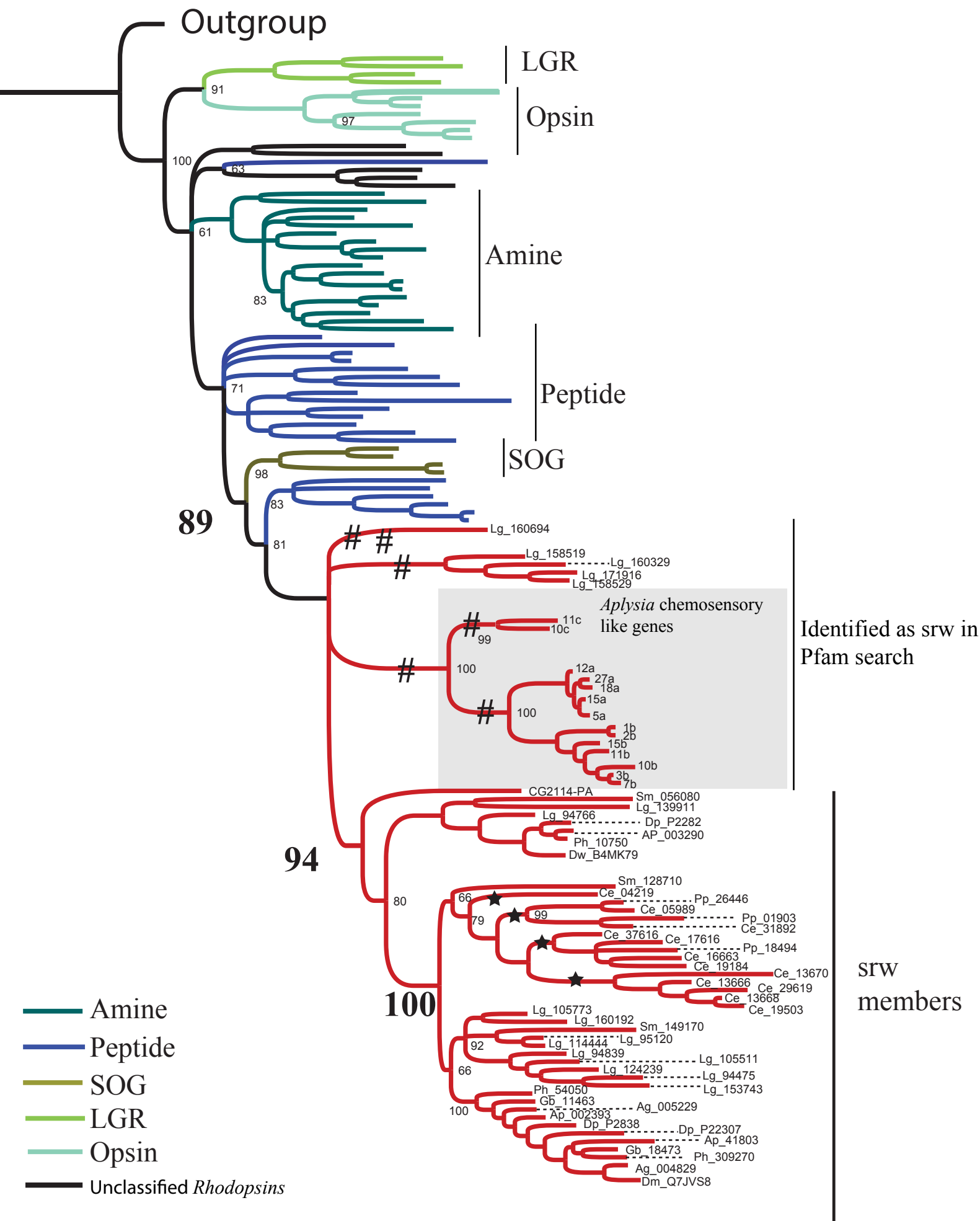

Figure S1 cont

*N. vectensis*

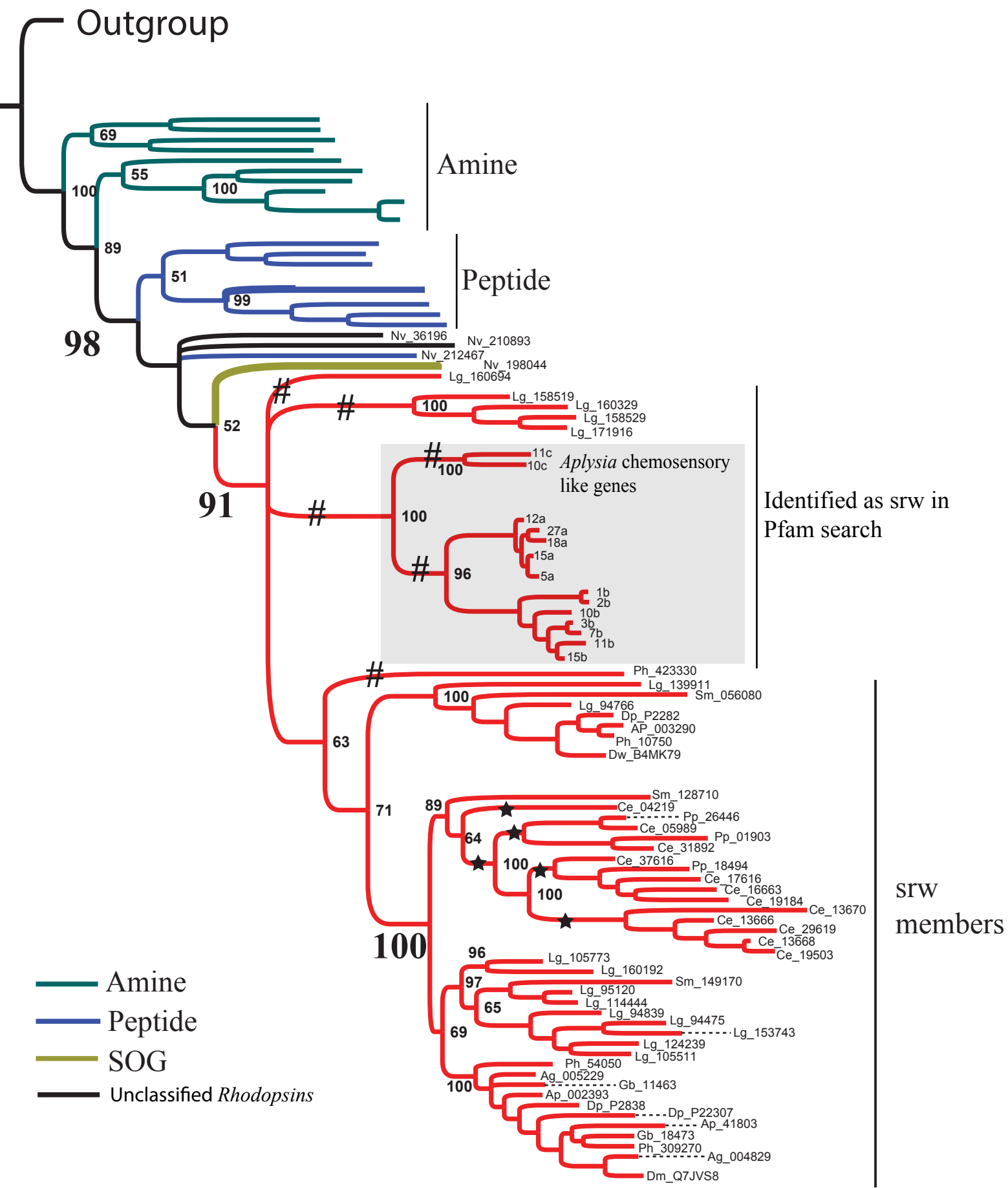

*T. adhearens*

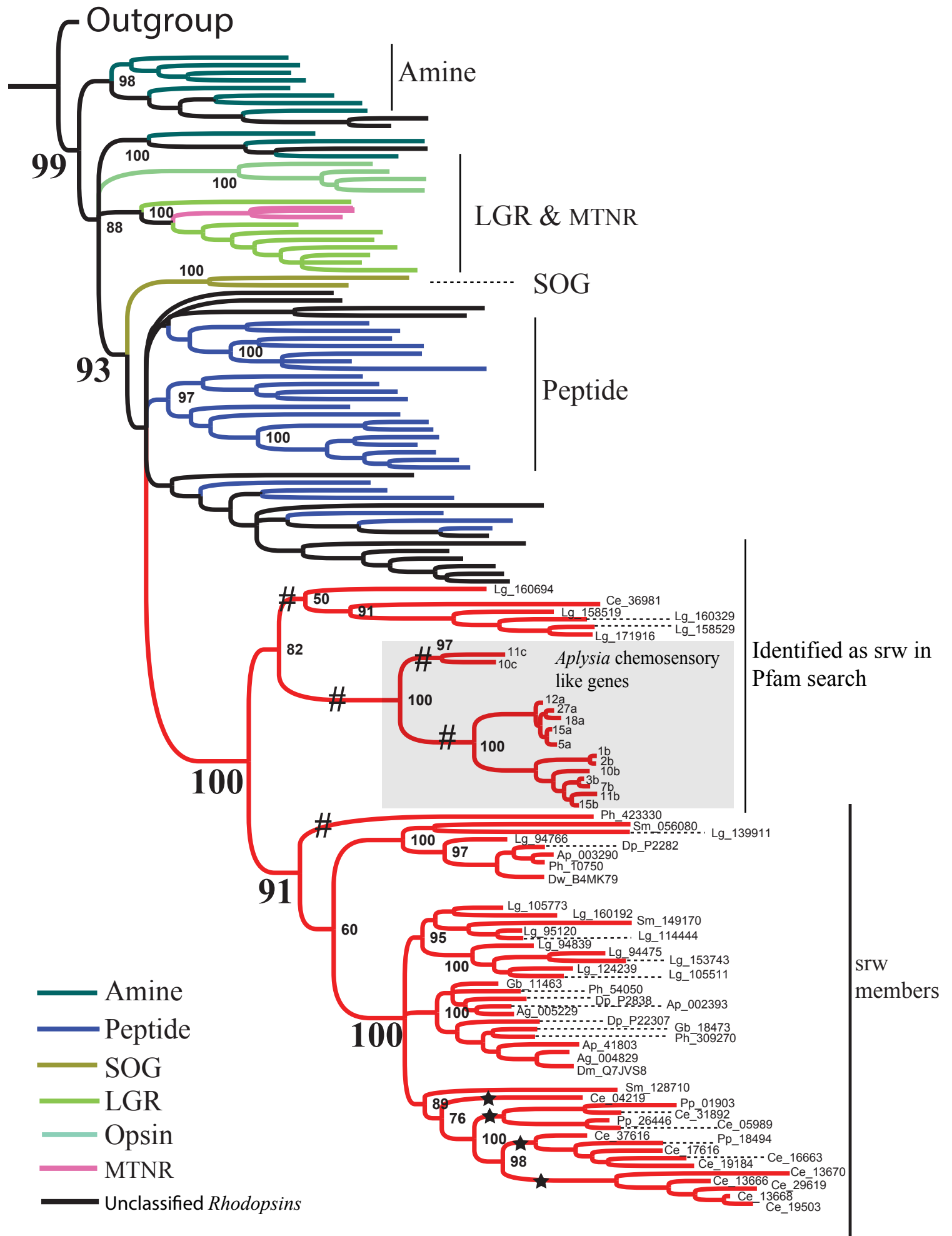

Supplement: Figure S1 — Phylogenetic trees showing closest related Rhodopsin subfamilies to the srw family in all four analyzed species ( C. elegans , N. vectensis , D. melanogaster and T. adhaerens ). Olfactory like genes identified in N. vectensis is used as outgroup and subsequently rooted in all the trees. Posterior probabilities were shown for the major nodes in percentage. The Rhodopsin subfamily sequences included in the trees 1 to 4 were obtained using srw family sequences as queries in a BLASTP search against the Rhodopsin family repertoire in C. elegans, D. melanogaster, N. vectensis and T. adhaerens, respectively. (PDF) [file pone.0093048.s001.pdf]

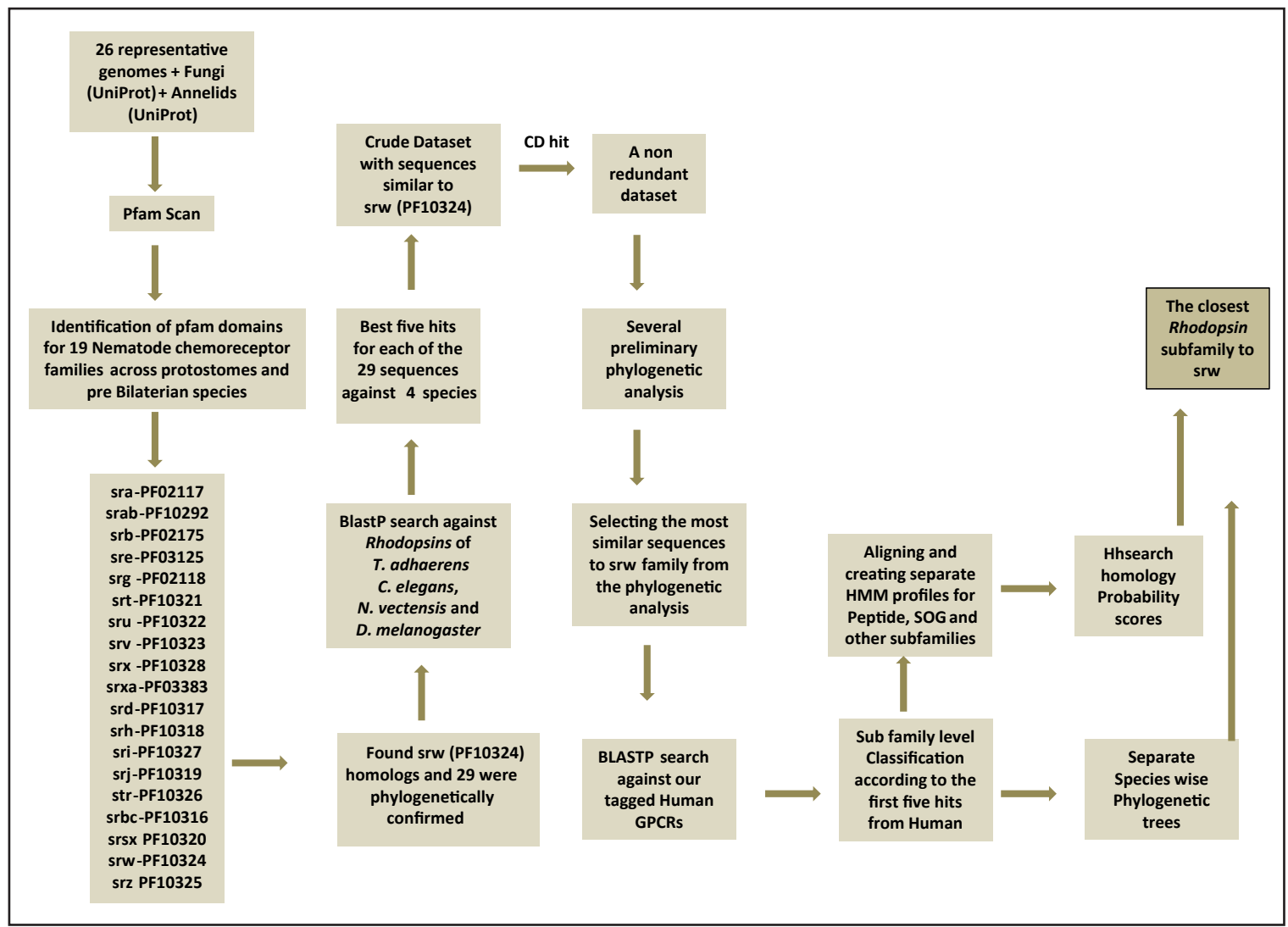

Supplement: Figure S3 — Flowchart describing the sequence analysis strategy used to identify the closest Rhodopsin subfamily to the srw family. (PDF) [file pone.0093048.s003.pdf]
