# Supplementary material for: Insights into the Origin of Nematode Chemosensory GPCRs: Putative Orthologs of the Srw Family Are Found across Several Phyla of Protostomes
Source: PLoS One. 2014 Mar 24;9(3):e93048. doi: 10.1371/journal.pone.0093048 (PMC3963977; doi:10.1371/journal.pone.0093048)
Supplement: Figure S2 — Multiple alignment showing conserved regions between the novel srw members and the srws from C. elegans and P. pacificus . The protein IDs of novel srw like sequences identified in the genomes of insects, mollusk and S. mansoni are highlighted in red. (PDF) [file pone.0093048.s002.pdf]

Figure S2 Multiple alignment showing conserved regions between the novel srw members and the srws from C. elegans and P. pacificus.

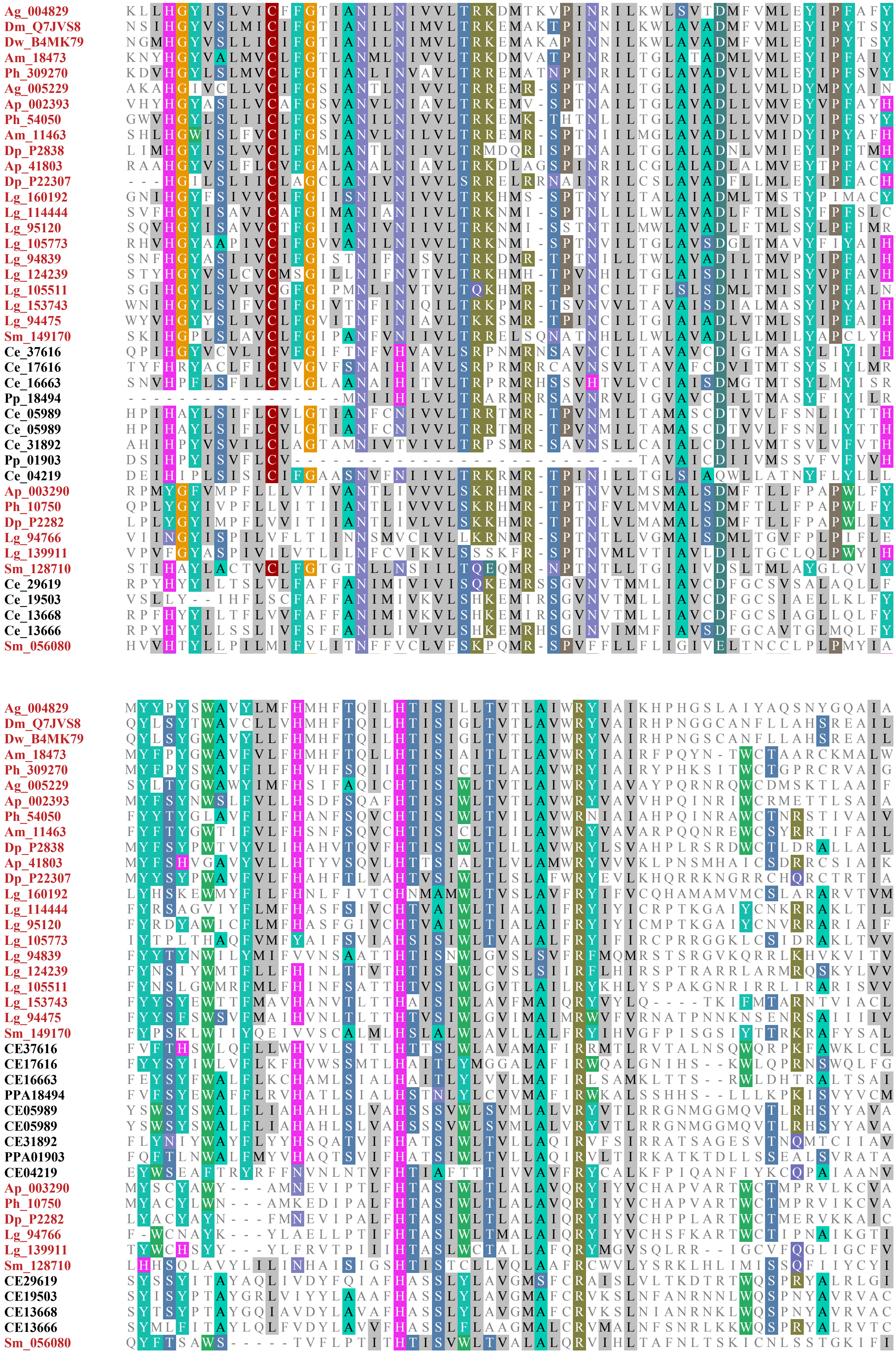

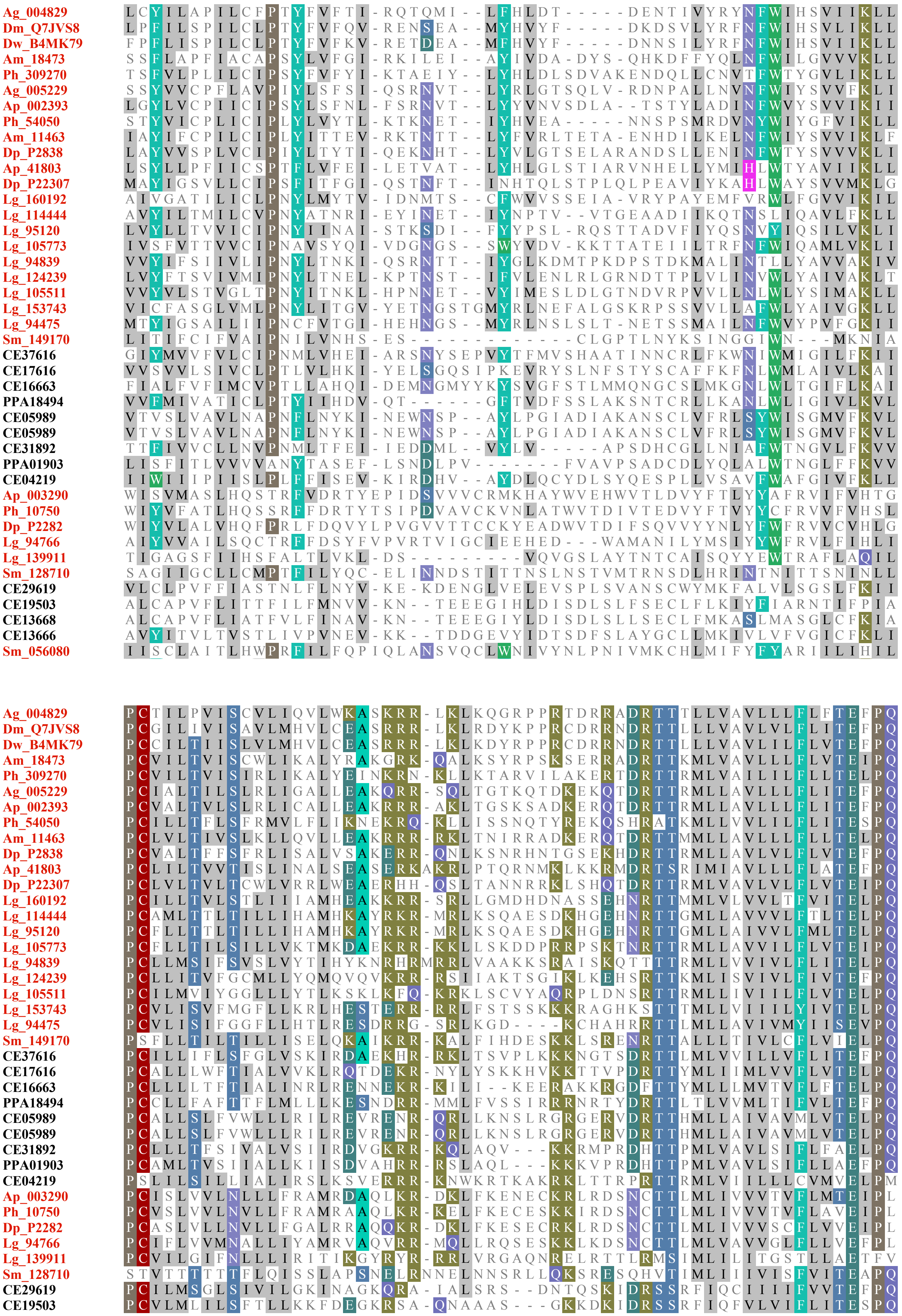
Ag_004829
Dm_Q7JVS8
Dw_B4MK79
Am_18473
Ph_309270
Ag_005229
Ap_002393
Ph_54050
Am_11463
Dp_P2838
Ap_41803
Dp_P22307
Lg_160192
Lg_114444
Lg_95120
Lg_105773
Lg_94839
Lg_124239
Lg_105511
Lg_153743
Lg_94475
Sm_149170
CE37616
CE17616
CE16663
PPA18494
CE05989
CE05989
CE31892
PPA01903
CE04219
Ap_003290
Ph_10750
Dp_P2282
Lg_94766
Lg_139911
Sm_128710
CE29619
CE19503
CE13668
CE13666
Sm_056080

```
CE13668    PCLLMLVLSLFLLRRIDEGKQSA-QNAAAN---GKKDKIDRSSRFIQFVLVVFLITESPQ
CE13666    PCTLMLILSILLLQKMDEGKRSS-VPINRN---KRVDQLDRSSQLIQTILIVFLITEVPQ
Sm_056080  PCGILIALTIILVIKMHNIIKLR-KRLGLRTTKKSSINPQAISRMLIVVLIKFIAMHLPN
```

```
Ag_004829  GILGLMSGILEFFRRCYGLFGEVMDLLALINAAIGFVLYGLMSKQFRTSFKSVF
Dm_Q7JVS8  GLLGLLSGVMEFFAHCYPPFGELMDLLALINAAVGFVLYGLMSKQFRTTFRSLF
Dw_B4MK79  GLLGLLSGILKFFEHCYPPFGEIVDLLALINAAVGFVLYGFMSKQFRTTFRSLF
Am_18473   GILGLLSGVLGFFRNCYHNFGEVMDILALLNGAINFILYCSMSRQFRTTFGQLF
Ph_309270  GILGLLSGILGFFRNCYNLFGEIMDILALVNSAINFILYCFMSRQFRQTFEKVF
Ag_005229  GILGLLSAVLKFFFNCYLKLGDVMDVLALVNSAINFILYCSMSRQFRSTFNDLF
Ap_002393  GILGLLTLLLGFFQDCYQNMGEVMDMLALVNSAINFILYCVMSRQFRNTFSLLF
Ph_54050   GILGLLSYSLGFYNSCYHPLGDVLDFLALFNSSTNFMIYCTMSRQFRVTFNLLF
Am_11463   GILGLFSVLLGFFKACYLMLGDVIDMLTLVNSAINFILYCTMSRQFRKTFNELF
Dp_P2838   GILALLSGILGFFTNCYRHLGELTDILALINGAINFLLYCVMSRQFRQTFSRIF
Ap_41803   GILGLLSGILGFFQTCYSLFGELMDMLALLNASLNFVFYCCMSKQFRVAFGQLF
Dp_P22307  GVMALLSGVLGFFIKCYSTMGEILDLLALLNSAINFLLYCSMSRQFR-------
Lg_160192  GILALISAV-NFFSRVYVNLGDLMDLLVLINSAVNFILYCIMSQQFRNTFKSLF
Lg_114444  GILTLMNIFI-FQWYVYDKLGDLIDTMALMNNSINFVLYCSMSKQFRDTFTEIF
Lg_95120   GILTLMTLF-ELQFYVYNNLGDLVDVMALINNSINFVLYCTMSKQFRDTFVKVF
Lg_105773  GILNLISGI-EFFDNVYTPLGDLMDILALINNGINFILYCTMSKQFRDTFIKLF
Lg_94839   GILILVTAAI-FHNNVYNLLGDLMDFIALLNNAINFVLYCSMSQQFRSRFIEMY
Lg_124239  GILIVLSAC-KFFETVYLPLGDIMDIVALVNNAVNFVLYCTMSTKFRETFLRQY
Lg_105511  GILIILSVT-SFFHFIYMPLGDLMDMMALINNGVNFILYCSMSRDFRLTLMDLL
Lg_153743  SVLVILSVI-FFFENVYYLLADILDMAALINNAINFVLYCIMSQQFRGHLMEMW
Lg_94475   SILVVLCAC-VFFVDVYLPLSDLIDAIALINSAINFVMYCTMSQQFR-------
Sm_149170  GILVTCIHL-IFEEKVYQHLGDLLDFLTLLNESISFIIYTTMSNQFRQTFCNIF
CE37616    GIISILCAIFTVHRYLYFYIGDVLDLLSLVNSSVNFVLYCVMSSRYRQTFWEVI
CE17616    GFLALLNGLYTVNIYIYKNLSELLDFLSLINCSVDFLLYCVMSSRYRQTFGHML
CE16663    GIMAILNALFTFHQMVYLNLADVLDLLSLINCYVAFLVYSFTSSRYRQTLFSLL
PPA18494   GMVAVLNGVYTVYNHIYSIFGDMLDLLSLINCYVGFIAYGFLCSKYRQTFVMLM
CE05989    GIMAVLSGMCSFRIYIYNNLGDILDLFSLCGSCCSFIIYCSMSGQFRNEFHRVF
CE05989    GIMAVLSGMCSFRIYIYNNLGDILDLFSLCGSCCSFIIYCSMSGQFRNEFHRVF
CE31892    GVLHVFNAIFTFYDKIYIHLGDVMDVLSLLNSAVNFIIYCAMSRKFRAVFIQIF
PPA01903   GVLHIGKVVFTFHQQIYQPLGNVMDLLSLVNSAVNFIIYCAMSRNKLQYGADFD
CE04219    GILNLCVAIYGFGNRYYDPVGNLMEMLTLLYSSVSFVLYCTMSNEYLSTFRALF
Ap_003290  AVVTVLHIISSLDYSVANLLVLFTNFFIIVSYPINFAIYCGMSRQFRETFKELF
Ph_10750   AVITVLHIISSLDYNIANVLILFSNFFIIVSYPINFAIYCGMSRQFRETFKELF
Dp_P2282   AVLTVLHIFDALDYSVINVLILFTNFFISLSYPINFAIYCGMSRQFRETFQGLF
Lg_94766   GIIMILLIVSNISKETFQILSIFSNLFILLSYPFNFFIYCGMSKQFRETFKRLF
Lg_139911  AILLIIQTSQEADPTNVSKSMVISNFLILFTFPLNFLFYFLMSSRFRRHFVNCY
Sm_128710  GVFNALVAIKGFLHTIYLPLGDLLDLLVLLNSSTNFILYCAMSQVFRVNFVNLL
CE29619    GFFSVVGGLSI-YINYFQFFSVFTNLLAYFNTTTSFIIYSTLSSKFRKLFVQLF
CE19503    GVFKILSGIMM-YINYFQNLTIFINILAFFNSTTSFIIYSSLSAKFRKIFAQLF
CE13668    GVFSILGGFMI-YINYFQNLSIFMNILAFFNTTTSFIIYSSLSAKFRRIFAQLF
CE13666    GVFSIIGGIEV-YLNYYQNLTIFMNVLSFFNTTTSFIIYSSLSAKFRRIFAQTF
Sm_056080  AIVLTIYVLRKLIDDYLGKAVILCNLVILVSYQLNFVIYYVMSTQFKETFNNLC
```
