# Supplementary material for: Insights into the Origin of Nematode Chemosensory GPCRs: Putative Orthologs of the Srw Family Are Found across Several Phyla of Protostomes
Source: PLoS One. 2014 Mar 24;9(3):e93048. doi: 10.1371/journal.pone.0093048 (PMC3963977; doi:10.1371/journal.pone.0093048)
Supplement: Figure S4 — Schematic representation of HMM-HMM profile comparison scores between the Rhodopsin and NemChR subfamilies. The scores within the parenthesis are HHsearch probability score in percentage and E-values between the query and the respective hit. Among the hits, peptide (pep), SOG and amines (amin) are colored. Hits with HHsearch probability scores that are less than 80% (relatively less homology) are highlighted in red. Empty cells indicate no further hits were found. The 19 NemChR HMM profiles were downloaded from the Pfam database. HMM models constructed using the multiple alignments of our sequence datasets include Ag_srw (Anopheles gambiae), Ac_srw (Aplysia californica), Ap_srw (Acyrthosiphon pisum), Dp_srw (Daphnia pulex), Am_srw (Apis mellifera), Lg_srw (Lottia gigantea), Ph_srw (Pediculus humanus), Pp_srw (Pristionchus pacificus), Sm_srw (Schistosoma mansoni) and Dros_srw (Drosophila melanogaster and Drosophila willistoni). (PDF) [file pone.0093048.s004.pdf]

	Query	1	2	3	4	5
	sra	srab (96.7; 1.8E-08)	srb (75.4; 0.0013)			
Sra Superfamily	srab	sra (96.7; 1.9E-08)	srb (96.1;1.5E-07)	sre (90.5;7.4E-05)		
	srb	srab (96.2; 1.3E-07)	sra (75.8; 0.0013)			
	sre	srab (90.4;7.5E-05)	sra (28.1; 0.034)			
	srd	str (98.5; 1.1E-13)	srj (98.2; 2.7E-12)	srh (96.8; 1.1E-08)	sri (96.0; 2.3E-07)	
Str Superfamily	srh	sri (99.1; 5E-17)	str (97.2; 1.5E-09)	srd (96.8; 1.1E-08)	srj (94.0; 8E-06)	
	sri	srh (99.1; 6.8E-17)	srd (96.0; 2.5E-07)	str (94.8; 2.7E-06)	srj (94.8; 2.8E-06)	
	srj	str (99.8; 1.4E-27)	srd (98.2; 2.9E-12)	sri (94.8; 2.8E-06)	srh (93.9; 8.4E-06)	
	str	srj (99.8; 1.3E-27)	srd (98.5; 1.3E-13)	srh (97.2; 1.1E-08)	sri (94.8; 2.6E-06)	
	srg	srv (96.4; 5.8E-08)	sru (90.4; 7.5E-05)	srx (32.5; 0.025)		
	sru	srg (90.2; 8.3E-05)				
Srg Superfamily	srv	srg (96.4; 6.7E-08)	srx (87.2; 0.00021)			
	srx	srv (87.4; 0.0002)	Nv_Amin (77.4; 0.0011)	srt (76.8; 0.0011)	Ce_Pep (74.5; 0.0014)	Ta_Amin 73.(73.8; 0.0015)
	srt	srx (76.6; 0.0012)	srv (13.2; 0.12)			
	srxa	srx (18.4; 0.07)				
	srbc	Ce_Pep (18.4; 0.07)	srsx (14.3; 0.1)	srx (13.6; 0.11)		
Individual families	srsx	Ce_Pep (95.7; 5.4E-07)	Nv_Amin (95.6; 6.7E-07)	Ta_Pep (92.1; 3.3E-05)	Dm_pep (92.0; 3.6E-05)	Nv_pep (89.8; 9.7E-05)
	srw	Ce_Pep (95.6; 6.4E-07)	Dm_Sog (93.3; 1.5E-05	Dm_Pep(92.9; 2E-05)	Nv_Pep (64.4; 0.0034)	Nv_Sog (26.5; 0.038)
	srz	No hits				
	Ag_srw	No hits other than srw families				
	Ac_srw	Ce_Pep (96.0;2.3E-07)	Dm_pep (95.6; 5.9E-07)	Nv_pep (91.2; 5.3E-05)	Nv_Amin (87.7; 0.00019)	Nv_sog (87.0; 0.00022)
	Ap_srw	Ce_Pep (96.9;1E-08)	Ce_Sog (96.6; 3.4E-08)	Dm_Pep (96.4; 6.2E-08)	Nv_pep (94.4; 4.5E-06)	Nv_sog (92.8; 2.1E-05)
srw	Dp_srw	Ce_Pep (97.9;2E-11)	Dm_Pep (97.8; 4.2E-11)	Ce_Sog (97.8; 5.3E-11)	Nv_pep (96.6; 2.8E-08)	Ta_Pep (96.1; 1.5E-07)
	Dros_srw	Ce_Pep (97.2; 2.5E-09)	Dm_Pep (96.4; 6.6E-08)	Ce_Sog (95.4; 9.5E-07)	Ce_Amin (92.8; 2.2E-05)	Nv_pep (92.6; 2.4E-05)
	Am_srw	Dm_Pep (95.2; 1.4E-06)	Ce_Sog (95.1; 1.6E-06)	Ce_Pep (94.9; 2.3E-06)	Ce_Amin (90.1; 8.6E-05)	Nv_pep (65.8; 0.0031)
	Lg_srw	Ce_Pep (98.5; 1.1E-13)	Dm_Pep (98.4; 3.9E-13)	Ce_Sog (98.1; 5.8E-12)	Nv_pep (97.3; 1.3E-09)	Nv_sog (96.9; 8.2E-09)
	Ph_srw	Ce_Pep (97.5; 4.2E-10)	Ce_Sog (97.4; 5.4E-10)	Nv_pep (96.9; 8.7E-09)	Dm_Pep (96.8; 1.6E-08)	Nv_Amin (95.6; 6.4E-07)
	Pp_srw	Ce_Sog (55.1; 0.0064)	Ce_Pep (52.0; 0.0078)	Dm_Pep (21.7; 0.054)		
	Sm_srw	Ce_Pep(48.8; 4.2E-10)	Dm_Pep (32.2; 0.026)	Ce_Sog (26.0; 0.039)		
