# Supplementary material for: Insights into the Origin of Nematode Chemosensory GPCRs: Putative Orthologs of the Srw Family Are Found across Several Phyla of Protostomes
Source: PLoS One. 2014 Mar 24;9(3):e93048. doi: 10.1371/journal.pone.0093048 (PMC3963977; doi:10.1371/journal.pone.0093048)
Supplement: Table S1 — List of sequences identified with the highest alignment score against the 7tm_GPCR_srw (PF10324) domain. (DOCX) [file pone.0093048.s005.docx]

**Table S1.** List of sequences identified with the highest alignment score against the 7tm_GPCR_srw (PF10324) domain.

| **Species** | **Protein ID** | **7TM_GPCR_SRW domain spanning or envelope regions** |
| --- | --- | --- |
| *Anopheles gambiae* | Ag_004829 | 127-348 (3.1e-19), 364-456 (8.2e-10) |
|  | Ag_005229 | 57-395 (1.7e-40) |
| *Acyrthosiphon pisum* | Ap_003290 | 53-367 (2.5e-22) |
|  | Ap_002393 | 38-362 (7.6e-41) |
|  | Ap_41803 | 80-391 (5.7e-32) |
| *Apis mellifera* | Am_18473 | 91-408 (3.2e-33) |
|  | Am_11463 | 28-359 (1.3e-37) |
| *Drosophila melanogaster* | Dm_Q7JVS8 | 78-396 (2.1e-31) |
| *Drosophila willistoni* | Dw_B4MK79 | 35-351 (3.1e-30) |
| *Pediculus humanus* | Ph_10750 | 10-324 (6.8e-22) |
|  | Ph_309270 | 90-406 (6.3e-39) |
|  | Ph_54050 | 8-311 (1.4e-31) |
|  | Ph_423330 | 66-327 (1.1e-12) |
| *Daphnia pulex* | Dp_P2838 | 19-276 (1e-29), 286-382 (8e-14) |
|  | Dp_P2282 | 19-332 (6.2e-24) |
|  | Dp_P22307 | 3-323 (1.6e-31) |
| *Lottia gigantea* | Lg_94839 | 11-315 (5.1e-33) |
|  | Lg_105511 | 31-337 (1.9e-28) |
|  | Lg_124239 | 24-334 (3.5e-35) |
|  | Lg_153743 | 46-353 (5.1e-32) |
|  | Lg_94475 | 14-305 (9.9e-38) |
|  | Lg_160192 | 14-321 (4.1e-38) |
|  | Lg_114444 | 17-336 (2.4e-38) |
|  | Lg_95120 | 20-338 (3e-42) |
|  | Lg_105773 | 26-325 (1.6e-42) |
|  | Lg_94766 | 11-323 (2.6e-27) |
|  | Lg_139911 | 56-372 (4.1e-11) |
|  | Lg_160329 | 34-332 (1.5e-14) |
|  | Lg_171916 | 116-420 (4.5e-22) |
|  | Lg_160694 | 40-344 (1.7e-19) |
|  | Lg_158519 | 179-484 (5.8e-17) |
|  | Lg_158529 | 130-429 (5.5e-21) |
| *Aplysia californica* | 5a (AASC01117380) | 48-352 (5.6e-16) |
|  | 12a (AASC01093420) | 1-269 (1.1e-13) |
|  | 15a (AASC01194155) | 61-365 (3.2e-18) |
|  | 18a (AASC01187084) | 1-270 (7.9e-14) |
|  | 27a (AASC01105652) | 60-365 (3.8e-17) |
|  | 1b (AASC01147398) | 33-335 (2.9e-20) |
|  | 2b (AASC01177837) | 34-336 (3.3e-23) |
|  | 3b (AASC01132996) | 33-336 (8.3e-22) |
|  | 7b (AASC01137539) | 34-336 (2.5e-15) |
|  | 9b (AASC01123121) | 35-338 (3.8e-15) |
|  | 10b (AASC01191494) | 35-338 (2.2e-17) |
|  | 11b (AASC01159697) | 35-336 (9.6e-15) |
|  | 12b (AASC01159697) | 33-336 (2.2e-16) |
|  | 14b (AASC01064969) | 34-336 (7e-19) |
|  | 15b (AASC01189496) | 35-336 (2.8e-16) |
|  | 16b (AASC01202310) | 34-338 (2.7e-16) |
|  | 19b (AASC01182788) | 35-336 (2.7e-15) |
|  | 20b (AASC01152348) | 36-336 (3.9e-15) |
|  | 23b (AASC01196016) | 33-334 (5.2e-16) |
|  | 24b (AASC01025013) | 31-332 (1.1e-15) |
|  | 27b (AASC01064229) | 31-332 (4e-15) |
|  | 29b (AASC01193148) | 33-336 (2e-14) |
|  | 30b (AASC01085458) | 33-335 (2e-20) |
|  | 31b (AASC01201741) | 33-335 (3.3e-18) |
|  | 34b (AASC01091901) | 35-335 (1.5e-18) |
|  | 37b (AASC01203463) | 31-338 (9.6e-16) |
|  | 38b (AASC01160578) | 34-336 (7.3e-17) |
|  | 10c (AASC01111921) | 42-347 (1.4e-09) |
|  | 11c (AASC01161139) | 38-348 (2.5e-12) |
| *Schistosoma mansoni* | Sm_128710 | 76-217 (1.8e-10), 365-485 (6.7e-09) |
|  | Sm_149170 | 55-203 (2.2e-14), 289-379 (6.9e-11) |
|  | Sm_056080 | 103-390 (1.5e-06) |
| *Nematostella vectensis* | Nv_210893 | 12-164 (2.6e-10) |
| *Dictyostelium fasciculatum* | Df_F4PUK6 | 3-143 (1.9e-06) |

Note: We follow the same renaming pattern for the *A. californica* genes as previously used [30]. Accession numbers for *A. californica* are shown within the parenthesis. According to the Pfam database documentation the envelope coordinates represents the region on the sequence where the Pfam match has been probabilistically determined to lie.
