# Supplementary material for: Insights into the Origin of Nematode Chemosensory GPCRs: Putative Orthologs of the Srw Family Are Found across Several Phyla of Protostomes
Source: PLoS One. 2014 Mar 24;9(3):e93048. doi: 10.1371/journal.pone.0093048 (PMC3963977; doi:10.1371/journal.pone.0093048)
Supplement: Table S2 — List of sequences identified with srsx (7TM_GPCR_Srsx) domain. The table contains a list of vertebrate olfactory receptors that had two significant Pfam HMM profile hits corresponding to 7TM_GPCR_Srsx domain (PF10320) and 7tm_4 domain (PF13853), within their transmembrane spanning regions. The domain spanning or envelope regions, i.e., residue coordinates on the sequence where the Pfam match has been probabilistically determined to lie is given for all the hits. (DOCX) [file pone.0093048.s006.docx]

**Table S2.** List of sequences identified with srsx (7TM_GPCR_Srsx) domain

| **Species** | **Protein ID** | **Domain spanning or envelope regions** | | |
| --- | --- | --- | --- | --- |
|  |  | **7TM_GPCR_SRSX**  **(SRSX family of NEMCHRs)** | **7tm_4**  **(Olfactory receptor)** | **7tm_1 (*Rhodopsin* family)** |
| *Homo sapiens* | Hs_OR51C1P^#^ | 35-307 (3.4e-08) | 140-285 (6.2e-29) | - |
|  | Hs_OR6V1^#^ | 33-137 (1.1e-05) | 137-281 (1.2e-36) | - |
|  | Hs_OR51F2^#^ | 49-298 (4.3e-07) | 154-299 (2.5e-27) | - |
|  | Hs_OR51H1P^#^ | 37-255 (4.2e-06) | 145-288 (1.3e-23) | - |
|  | Hs_OR10H2^#^ | 35-306 (3.4e-05) | 139-284 (1.9e-37) | - |
|  | Hs_OR52A5^#^ | 37-310 (3.2e-07) | 139-288 (1.5e-27) | - |
|  | Hs_OR52M1^#^ | 37-152 (9.4e-09) | 141-288 (6e-27) | - |
|  | Hs_OR2Y1^#^ | 35-224 (1.5e-06) | 139-282 (3.9e-42) | - |
|  | Hs_OR51M1^#^ | 47-310 (3.6e-07) | 152-297 (3.8e-28) | - |
|  | Hs_OR1I1^#^ | 35-209 (6.6e-08) | 139-283 (4.2e-42) | - |
|  | Hs_OR52L1^#^ | 53-192 (9.6e-05) | 158-303 (1.5e-30) | - |
|  | Hs_OR56B4^#^ | 41-176 (2.1e-08) | 146-289 (1.3e-21) | - |
|  | Hs_OR51D1^#^ | 48-295 (9.1e-06) | 153-297 (3e-27) | - |
| *Mus musculus* | Mm_Olfr631^#^ | 36-308 (1.2e-06 | 141-286 (5.4e-29) | - |
|  | Mm_Olfr1381^#^ | 35-214 (3.3e-05) | 139-282 (5.3e-39) | - |
|  | Mm_Olfr432^#^ | 35-304 (2.3e-06) | 139-282 (5.5e-35) | - |
|  | Mm_Olfr1344^#^ | 37-222 (8.5e-07) | 142-288 (1.2e-40) | - |
|  | Mm_Olfr117^#^ | 34-215 (1.2e-05) | 138-282 (2.9e-37) | - |
|  | Mm_Olfr68^#^ | 37-271 (6.3e-05) | 142-288 (7.5e-26) | - |
|  | Mm_Olfr46^#^ | 43-313 (3.6e-06) | 147-291 (5.9e-41) | - |
|  | Mm_Olfr1361^#^ | 40-159 (3.5e-05) | 144-288 (1.3e-41) | - |
|  | Mm_Olfr554^#^ | 37-151 (2.3e-09) | 141-288 (6.6e-28) | - |
|  | Mm_Olfr553^#^ | 44-157 (1.5e-09) | 149-295 (4.1e-29) | - |
|  | Mm_Olfr551^#^ | 37-151 (3.1e-09) | 144-286 (1.1e-32) | - |
|  | Mm_Olfr550^#^ | 36-151 (2.2e-09) | 141-286 (4.5e-25) | - |
|  | Mm_Olfr10^#^ | 35-150 (5e-07) | 139-282 (1.2e-41) | - |
|  | Mm_Olfr684^#^ | 37-308 (7e-08) | 142-287 (9.6e-25) | - |
|  | Mm_Olfr94^#^ | 109-219 (4.8e-09) | 213-358 (1e-34) | - |
|  | Mm_Olfr1384^#^ | 34-149 (5.1e-06) | 138-281 (1.6e-38) | - |
|  | Mm_Olfr564^#^ | 38-309 (9.1e-06) | 142-287 (2.3e-32) | - |
|  | Mm_Olfr653^#^ | 41-179 (1.5e-09) | 146-291 (2.9e-32) | - |
|  | Mm_Olfr53^#^ | 36-301 (3.8e-06) | 140-284 (7.4e-42) | -- |
|  | Mm_Olfr61^#^ | 43-209 (2.1e-06) | 147-291 (7.8e-43) | - |
|  | Mm_Olfr45^#^ | 36-176 (3.4e-07) | 140-284 (3.4e-41) | - |
|  | Mm_Olfr577^#^ | 38-310 (3.6e-06) | 144-288 (2.9e-28) | - |
|  | Mm_Olfr1383^#^ | 35-286 (0.00017) | 139-282 (3.6e-38) | - |
|  | Mm_Olfr123^#^ | 33-143 (4.4e-05) | 137-281 (8.1e-39) | - |
|  | Mm_Olfr1385^#^ | 35-214 (7e-06) | 139-282 (1.5e-43) | - |
|  | Mm_Olfr1391^#^ | 35-235 (9.3e-05) | 139-282 (1.9e-41) | - |
|  | Mm_Olfr685^#^ | 38-217 (6.6e-08) | 146-288 (7.3e-31) | - |
|  | Mm_Olfr107^#^ | 32-179 (1.3e-06) | 136-280 (3.8e-38) | - |
|  | Mm_Olfr615^#^ | 43-167 (9.7e-11) | 148-293 (2.7e-28) | - |
|  | Mm_Olfr606^#^ | 37-308 (6.7e-08) | 144-287 (1.1e-27) | - |
|  | Mm_Olfr608^#^ | 36-308 (6.9e-07) | 135-286 (1.8e-27) | - |
|  | Mm_Olfr978^#^ | 33-171 (0.0001) | 138-280 (7.5e-47) | - |
|  | Mm_Olfr541^#^ | 37-298 (9.2e-07) | 141-285 (3.6e-43) | - |
|  | Mm_Olfr686^#^ | 37-229 (4.6e-09) | 144-287 (3.1e-28) | - |
|  | Mm_Olfr611^#^ | 37-309 (2.9e-10) | 142-287 (2.4e-27) | - |
|  | Mm_Olfr632^#^ | 39-307 (4.2e-10) | 144-289 (2.5e-27) | - |
|  | Mm_Olfr633^#^ | 35-158 (2.1e-09) | 141-285 (3.1e-29) | - |
|  | Mm_Olfr603^#^ | 36-305 (5.5e-06) | 140-284 (3.4e-41) | - |
|  | Mm_Olfr97^#^ | 33-303 (1.6e-05) | 137-281(3.2e-39) | - |
|  | Mm_Olfr1367^#^ | 37-150 (3.4e-06) | 141-285 (4.7e-45) | - |
|  | Mm_Olfr1387^#^ | 35-141 (3.2e-05) | 139-282 (3.6e-41) | - |
|  | Mm_Olfr609^#^ | 34-170 (5.1e-09) | 139-284 (1.2e-28) | - |
|  | Mm_Olfr1388^#^ | 35-154 (1.5e-06) | 139-282 (2.2e-41) | - |
| *Gallus gallus* | Gg_F1NTA9^#^ | 35-149 (1.2e-07) | 139-283 (5.2e-45) |  |
| *Xenopus tropicalis* | Xt_or51g2# | 39-162 (5.9e-09) | 144-289 (8.6e-32) | - |
| *Nematostella vectensis* | Nv_205247 | 37-167 (8.3e-05) | - | - |
| *Trichoplax adhaerens* | Ta_58780 | 40-99 (1.5e-08) | - | 85-281 (2.9e-23 |

Note: Protein IDs marked with # symbol are vertebrate olfactory receptors (7tm_4) that had two significant Pfam HMM profile hits corresponding to 7TM_GPCR_Srsx domain (PF10320) and 7tm_4 domain (PF13853), within their transmembrane spanning regions. Nv_205247 and Ta_58780 are sequence fragments that show similarity to 7TM_GPCR_Srsx domain.
