# Supplementary material for: Insights into the Origin of Nematode Chemosensory GPCRs: Putative Orthologs of the Srw Family Are Found across Several Phyla of Protostomes
Source: PLoS One. 2014 Mar 24;9(3):e93048. doi: 10.1371/journal.pone.0093048 (PMC3963977; doi:10.1371/journal.pone.0093048)
Supplement: Dataset S1 — List of all sequences identified in this study in FASTA format. (PDF) [file pone.0093048.s007.pdf]

List of receptors had the highest alignment score against the 7tm_GPCR_srw domain.

>Ag_004829 (AGAP004829-PA)
MEHQCVEERVLEWLHYIYSNDSARAIVLNAAEFKQRLLYILATRFNMDETGASYYLHAFE
YQNITEHNITEEHVNDFLRRVFQSLNFLNRTIPDHADVDEVEATNCSDYCKGAVREILLE
YKLLHGYISLVICFFGTIANILNIVVLTRKDMTKVPINRILKWLSVTDMFVMLEYIPFAF
YMYLILPDRRDYPYSWAVYLMFHMHFTQILHTISILLTVTLAIWRYIAIKHPHGSLAIYA
QSNYGQAIALCYILAPILCFPTYFVFTIRQTFVFDGDTQMILFHLDTDENTIVYRYNFWI
HSVIIKLLPCTILPVISCVLIQVLWKASKRRLKLKQGGNYSKNGASSVTPQSGGGSSGVL
PPISNGNRPPRTDRRADRTTTLLVAVLLLFLFTEFPQGILGLMSGILEKCFFRRCYGLFG
EVMDLLALINAAIGFVLYGLMSKQFRTSFKSVFFKTPTHRAEITRMTGITTTCV

>Ag_005229 (AGAP005229-PA)
MSSTELGEFVINVTDGPHSLAGDEDGANISTATTAHLLYCGKALDDFHTSYAKAHGIVCL
LVCIFGSIANTLNIVVLTRREMRSPTNAILTGLAIADLLVMLDYMPYAINSIPYLRLSRE
ERLTYGWAWYIMFHSIFAQICHTISIWLTVTLAIWRYIAVAYPQRNRQWCDMSKTLAAIF
SSYVVCPFLAVPIYLSFSIQSNVELLGCDGNTLTPDAIGNVSQGRNVTLYRLGTSQLVRD
NPALLNVNFWIYSVVFKLIPCIALTILSLRLIGALLEAKQRRSQLTGTATGLKQIVDGRV
VDAKAGKQTDKEKQTDRTTRMLLAVLLLFLITEFPQGILGLLSAVLKKDFFFNCYLKLGD
VMDVLALVNSAINFILYCSMSRQFRSTFNDLFRPRILDRWMAVPQGDDEQAGRGQHQDAA
TTQITQI

>Ap_003290 (ACYPI003290-PA)
MNEENYTAVVPTYLTGLFDFNGTVEGGNETDGGGYLNVTTEMPVQYARPMYGFVMPFLLL
VTIVANTLIVVVLSKRHMRTPTNVVLMSMALSDMFTLLFPAPWLFYMYTLGNHYKPLSPV
ESCYAWYAMNEVIPTLFHTASIWLTLALAVQRYIYVCHAPVARTWCTMPRVLKCVAWISV
MASLHQSTRFVDRTYEPIKISWRGQDSVVVCRMKHAYWVEHWVTLDVYFTLYYAFRVIFV
HTGPCISLVVLNLLLFRAMRDAQLKRDKLFKENRKNECKRLRDSNCTTLMLIVVVTVFLM
TEIPLAVVTVLHIISSSIKEILDYSVANLLVLFTNFFIIVSYPINFAIYCGMSRQFRETF
KELFIRGSVQINRKHGAGGSSRYSLVNGPRTCTNESLL

>Ap_002393 (ACYPI002393-PA)
MERGNGTSGAGGGAAHVSPYCGDLLVDLHDVYVHYHGYASLLVCAFGSVANVLNIAVLTR
KEMVSPTNAILTGLAVADLLVMVEYVPFAYHMYLRPANYPRADRFSYNWSLFVLLHSDFS
QAFHTISIWLTVTLAVWRYVAVVHPQINRIWCRMETTLSAIALGYLVCPIICIPSYLSFN
LFSRVETIGTSGGGGEVGGVGKDSGGPLRNVTLYYVNVSDLATSTYLADINFWVYSVVIK
IIPCVALTVLSLRLICALLEAKRRRAKLTGSGRKSADKERQTDRTTRMLLAVLLLFLITE
FPQGILGLLTLLLGKRFFQDCYQNMGEVMDMLALVNSAINFILYCVMSRQFRNTFSLLFL
PSWISKAESQALSHGNPTTTQVTQV

>Ap_41803 (ACYPI41803-PA)
MMCTDMSSNRARYNYTWLAHLRSLNLTQEDLQYLINITSFDDLDDDGGDEHHHHPMDNGC
YNCGGAVKSYSLLFRAAHGYVSLFLCVFGALANALNIAVLTRKDLAGSPINRILCGLALA
DLALMVEYTPFACYMYLTTAKKEEFSHVGAVYVLLHTYVSQVLHTTSIALTLVLAMWRYV
VVKLPNSMHAICSDRRCSIAIKLSYLLPFIICSPTFLVFEILETKVVENGTVATLYHLGL
STIARVNHELLYMIHLWTYAVIIKLLPCLILTVVTISLINALSEASERKAKRLPTQQQMA
RIRNMKLKKRMDRTSRIMIAVLLLFLATEFPQGILGLLSGILGRGFFQTCYSLFGELMDM
LALLNASLNFVFYCCMSKQFRVAFGQLFKTQPSIMFKPNNILETFV

>Am_18473 (GB18473-PA)
MPRHVHYEDTRRLNATLLHLSHLIRHISTMNRLNTSIWNSLNITDEDIDYVNKFMDNLSI
SCNENCEQQIDCYCNGTVRDLAIEYKNYHGYVALMVCLFGTLANMLNIVVLTRKDMVATP
INRILTGLATADMLVMLEYIPFAIYMYIVLPKRQIFPYGWAVFVLFHMHFTQLLHTISIA
ITLTLAVWRYIAIRFPQYNTWCTAARCKMALWSSFLAPFIACAPSYLVFGIRKQTVNENG
ILEIAYIVDADYSQHKDFFYQLNFWILGVVVKLLPCVILTVISCWLIKALYRAKGRKQAL
KSYNQCKNISVMGNGLIPKRPSKSERRADRTTKMLVAVLLLFLVTEIPQGILGLLSGVLG

DCFFRNCYHNFGEVMDILALLNGAINFILYCSMSRQFRTTFGQLFKPRIMKKWQPATQQT
DVQSTYV

>Am_11463 (GB11463-PA)
MTDFNTSSSYYVCDLSSFYSYYSHLHGWISLFVCIFGSIANVLNILVLTRREMRSPTNII
LMGLAVADLLVMIDYIPYAFHFYLYRRSRRDTFTYGWTIFVLFHSNFSQVCHTISICLTL
ILAVWRYVAVARPQQNREWCSYRRTIFAILIAYIFCPILCIPLYITTEVRKQVELLDSNG
MTVHNRNKSLIVENTTNTTLYFVRLTETAENHDILKELNFWIYSVVIKLFPCLVLTIVSL
KLLQVLLEAKRRRRKLTNIQEQNFEKKKSCRRADKERQTDRTTMMLLAVLLLFLLTELPQ
GILGLFSVLLGPRFFKACYLMLGDVIDMLTLVNSAINFILYCTMSRQFRKTFNELFCKHW
NISKNSGKQICIQNNGNTGTNHTQVTQV

>Dm_Q7JVS8 (tr|Q7JVS8|Q7JVS8_DROME) (FBgn0033579)
MMQETGNQMGQTHMHQRVPFNDTVLKDYHLTSTDIEKFVKLWQEYQMKNMTPQVDECQGY
CQGEIYNWLRAYNSIHGYVSLMICIFGTIANILNIMVLTRKEMAKTPINNILKWLAVADM
FVMLEYIPYTSYQYIYMGPGEKDLSYTWAVCLLVHMHFTQILHTISIGLTVTLAVWRYVA
IRHPNGGCANFLLAHSREAILLPFILSPILCLPTYFVFQVRETYDVDKVNSEAMYHVYFD
KDSVLYRFNFWIHSVLIKLLPCGILIVISAVLMHVLCEASRRRLKLRDYNNPAKYAIQLN
LNETKSKKPPRCDRRNDRTTLLLVAVLVLFLITEFPQGLLGLLSGVMEKCFFAHCYPPFG
ELMDLLALINAAVGFVLYGLMSKQFRTTFRSLFMKRHFGSTEMTRLTRVTTTCV

>Dw_B4MK79 (tr|B4MK79|B4MK79_DROWI) (FBgn0222655)
MKIKNKSLAEISECEGYCQGEIYKWLRTYNGMHGYVSLLICIFGTIANILNIMVLTRKEM
AKAPINNILKWLAVADMFVMLEYIPYTSYQYIYMKGDKDLSYGWAVYLLFHMHFTQILHT
ISIGLTVTLAIWRYVAIRHPNGSCANFLLAHSREAILFPFLISPILCLPTYFVFKVRETH
ELATDEAMFHVYFDNNSILYRFNFWIHSVIIKLLPCCILTIISLVLMHVLCEASRRRLKL
KDYDTTAKYAIQFNLNDQTKSKRPPRCDRRNDRTTLLLVAVLILFLVTEFPQGLLGLLSG
ILKKCFFEHCYPPFGEIVDLLALINAAVGFVLYGFMSKQFRTTFRSLFFKRHFGSSEMTR
LTRVTTTCV

>Ph_10750 (PHUM110750-PA)
MGFAQPLYGYIVPFLLVITIIANTLIVVVLSKRHMRTPTNFVLMVMALSDMFTLLFPAPW
LFYMYTFGNHYKPLWPVAACYLWNAMKEDIPALFHTASIWLTLALAVQRYIYVCHAPVAR
TWCTMPRVIKCVAWIYVFATLHQSSRFFDRTYTSINITWNGVPDVAVCKVNLATWVTDIV
TEDVYFTVYYCFRVVFVHSLPCVSLVVLNVLLFRAMRAAQLKRKELFKENRKCESKKIRD
SNCTTLMLIVVVTVFLAVEIPLAVITVLHIISSGITEILDYNIANVLILFSNFFIIVSYP
INFAIYCGMSRQFRETFKELFIRGAITTRRNDGSSRYSLVNGPRTCTNETVL

>Ph_309270 (PHUM309270-PA)
MEYSMTTTTTTTTTLPFFNPNLSYESLRKFIQSMNLTEEDLYRFFKMHFPNDTIRGLILA
PREICLYDPDKYCDGTLKAFANGYKDVHGYLSLMVCLFGTIANLINVAVLTRREMATNPI
NRILTGLAVADVLVMLEYIPFSVYMYLIFPGQRNFPYSWAVFILFHVHFSQIIHTISICL
TLALAVWRYIAIRYPHKSITWCTGPRCRVAIGTSFVLPLILCIPSYFVFYIKTTRITENS
AEIYLYHLDLSDVAKENDQLLCNVTFWTYGVLIKLLPCVILTVISIRLIKALYEINKRNK
LLKTANSTHHNENSVERRVILAKERTDRTTRMLVAILILFLLTELPQGILGLLSGILGTC
FFRNCYNLFGEIMDILALVNSAINFILYCFMSRQFRQTFEKVFKINLFTENVPRIGPPTD
MQSSYV

>Ph_54050 (PHUM054050-PA)
MWGWVHGYLSLIICLFGSVANILNIVVLTRKEMKTHTNLILTGLAIADLLVMVDYIPFSY
YFYVKSKKYTYGLAVFILFHANFSQVCHTISIWLTVILAVWRNIAIAHPQINRAWCTNRS
TIVAIVSTYVICPLICIPLYLVYTLKTSKYKNETIYHVEANNSPSMRDVNYWIYGFVIKL
LPCILLTFLSFRMVLFLIKNEKRQKLLISSKALINTETRKSKNQTYREKQSHRATKMLLV
VLILFLITESPQGILGLLSYSLGDHFYNSCYHPLGDVLDFLALFNSSTNFMIYCTMSRQF
RVTFNLLFKPKVLNKWMVTSQVNNVTKDATQLTQV

>Ph_423330 (PHUM423330-PA
MDWLTESNSGEEYVDNCTYSLSSLDFLPKYNSNDIPNLEIQWPCEVRTYWVQISPFVSTV
KFLLTYVTLVIIIIGITMNTLSFLILNSSPICHTSLSVFLRALAVSDNGALLFNFATGVG
RSHVPIFAKLYLESVWLCGANKILIDVFLFYSTWLVVGLTVKRLLVIGCPMKFSFKCSVK
KAKRVVYSIGLTSFSIACLKLKYSGYEQDSTFGFKPCKFQSEGVDNKVVYVYIALSTWLP
CLLIFIGNILLIRQVRMTNKSRFKLSVKRFNDTHRMLGLLITVSITTLVLMLPLGIVETF
ELYWDVVLIKKPTAQDPGRQQYIKCAARNYFKNCFQTPCCCRTFVSTSITRIRYQLSKLT
RSNATTSTDIEMESQNNNRITHNSPEMGGKDEEKTS

>Dp_P2838 (DappuP2838)
YCGDGLEAFRKSYLIMHGYISLVVCLFGMLANTLNIIVLTRMDQRISPTNAILTGLALAD
NLVMIEYIPFTMHMYILHGRPVYERFSYPWTVYVLIHAHVTQVFHTISIWLTLLLAVWRY
LSVAHPLRSRDWCTLDRALLAILLAYVVSPLVCIPIYLTYTIQETPYQVPPPPPSNRSAA
AQMLLANATGQQHWLLDEQQKNHTLYVLGTSELARANDSLLENINFWTYSVVVKLIPCVA
LTFFSFRLISALVSAKERRQNLKSNNTHSSKKNALASSAMGGGDPEHSVSAVERHNTGSE
KHDRTTRMLLAVLVLFLVTEFPQGILALLSGILGDAFFTNCYRHLGELTDILALINGAIN
FLLYCVMSRQFRQTFSRIFRPRLLDKW

>Dp_P2282 (DappuP2282)
YLNITADLDISYALPLYGYIMPFLVVITIIANTLIVLVLSKKHMRTPTNLVLMAMALSDM
FTLLFPAPWLLYLYTFGNHHRPLRPLSACYAYNFMNEVIPALFHTASIWLTLALAIQRYI
YVCHPPLARTWCTMERVKKAICWIYVLALVHQFPRLFDQVYLPVEIRWDGEGVVTTCCKY
EADWVTDIFSQVVYYNLYFWFRVVCVHLGPCASLVLLNVLLFGALRRAQQKRDKLFKENR
KASECRKLRDSNCTTLMLIVVVSCFLVVEIPLAVLTVLHIFDASLELRVLDYSVINVLIL
FTNFFISLSYPINFAIYCGMSRQFRETFQGLF

>Dp_P22307 (DappuP22307)
HGILSLIICLAGCLANIVNIVVLSRRELRRNAINRILCSLAVADFLLMLEYIPFACHMYL
FPGRSLQTRYSYPWAVFVLFHAHFTLVAHTVSIWLTLSLAFWRYEVLKNGRHQRRKNGRR
CHQRCTRTIAMAYIGSVLLCIPSFITFGIQSEPIKNTNFTINHTQLPTASSWSSSSTPLQ
LPEAVIYKAHLWAYSVVMKLGPCLVLTVLTCWLVRRLWEAERHHQSLTANGQIRQTELVI
PARNRRKLSHQTDRTTRMLVAVLVLFLVTEIPQGVMALLSGVLGRQFFIKCYSTGMGEIL
DLLALLNSAINFLLYCSMSRQFR

>Lg_94839 (jgi|Lotgi1|94839|gw1.71.238.1)
FHQWFSNFHGYASIIVCIFGISTNIFNISVLTRKDMRTPTNILLTWLAVADILTMVPYIPFAIHFYCP
NTSPFETPEKYTYNWILYMIFVVNSAATTHTISNWLGVSLSVFRFMQMRSTSRGVLAKQRRLKHVKVI
TVVVYIFSIIVLIPNYLTNKIQSVQGPRNTTIYGLKDMTPKDPSTDKMALINTLLYAVVAKIVPCLLM
SIFSVSLVYTIHYKNRHRMRRLVAAGKKSRAISKQTTTTRMLLVVIVLFLITELPQGILILVTAAIPQ
FHNNVYNLLGDLMDFIALLNNAINFVLYCSMSQQFRSRFIEMY

>Lg_105511 (jgi|Lotgi1|105511|e_gw1.4.616.1)
MSFNSSTGVFEFYRVPELESFSVWYSGIHGYLSVIVCGFGIPMNLINVTVLTQKHMRTPINCILTFLS
LSDMLTMISYVPFALNFYILHSPWELNATKNSLGWMRFMLFHINFSATTHTVSIWLGVTLAILRYKHL
YSPAKGNLTRIRRLIRARISVVVVYVLSTVGLTPNYITNKLHPSPFNNETVYIMESLDLGTNDVRPVV
LLNLWLYSIMAKLLPCILMVIYGGLLLYTLKSKLKFQKRKLSCTSVYAQRPLDNSRTTRMLLIVIILF
LVTELPQGILIIILSVTSRGFFHFIYMPLGDLMDMMALINNGVNFILYCSMSRDFRLTLMDLLKIRIAK
HHFTTVACDSERSQTALIVRVS*

>Lg_124239 (jgi|Lotgi1|124239|e_gw1.46.322.1)
MTERPQVDPNLDRFSTWYSTYHGYVSLCVCMSGILLNIFNVTVLTRKHMHTPVNHILTGLAISDIITM
LSYVPFAVHFYCMHPTEAGTPEKNSIYWMTFLLFHINLTTVTHTISIWLCVSLSIIRFLHIRSPTRAR
VVRLARMRQSKYLVVLVYFTSVIVMIPNYLTNELKPAHVSTNETNSTIFVLENLRLGRNDTTPLVLIN
VWLYAIVAKLTPCLLITVFGCMLLYQMQVQVKRRRSIIAKFSGTSGIKLKEHSRTTKMLISVIVLFIV
TEFPQGILIVLSACKANFFETVYLPLGDIMDIVALVNNAVNFVLYCTMSTKFRETFLRQYCTMPKFPK
TENGFTLREKLDSYSNNNQV*

>Lg_153743 (jgi|Lotgi1|153743|fgenesh2_pg.C_sca_4000202)
MGLMNNTIEDNYISDDNRTVQQGGWFSKPDPNLNAFAYWYWNIHGYLSIFVCIFGIVTNFINIQILTR
KPMRTSVNVVLTAVAISDIALMASYIPYAIHFYIACGLDPTPDRYSYEWTTFMAVHANVTLTTHAISI
WLAVFMAIQRYVYLQTKIFMTARNTVIACLVICFASGLVMLPNYLITGVYEVKTSATNGSTGMYRLNE
FALGSKRPSSVVLLAFWLYAIVGKLLPCVLISVFMGFLLKRLHESTERRRRLFSTNASSSSSSKKKRA
GHKSTTTMLLVIIILYIVTELPQSVLVILSVIFDGFFENVYYLLADILDMAALINNAINFVLYCIMSQ
QFRGHLMEMWVFKKCKHSRCFLPTNVKNVQSNIDDMATTMVTEKTVIRAETIKLLKT*

>Lg_94475 (jgi|Lotgi1|94475|gw1.46.313.1)
LQSFGECYWYVHGYYSLIVCLFGVITNFINIAVLTKKSMRTPINCILTGIAIADLVTMLSYIPFAIHF
YIANDIKATPSKYSFSWSVFMAIHVNLTLTTHTVSIWLGVIMAIMRWVFVRNATPNNRKNSENRSAII
IIVMTYIGSAILIIPNCFVTGIHEEFSEEHNGSMYRLNSLSLTNETSSMAILNFWVYPVFGKIIPCVL
ISIFGGFLLHTLRESDRRGSRLKGDNHKCHAHRRTTLMLLAVIVMYIISEVPQSILVVLCACVQGFFV
DVYLPLSDLIDAIALINSAINFVMYCTMSQQFR

>Lg_160192 (jgi|Lotgi1|160192|fgenesh2_pg.C_sca_24000006)
MVQFAITYGNIHGYFSIVVCIFGIISNILNIVVLTRKHMSSPTNYILTALAIADMLTMSTYPIMACYL
YIISRPDCDGTNHSKEWMYFILFHNLFIVTCHNMAMWLTVSLAVFRYIFVCQHAMAMVMCSLARARVT
VMAIVGATILICLPNYLMYTVIDYGKSNNMTSCFWVVSSEIAVRYPAYEMFVRWLFGVVIKILPCILL
TVLSTLIIIAMHEAKKRRSRLLGMVNRASDHDNASSEHNRTTMMLVLVVLTFVITELPQGILALISAV
NTDFFSRVYVNLGDLMDLLVLINSAVNFILYCIMSQQFRNTFKSLFVGKYFRPFFNKNPSQNGTSYTM
VKTETTQV

>Lg_114444 (jgi|Lotgi1|114444|e_gw1.19.420.1)
MAKLDSFSVKYSVFHGYISAVICAFGIMANIANIIVLTRKNMISPTNLILLWLAVADLFTMLSYFPFS
LCFYVFRDDQLRMFHTRSAGVIYFLMFHASFSIVCHTVAIWLTIALAIFRYIYICRPTKGAIYCNKRR
AKLTILAVYILTMILCVPNYATNRIEYKIRTTNSSEIINSTVRYNYINETIYNPTVVTGEAADIIKQT
NSLIQAVLFKLLPCAMLTTLTILLIHAMHKAYRKRMRLKSQGRKAESDKHGEHNRTTGMLLAVVVLFT
LTELPQGILTLMNIFIQCFQWYVYDKLGDLIDTMALMNNSINFVLYCSMSKQFRDTFTEIFCKCFMKH
QSGFFKMKPVKSELNGHVHRDSNCNNSHV

>Lg_95120 (jgi|Lotgi1|95120|gw1.19.432.1)
NHTVIELALFASEYSQVHGYISAVVCTFGIIANIVNIIVLTRKNMISSTNTILMWLAVADLFTMLSYL
PFIMRFYIFREEDLDRFNTRDYAWICFLMFHASFGIVCHTVAIWLTIALAIFRYIYICMPTKGAIYCN
VRRARIAIFLVYLLTVVICIPNYIINAISTDSLPPKQSLGPNGTNVSDKSDIFYYPSLRQSTTADVFI
YQSNVYIQSILVKLIPCFLLTTLTILLIHAMHKAYRKRMRLKSQGRKAESDKHGEHNRTTGMLLAIVV
LFMLTELPQGILTLMTLFEPCLQFYVYNNLGDLVDVMALINNSINFVLYCTMSKQFRDTFVKVFCK

>Lg_105773 (jgi|Lotgi1|105773|e_gw1.4.628.1)
MDNITDIPNATNLELWANWYRHVHGYAAPIVCIFGVVANILNIVVLTRKNMISPTNVILTGLAVSDGL
TMAVYFIYAILSHHIYVNRPTPLTHAQFVMFYAIFSVIAHSISIWLTVALALFRYIFIRCPRRGGKLC
SIDRAKLTVVIVSFVTTVVCIPNAVSYQIVDQGNGSSWYVDVKKTTATEIILTRFNFWIQAMLVKLIP
CFLLTILSILLVKTMKDAEKRRKKLLSKPMKGSDDPRRPSKTNRTTRMLLAVVILFLVTEIPQGILNL
ISGIEEDFFDNVYTPLGDLMDILALINNGINFILYCTMSKQFRDTFIKLFLKDLVMDNNRGTYHCTQV
TRDTEL

>Lg_94766 (jgi|Lotgi1|94766|gw1.3.630.1)
PENYSVIINGYISPILVFLTIINNSMVCIVLLKRNMRSPTNTLLVGMAISDMLTGVFPLPIFLEFFAT
DRLKEFTPYSWCNAYKYLAELLPTIFHTASIWLTMALAIQRYIYVCHSFKARTWCTIPNAIKGTIAIY
VVAILSQCTRFFDSYFVPVVLESAVYENRTVIGCIEEHEDWAMANILYMSIYYWFRVLFIHLIPCIFL
VVMNALLIYAMRVAQVRRMQLLRQNKKSESKKLKDSNCTTLMLVAVVGLFLLVEFPLGIIMILLIVSN
TFEVHIISKETFQILSIFSNLFILLSYPFNFFIYCGMSKQFRETFKRLFVG

>Lg_139911 (jgi|Lotgi1|139911|e_gw1.141.105.1)
MADLTGELTSVIRSIDPYVHLCHNLSTAIYSKRKSMTVLDFAEMFPLDVAVPVFGYASPIVILVTLIL
NFCVIKVLSSSKFRSPTNVMLVTIAVLDILTGCLQLPWYIHTYSLHGYRQLPDQFWCHSYYYLFRVTP

IIIHTASLWCTALLAFQRYMGVSQLRRIGCVFQYKGLIGCFVTIGAGSFIIHSFALTLVKLDSVQVIS
NDIIKADGSLAYTNTCAISYRNADFRMYYSQYYEWTRAFLAQILPCVILGIFNLLLIRITIKGYRYRR
RLVRGNKAAQNRELRTTLRMSIMLILITGSTLLAETFVAILLIIQTSQEHTEIVLADPTNVSKSMVIS
NFLILFTFPLNFLFYFLMSSRFRRHFVNCYIQRKHRRIVPLLGQSQNKNGNDEDDDDDEDEVDAAPQI
SPVQMNNFQKEENSRYNYSSQTM

>Lg_160329 (jgi|Lotgi1|160329|fgenesh2_pg.C_sca_24000143)
MNVSNNSSGIDEQETTLISQEVFFEFNTALAQSWIPLAVIGIILNAVNIIVFADARIRNATSVYLAAI
SAWQIVLLVSGMISKIHQFIYGTIGQRSTKFFYFAAIYINNFVVAAVQRIIFYLVFLVALERMLAIQN
PMKKHYFRWLKAPGKCILLVFCITFTFHIYSPLKYTVSDTVGDDGRTKYVLERTNHDLDVLINFGFAA
KIIFVYCPLLGIMCCNIILTISLNRKRKIRQQLQEVFVGREARDRQTNVAILVSTFVFFLFNLPSTTN
AFIGDVLPNYSYFSSNRYTLMFFQGVGSLSALVSCSSDFFTYIIISQTYRNIFMSKFLPCLKPRIEVP
THRYVSGNQNSSFTQSSSMSSLPERVSSN

>Lg_171916 (jgi|Lotgi1|171916|fgenesh2_pg.C_sca_122000034)
MDFSSEQNSIMFLLLLIPIYMVAAQETSEILDATDLDGGVMNITDPVTDSGQQITNGNATMNWENLET
EEIISAMGNEEFHNRTNDSVVNTTSKMPAIGGFISKEIYDQAIQITQNIWLPVCCIGLVTNILNIVVF
LDREMRKSATSLYLVGISLLQITYFIVTSIRRIPPLIYGEYAYTKTKFYLLSVIYLSNYFGAALTRTV
YCLVMLVAFERMMAILFPMKLAKTRLVKSPIICMICLIIVSMTFHVYLALSFYLSETIGEDGIVKYGM
IRTDFDPVVINRFSMAAKIIFVYIPIIGLFVFNVVLCVALKRSFKTRKSLQESRSDRNQEKRERQTTI
AILVTTCIFFVLSLPMPINSIVVNLVPEYNYFNENRYTFLFVQEVGSLLSIVSYCVDFVTYLIISSRY
RLTLMKKCLPCRYHLKSKIHSTNTQVSGKSLSTGSG

>Lg_160694 (jgi|Lotgi1|160694|fgenesh2_pg.C_sca_25000299)
MYDTQNITQSAAPAFQFYPEGYFVLTEKQLDDARFAFYVVLNPLTALFGIGGNISSFLILIRKTMRTT
QNLILLALSITDAMHLLATFCHSFSHIMTYFYKQEWNNLINDAFPYFGAYLYHVWGRISAMLTVCITL
ERLIAVTMPLKVKMIWTKKVTLVCIFLSYFIPATIMVTQIFEYEAAHVYFPNSTDYKVTLVLTDLGKD
RILFTNLYNANMTILRYMPIFIVSLANLIIIAGLWHHARQRKHLKTGSESMISSKELKITRTLIMVSV
VFFISSAPGTTVFLIQNLDPEYNFPFFRNNGYTMALIVSLWLEVLNSSINFVIYVATNSKFRDMYCEM
FPCCGKVKKQNVYVDSSKKTTSISVLSDQETNEVTPSS

>Lg_158519 (jgi|Lotgi1|158519|fgenesh2_pg.C_sca_17000170)
MFSLARHILSYTILICTIYNDVVTTQLLFHKYKILYSRKVDNAKTTVSYEGVRDSGANTVRIRNIEVE
MPFKQETSSFAEISEGGYGEDEIIQRPKRDTKKTNTTERVISAVDSNSLKFVDGDEQDINQTIVNSNI
SLRINSTDPETYTPAAKSGMVSVINTGLISKTTYDKLERTVRYMTYVTQIIAVVLVCFNIIVFSRHTM
RSPTSSYLIAVNISQIFTIVSGSFLTIAGFIFGDKAFFSYNYLFIGLYISNYFTVATRRCTFVLTCLV
AAERFLAVAFPLKSKHFKLVRKPILFIVTIAVLSFIFHIFSVLKYDIIPFQVGISNETFWKFQFTKRF
LENKANMESWSIASKIIFVYIQLIGCLILNIGIVIALKRHRMNRLRLNSSEDVSKVAQEKQTTVTILV
STFVFVLLALPVNTSSVVANLNRINYGINTKNHYLFYFLMHIGGWFELLSDCTNFMFYIALSSRFRKT
FAQVICRCFSTPLERTESIDYNSTRTCQYGLSVVSQSSH

>Lg_158529 (jgi|Lotgi1|158529|fgenesh2_pg.C_sca_17000180)
MVDVRLSKGLFLMVLHSIIVAALQNTNNNSASGISNCSSPESDVWCCKCITKKLNGLGNETNVIKGIE
FFPVEDILDGLVLYPDYSDLKDADNNTILNQSNITNSNKTTVESQVGISHATYTRLNIVMGHIWFPLC
AVGLVANIFNVVVFLDKKMRNATSIYLASISIFQSTFMIVTSIRRVPQLIYGEIEQRTTKFYYVNVIY
LSSFGGTTLIRVVYCMVMLVAFERMLAILFPLKRARSRLVKSPVLSIVTLLFVSLGFHTFTTLNFRIS
TYQSETGEAIYALKRTEYDQVVLNNFSVASKVLFVYIPLTGIFIFNMILCVSLRRSSQSRKDLHEVNE
NKGRERQTTIAISVSTFMFLMLNIPMSTNSIFGKLLSGYGYFSPLRYTFLFVQEMGNILSLISYCVDF
VTYTCMSNKYRETLTRKCCPCRYRQRLENRSLYGVPSVHSATHHSVV

>5a (AASC01117380)
MSFSNLWVNNLTDSRDDSVPITTSPSPSHYDIMSDEQWYIVQVVNGVVLCGLVSLFGVVSNIINIVVF
AKQGFQDSMNISLMGLAVSDLCSLVTTIWLSICVNPLFYLSELPFDPRDIMYLTGSMPRYLFVKIATF
ITAFITFERCLCIAAPLKVKMVITPVRTKMIIISIYITMSILLIPFCLGHRLEWVFDFTLNATVLKTT
FTAEREMLEAITYLTQGVFTTTLSFVFVICCTIVLVVKLNSKTKWRQATAAKGDRAAEGVGVKDQKVV

KMVTFIAVIFIVSAVPPTLLFFYMLFDTNFRIDGAYPNLYSTIWCLSFLTETVNSSVNIFVYLKMSSK
YRAVFIKTFLNKQEK

>12a (AASC01093420)
GLAVADLCSLLSMIWLSICWNPLFYNSELPFDPRDILNLTGAVPHVLLVKIATFITAFITFERCLCIA
VPLKVKTIITPRRTKTIIISIYIAMAVLMIPFCLGNRLEWVYDLTLNATVLKATYTAERDILEAITLL
IQDVIATTFSFVFVVCCTIVLVVKLKSKTKWRQATAAKSDRAAGGVGVKDQKVMKMVTFIAVIYIICA
VPSTLFFLYIVIDPSFHHDGSYRNLFLVLCFLSFLTETINSSVNIFVYLKMSSKYRAVFMKTFWN

>15a (AASC01194155)
MSHTSLPPRMSISHLWGNNSTYSHDQKMSLPMTTPSPSGSQYANIVSDEQLYIIQLVNGVVICGLLSL
FGVASNIINIVVFAKQGFQDSMNISLMGLAVSDLFSLLTTIWLSICANPLFYNSELPFDPRDLTDLTG
GMPHILFVKIATFITAFITFERCLCIAVPLKVKTIITPRRTKTIIISIYIAMAVLVIPYYFGNRLEWI
FDFNKNATVLKTTYTAERGMFEAITFLTQGVFTTTFSFVFVICCTIVLIVKLNSKTKWRRATAAKSDR
AAEGVGVKDQKVVKMVTFIAVIFIVCSVPPTLMFLYMVIDPSFRIDGVYGNLYFVIWCSSFLTETVNS
SVNIFVYLKMSSKYRIVFMKTFLNKGEK

>18a (AASC01187084)
GLAVSDLCSLLTMIWISICENPLFYRSELNFDPKDILYLTGATPHILFVKIASFITAFITFERCLCIA
VPLKVKTIITPGRTKTIIISIYIAMSLLMIPFYLGNRLEWVFDFTVNATVLKTTFAADREMLEAITFL
TLGVFSTTFSFVFVICCTIVLIVKLKSKTKWRQATAAKSDRAAEGVGVKDQKVVKMVTFIAAIFIVCA
VPPTLLFLYMVIDPSFRIDGVYRNIYLVVWTSSFLTETVNSSVNIFVYLKMSSKYRAVFMKTFWNKEE
N

>27a (AASC01105652)
MSHTSLPPRMSMSHLWGNNSTNSCDYNMGLSMTTPSPSGSYYANIVSDEQLYAIKMVNVVVISGLLSL
FGVASNIINIVVFAKQGFQDSMNISLMGLAVSDLCSLLTMTWISICENPLFYRSELNFDPKDILYLTG
AIPHVLFVKIATFITAFITFERCLCIAVPLKVKTIITPGRTKTIIISIYVAMSVLMIPFYLGNRLEWV
FDFPTNVTVLKTTFTAEREMLEAITFLTQGVFASTFSFVFVICCTIVLIVKLKSKTKWRQATAAKSDR
AAEGVGVKDQKVVKMVTLIAVIFIVCSLPPTLLFLYMVIDLDFRINGVYRNLYLVVWSSSYLTETINS
SVNIFVYLKMSSKYRAVFMKTFWTHRKK

>1b (AASC01147398)
MASGNESYGTPLGPPQSRMVDDHVLAIIIICFFVIILGTISFFGIVANVINLIVFIRQGFKDTVNISL
FSLAISDIGALIPLLWMSIGLNPYFFADPPFDPQEVLYLTSGWPHICFARISGWITAFITLERCLCIA
MPLKVKTMITVKRTAIALVSIYVAMIGALLPVFYALRLEPIFSPLKNETKFRTVYIPNGFAIESAVFL
INAFSQFVSFIVVIICTIILVQALISKSKWRKSTSSFGDHDSFSNRDKKVVKLVLFIAIIFIVCFLPS
AINFLASSLEPEFNTYGKYGDLFVLMWSFSANLSATNSAVNIFVYYKMSSKYKQILDKMLLRSTKG

>2b (AASC01177837)
MASGNETYGTSFGPRPSRMVNDLVLTIFVIGFYVIILGAICLSGIVANVINLIVFIRQGFKDTVNIFL
FSLAISDIGLLIASLWLSIGFNPYFENACLPFDAKEVAYLTAGWPGICFSRISGWITAFIMLERCLCI
AVPLKVKTMITVKRTVIALVSIYVAMIGALLPVFYAIRLEPRYSPLKNETKIRIVYIANGFAIESAVF
LINAFSQLISFIMVIICSIILVQNLISKSKWRKSTSSSGDHDSFSNRDKKVVKLVLFIAIIFIVCLSP
SVINFIAQSFEPEYTASGKYGDMFLLIWSFSSCLKATNSAVNIIVYYNMSSKYKQTLDKMLLRCKKG

>3b (AASC01132996)
MASGNETHGTSFGPRPSRMVNDLVLAIFVIGFYVIILGVISFFGIVANVINLIVFIRQGFKDTVNISL
FSLAIADIGALIASFWLSIGFNPFFANACLPFDPKDVLYLTAGWPGTCFSRISGWITASIMLERCLCI
AVPLKVKTMITVKRTVIVLVSIYVAMIGALLLVFYALRLEPRYSPLKNETKIRIVYITNGFAIESAVF
LINAFSQLITFSIVIICSIILVQNLISKSKWRKSTSSSGDHDSFSNRDKKIVKLVLFIAIIFIVCLSP
SVINFIAQSLEPEYTASGKYGDLYLLIWSFSASLSTTNSAVNIFVYYNMSSKYKQILDKMLPRCTKG

>7b (AASC01137539)
MDAGNRSKPESPEPQRTGLVNDEVLDVFVVLFFVVASGTISFFGVLFNMVNVIVFYKQGFKDTVNITL
FGLAISDMGSLVTLLWMSICFNPWFANADLPFDHQDIQYLTAGWPHVCFARITSWITAFVTFERCLCI

ALPLKVKTILTPRRTVIVIVSIYFVMIASVSPVYYSIRLGPKYFPQRNETKIGLVYRPSGPFIENVSF
SINVFAQLASFFSVIVCTAILVQNLLLKSKWRQATSSSAKQDTFTNRDKKVVKMILFISSIFIACFLP
SAVNLIAMIISAEYSIVGRYQNMFLVTWAIFNSLEANNSTVNIFVYYNMSSKYREILDKMVNKKQIKK

>9b (AASC01123121)
MDSGNNTSQTKVSESTQAGLVNKDVMNLFILVFFVHASGTISFFGLIFNIINIIVFYKQGFKDTVNIT
LFGLAISDMGGSIIHVWMSICFNPYLFADIDLPYDPSDISYVTGGWPHVCFTRITSWITVFATLERCL
CIALPLKVKAIITPKRTVLVIVGIFVSMIASLVPACYSIRLGPKYFPERNVTKIGLIFIPGGIYIENI
TVSINAFSQLSAFIAVASCTTILVHNLIQKSKWRQSTSSSTKQDSFSNRDKKVVKMILLISIIFIICL
LPGSVNLVTTFINHEYNMAGRYRNLFISLWGIWVSLEALNSSVNIFVYYNMSSKYKNILNTMLHINN

>10b (AASC01191494)
MGSGNHTSQTEVLESTQTGLVNKDVMNLFILVFFVHATGTISFFGLIFNIINIIVFYKQGFKDTINIT
LFGLAISDMGGSIIHVWMSICFNPFLFADIDLPYDPQDISYITGGWPHVCFTRITSWITVFATLERCL
CIALPLKVKTIITPKRTVLVIVGIFLSMITSLAPAFYSVRLGPKYFPERNVTKIGLIFRPGGIYIENI
SVSINAFSQLSAFIAVASCTTILVHNLIQKSKWRQSTSSFAKQDSFSNRDKKVVKMILLISIIFIICL
LPGSVNLVTTFINHEYNMAGRYRNLFISLGGMWVSLEAFNSTVNIFVYYNMSSKYKDILNTMFHIKN

>11b (AASC01159697)
MGPGNESQTALSKITTRGIVNDNIRYIFVLIFFVSATGTISFFGIIFNIINIIVFLKQGLKDSVNITL
FSMAISDTGSLIALLWMSVCFNPWFINSGLPFDHIEMQYLTAGWPHGLFARITSWITAFVTFERCLCI
AVPLKVKTIITPRRTVVVVVCIYLTMMACVAPIYYAVHFGPKFFPEKNKTMIGLVYIPNGLLIQSISL
TINAFAQFGSFFAVIICTTVLVLNLRRKSKWRQSTSTSAKQESLSQRDKKVVKIVLLISTIFIACFSP
AAAAFIAMSIVPGYNIVGRYQNMFLLTWSMFKTFKATNSTINIFIYYNMSSKFKEIFDEMFHRNREID
HQDNTI

>12b (AASC01159697)
MRSGNENQTALSEITMSGIVDDGILYIFVIIFFVTATGTISFSGIVFNIINVIVFLKQGLKDSVNITL
FALAISDTGSLMGLLWMSICFNPLFINSGLPFDHIDFQYLTAGWPHVLFARITSWITVFVTFERCLCI
ALPLKVKTIITPRRTIVVVVCIYLFMMACVAPIYYAVRFGPKFFPEKNRTLIGLVYIPNGIFIQSISM
TLNAFAQFGSFFAVIICTAILVHNLIRKSKWRQSTSSSAKQESLSQRDKKVVKIVLLIATIFIACFFP
GAVAFIAMSIVPEYNIVGRYQNMWLLTWSMFKAFKALNSTVNIFIYYNMSSKYKEIFDEMFHRNRETD
HEDNI

>13b (AASC01064969)
MGTGNESHTTPSEPASGGLVSEGLRDLFIVIFFVATTGTISFSGVVFNVINMIVFLKQGLRDSVNISL
FGMATSDTGALLGLFWMSICFNPWFVSSGLPFDHIDVQHLTGGWPHMIFSRITSWITAFVSFERCLCI
AMPLKVKTIITPRRTMIVVACIYLILITCVTPVYYSVRLGPKFFPKKNKTLIGVTYIPNGSSIENISL
ALNILAQFGSFFAVSICTAILVHNLLLKSKWRQSTSNSAKQESLSQRDKKVVKLVLLISTIFIACFFP
GAVAFIVMTLFPGYNIVGRYQNIFLLTWSMFTVLQATNATVNIFIYYNMSSKYKEILDELLRRKKGND
NQSTKNY

>14b (AASC01064969)
MGPGNGSQTTLPESVSKGIVSAGLLNIFVITFYVAATGTISFSGIIFNIINIIVFLKQGLKDSVNITL
FSMAISDTGSLMGLFYVSISFNPWLVNADLPFDPLDLQHLAGGWVHILFARITSWITAFVTFERCLCI
ALPLKVKNIITPRRTIVVVVSIYLTMLAFVAPVYYSTRLGPKFFPEKNKTMIGLLYVLNGPSIEKVSL
ALNVLAQFGAFFAVIICTVILVHNLLLKSKWRQSTSSSAKQESLSQRDKKVVKIVLLISTIFIFCFFP
GAVSFIAMSTVNGFNVNGRYHNIFLLTWSMFKTLQATNSTVNIFIYYNMSSKYKEILDEMLHRKKGNI
D

>15b (AASC01189496)
MGSGNESQTALSKTTTGVLVNDRTLHIFVLIFFVSATGAISFSGIIFNIINIIVFLKQGLKDSVNITL
FSMAISDTASLIALLWMSICFNPWLINSGLSFDHKEMQYLTAGWPHGLFARITSWITAFVTFERCLCV
ALPLKVKTIITPRRTIVVVVCIYVTMIGCVAPIYYAVHFGPKFFPDKNKTMIGLVYVPNGHFIQSILL
TINSFAQFGSFFAVIICTAVLVYDLRRKSKWRQSTSSSAKQESLSQRDNKIVKIVLFISTIFIACFSP

AAAAFIAMSIVPGYNIVGRYQNMFLLTWSMFKTFKATNSTINIFIYYNMSSKYKEIFDEMFHRDRKTD
HQEI

>16b (AASC01202310)
MDSDNDTSQTEISESTQTGLVNKEVTNLFISVFYVHSTGIISFFGLIFNIINIIVFYRQGFKDTINIT
LLGLAISDTGGLITHLWISICYNPYLFADLDLLYDPTDIGYLTGGWPHVCFTRITSWITVFATLERCL
CIALPLKVKTIITPKRAVIIIVGIFVTMIASLAPVCYSIRLGPKYFPQRNVTKIGLVFIPGGIYIENI
AVSINAFSQLSAFFAVTSFTTILVQNLIQKSKWRRSTSSSAKHDSFSNRDKKVVKMILLISIIFIICL
LPGSVNLITTFINDEYNMAGRYRNLFLCSWGICGFLDALNSTINIFVYYNMSSKYKDILNTLLCIKK

>19b (AASC01182788)
MGPGNGSQTTFSESPEEGIVDPELLNIFVIMFYVTATGTISFFGMVFNIINAIVFVKQVLKDSVNITL
FAMALSDTGSLLGLFWMSICFNPWFVNSGLPFDHLDVQYITAGWPHVLFARITSWITAFVTFERCLCI
ALPLKVKTIITPKRTVVLVVCIYVATIASVAPVYNFVRLWPKYFPKQNKTTIGLVYVGNGPFIENILL
AFNVLGQFGSFFAVSICTAVLVHNLMLKSKWRQKTSTAAKKESLSQRDKKVVKIVLLISTIFIVCFFP
GAVSFIAMSVIPGFGIVGRYQNTFLLIWSIFKTLQATNSTVNIFIYYNMSSKYKEVLDNMLNRKSDKH
G

>20b (AASC01152348)
MDSGNHSHVTPSQSSEKGWVSDELLNIFVVFFLVAAAGPICFFGTIFNAINAIVFFKQGFQDTVNITL
FFLAISDLGTLVNIFGMSICYNPWFVQADLPIHYHDLSYLTTGWLSLCFARISSWITAFVTFERCLCI
ALPLKVKTIITPRNTVIVILGIYVTMIVSVAPVYYAIGLGPKYFPQLNATKIGLVYISNGVSIENVSL
SINAFSQLFSFFATITCTIILVHNLLLKSKWRQSSSSAVQQKSFSKRDAKVVKMIVFISSVFIACYLP
SAVNFIATVIFDEYGVLGRYQNMFVLTWVIFLCLVATNSTVNIFVYYNMSSKFREILNDMLNIKRTET

>23b (AASC01196016)
MASGNSTTLDETALTGIVSDSLTNIFVVLFFVSITGTNSFCGIVFNIINIIVFLKQGFKDSVNITLFG
MAISDTGSLIGLVWVSIFLNPWLVNSGVTYDSTDVHYLTGGWPHMIFARIISWITAFVTFERCLCIAL
PLKVKTIITPRRTIAIVIGIYLIMFAFVTPVFYSVRLGPKYFPKKNQTLIGIIYIPNGPTFENVSFAV
NVLAKFGSFFAVIICTVVLVHNFLLKSKWRQSTSTSAKKESLSQRDKKVVKVVLFIATIFIACFSPGA
ASVVAISNVPGYSITGRYRNMFMVTWSIFTTIQATNSAVNIFVYYNMSSKYKEILDGMLRRKGGDGDK

>24b (AASC01025013)
MSAENKSLATAPTVFVDDDVLNIFVLVFLVIVSGVISFFGIMFNSFNVIVFYKQGFKDTVNITLFALA
ISDTGVLVTHLWMSICYNPQFTSADLPFDYKDIYYLTSGWPNICFSRITAWLTALATIERCLCVTLTL
KVKTILTPRTTSIVVVAIYFSTIAATTPVFYSVRLGPKHTPQINETKIGLFYIPNGVFIESISSFIHT
ISQCISFILVFVCTVILVITLRRNSKWRQSTSSSAKQENVLSKDKKVIKMILTISIIFISCYFPALVN
ALATLIFVEYTVFGAYSNLFLLNWGLAFTITAANSAINIFVYFNMSKKFRQILKEMLHVKKEVRE

>27b (AASC01064229)
MSAVNKSLATATTGLVDNDVLNILVLVFLVIASGVICFFGIIFNFINVIVFYKQGFKDTVNITLFSLA
ISDIGVLVTHLWMSICYNPQFASADLPFFYLDIHYFTSGWPNICFSRITAWLTAFVTFERCLCVALPL
KVKTIPTPRRTLFVVVAIYFTKIAATAPVYYSIRLGPKYIPQINETKIGLFYVPNGDFIESISSIIHT
ISQFVSSIIVFVCTVILVFTLRRNSKWRQSTSSSGKQGDILSKDQKIIEMILSISIIFISCFLPGLAN
AFGKLISVEYSVVGAYSNLFTLTWGLSFTITAGNSAVNIFVYFNMSSNFRQILKEMLQINKELRE

>29b (AASC01193148)
MGPGNNSQARSSESTQKGLLDDYTLAVLSLILYVFAIGIVSVCGLICNVINIIVFCKQGFKDTVNITL
FGLTISDMGCAITLFWGSVCFNPLFIEADLPMVYQDIIYLTSGWPLVCFARISSWITAFVTFERCLCI
TVPLKVKMILTPRRTVFVVVGIYLGIILCVVPLYYAMGLGPRHFPERNVTIIGSVYNENGPFYEGVAL
TLSAFSQLASFFAVIICTGILVHNFLLKSKWRQSASSATRQEFLTNRDKKVVKMILFISSLFIVFFSP
TAANTFVMMISSEYRTGGRYQNVYLLNWAISCLLVGTNSTVNIFVYYSMSSKYRKILDEMLKRKEGNR
GPSERKAVRCIQSL

>30b (AASC01085458)

MDAQNQDLGTTPESAQRGLIDDHQFHIFFIILYVVLGGMISFFGVICNIINVIVFLKQGFKSTVNITL
FGLAISDMGGLLFMLWYSICVNPILNGADLPFNTRDVAYLTAGWPRFCFNRITGWLTAYVTFERCLCV
ALPLKVKTLITQKRTILIVVGIYLAVTASVAPVFFAVRLGPKYFPDSNVTKIGLVHNPNSQFNESISA
TINSFSYLASLFIVILGTAVLIQNILRKSKWRQSTSSSRGQESFLNKDKKVVKMILLLAIIFISCSIP
SVASVIVWILVPEIRASGRYANLALVFHGIIRILEAVNSSVNILVYYNMSSKYREIFDQMLHRIARKR
STQTEQ

>31b (AASC01201741)
MDAENHDLGTTPETAQRGLIDNHVLNICVIILFVAIAGVISFFGAICNIINVIIFLKQGFKNTVNITL
FGLAISDLGGLLFMFWYSICVNPILNGADLPFYTLDVAYLTAGWPRFCFSRITGWLTAYVTFERCLCV
ALPLKVKTLITKRRTTSAVVAIYLAVTASVAPVFYAVRLGPKYFPHTNVTKIGLVHNPNSQFSESISA
TINSFSYLASLFTVVLCTAVLIHNILRKSKWRQSTSSSKGQESSLNKDKKVVKMILLISIIFISCSIP
SVASVIGWILVPEIRAGGRYANLALLFHGIIRILEAVNSSVNIFVYYNMSSKYRALFDQMLQRIPRKG
NTQVEQ

>34b (AASC01091901)
MNAENKDVLTSPESGQEGILDDHLINIFIIISSVVLSGIISFFGIICNIINVIVFLKQGFKNTVNVTL
FGLAISDMGGLLFMFWYSICVNPVLNGVDLPFHIQDVIYITAAWPSLCFSRITGWLTAFVTFERCLCV
ALPLKVKTIITKRRTIFVIVGIYLAATASIAPEFYAARLEPKNFLHINVTKIGLIYTPKGKNIKKILR
KINLFSYLASLIIIILCTAILIQNILRKSKWRQSTSGSCRQESFLTRDKKVVKMILLISIIFISCSVP
GAVGLIVTILILEFRAASRFANLIQLYFGIARILEGVNSTVNIFVYYNMSSKYREIVDQMFHRIVRKR
NIQVEQ

>37b (AASC01203463)
MDAKYQDVGTTPESVQSGFIDDYLINIFVIILNVVLLGIISFFGVICNIINIIVFLKQGFKNTVNITL
FAMSLSDIGGLLFMLWFSICVNPVLLAGTDLPFHMRGVTYITATWPSFCFARITGWLTAFVTFERCLC
VALPLKVKTVITKRRTIFVVVGIYLAVAASVSPVFFAARLGPKYLPHRNATKTGLIYMYTSNGQFIRK
ISTSIYFFSCQASFFTVILCTAVLILNILRKSKWRQSTSSSAVHGSSLNRDKKVVKMILILSIIFISC
SVPGAVGVLCQILIPELSPAGRYANLVTLCLGIIRILEGVNSMVNIFVYYHMSSKYRKIADQMWHRIT
RRQNTQVEQ

>38b (AASC01160578)
MASGNESYGTSFGPRPSKMVNDLVLTIFVIGFFVIILGAISLSGIVTNVINLIVFIRQGFKDNVNISL
FSLAISDIGALLPLLWVSIGFNPLFVNSDLPFDTVEVLHLTSGWPHICFARISGWITAFIMLERCLCI
AVPLKVKTMITVKRIVIVLVSIYVAMIGALLPVFYALRLEPRYSPLKNETKFRMVYIPNGFAIESAVF
LINAFSQLITFSMVIICSTILVQNLISKSKWRKSTSSSGDHDSFSNRDKKVVKLVLFIAIIFIVCLFP
SVINFIAQSLEPEYTASGKYGDLYLLTWSFSVSLSATNSAVNIFVYYNMSSKYKQILDEMLLRRSKG

>10c (AASC01111921)
MTISTVEKMSNISFDVRTHLSTGDGFVSDEAKVYVDIFLGLISLPIISLSGVISNFINMVIFTKSGLT
DCVTLAIFFLAISDFCISFFTFFHTMCFFVMKFAPTTMFDFFSLYFISAWTRSMFADVSLLLTVFVSL
ERCLCVIRPLTFKLIFTAKTSVIMCGVISSVTVLSYGPVMASQGVRWEFDDVTNQTRLMLWLADYRPE
VVKYSNILHSITLAKAALISITISAIVMAIRLRASSKFRKSATSAADSSSKENSLSKTDNRVVMMVFM
VAVVCIVLYSPTLAFVYARRIFPDFAFKNKYRNIFPTIMMIIFQFSTLNASINIVVYYKFNTKYRQLF
LRFYCCSKLQEKPEATR

>11c (AASC01161139)
MSSNDSESGDIRGYMSEAGSKFIINDHVQWYIDIAFLFVSLPFFSVSGILFNITNVIVFTKTGLSDCV
TMAVLFLATSDFLLCFTTLFNVLSSVTKADNSYIPGSYGLYNYLFLLSTWTREMFVDTSLLLTVFVSL
ERCLCVTWPLKFKTLFTTKKAAVISTAITCGTIVSYIPVQLSQGIREQYDVTTNRTHLMLWFSEDRGE
IIKYSNSFHRLFLHNASLASITTSAILMAFRLRASAGFRQRATSAEPGDKNNTRALSRKDSRVVTMVF
LVALASIILLLPSMLFVSARRVLPDLGQQPRFMNLISTLMEIMFIFSTLNANVNIFIYYNFNSRYRQY
LQQYLQCQKVK

>Sm_128710 (Smp_128710)
MDDTNNNYEKYHLLHNIIGCNISFHSCFISITTPSPSISSRSLYSNNSSSTVFTSNGCPT

IPSLKQFEIFYSTIHAYLACTVCLFGTGTNLLNSIILTQEQMRNPTNTLLTGIAIVDSLT
MLAYGLQVIYLHFITSPYPDKYPHSQLAVYLILINHAISIGSHTISTCLLVQLAAFRCWV
LYSRKLKYLLSSHHHLTWIMISSQGRFIIYCYISAGIIGCLLCMPTFILYQCELIDVRNA
SVTLKTKEYEGLGNTNNNFNDDDNNNIKIINVETNTTINNDSTITTNSLRPITCNNPSNS
TVMTRNSDLNNSDYINDLRIRSHQHRINTNITTSNINLLSTVTTTTTTRNPLFLQISSLA
PSNELRNNELNNSSSRNSSNNNNNNNLMITRNRRHSSRLLQKSRESQHVTIMLIIIVISF
VITEAPQGVFNALVAIKGECFLHTIYLPLGDLLDLLVLLNSSTNFILYCAMSQVFRVNFV
NLLKKLLLFTL

>Sm_149170 (Smp_149170)
MNECIPSLLELINQTNPLNTSSSSSSSSSPSNNTEPIINYSLIKFGTEFSKIHGPLSLAV
CLFGIPANFVNIIVLTRRELSQNATNHLLLWLAVADLLLMILYAPCLYHFYIIHPHPMKN
PIYTPSKLWIIYQEIVVSCALMLHSLALWLAVLLALFRYIHVGFPISGSIYTTRKRAFYS
ALLITIFCIFVAIPNILVNHSESCLGPTLNYKSINGGIWNMKNIAPSFLLTILTILLISE
LQKAIKRRKALFIHRDKSLSIIKYNKKQSSLSMRQSTSSPSTLPSTLPLYNNDESKKLSR
ENRTTALLLTIVLCFLVIELPQGILVTCIHLIDQFEEKVYQHLGDLLDFLTLLNESISFI
IYTTMSNQFRQTFCNIFCPILIKNKTSLLNDQQTIIQYRQHHALPSKNNDNNKGRQHSLI
PEEEDLDKGHQQIGTGDSNIK

>Sm_056080 (Smp_056080)
MQRPEFHNLSHCEREKIFIKHFLSTQSTNPIFYAFNSETAKNAVHFATNLNEDNNTSLSK
ILLIDSYVNISTGVISSNLLNQICGPYPTFSELYNDTFDHVVHTYLLPILMIFVLITNFF
VCLVFSKPQMRSPVFFLLFLIGIVELTNCCLPLPMYIAQSVYKRTAWWTDLPIKNFTIAN
NLLNTTYWELAPNDYYQTSDNMIASEASAWSTVFLPTITHTISVWLTVALALQRVIHLTA
FNLTSKICNLSSTGKIFIIISCLAITLHWPLLSSRFILFQPIQLKSSITEDSLHINQRAN
SVQCLWNIVYNLPNIVMKCHLIQPVYMIFYFYARIILIHILPCGILIALTIILVIKMHNI
IKLRKRLGLRQTNDSSSLNHCKCKLFCCVCKRFNSKDPIIDTHNLNNTICILTTKKSSIN
PQAISRMLIVVLIKFIAMHLPNAIVLTIYVLRKMWKVDNSTQTNNEDTLIETTTEEIIQG
LDDDNKQILFTFAYNDTMNRTINLSETNHILIDDYLGKAVILCNLVILVSYQLNFVIYYV
MSTQFKETFNNLCFIKYFTLNRD

>Nv_210893 (jgi|Nemve1|210893|fgenesh1_pg.scaffold_129000019)
MYTSPNLYTARLQKYGTSYKCLTVWAPLDHYTSATVHVVVMVVLFMFIPWLLVATLYLVMVSEIRKSK
REVAQRSRQRNRDLRVLKLALTITAAFAVCNFPICALYLLIAFKYQWNTCFLPEVWGPLFNTAKYLVL
ASTCLNPCICFAFSENFRKGLRELTRAQRSFITTIVTHSRRANSTAFSHIEGEHEMLYEMKENGITEN
GNKNAIDYSPSQSLRE

>Df_F4PUK6 (tr|F4PUK6|F4PUK6_DICFS) (DFA1498372)
MIELEPIEYSTANDSFKGIYFVYAVTRIVVYFLYTVFISILLYKVLRKIYKKRNRQDYTT
DSYQTTRDHTVPIIYLFSIIFLFSRFVAAIISIIDNVRYDPESVTHERLLVAYYSLLVVG
TSSNFISWFEIGYFWILTFYLFYGTIAGGDLYAINPRARIIVDRCLKVTVSILTLVMVAI
VIALNVKWESHEAIDMAYYIVFLGFVLFAGVLFSYHGHHLTQALKSELGKANFGPHEAKK
QEFSASIIRITYLVRLLAIMVIIVIAKTVVLVFLFKRTFGASTLDDFISFVIEMCQTSTI
ILAVHPRRAKALKNGSDTSSGSSGSNNSNKSKGTITGSQTSKKKSASGRSRSATKSSISS
SSSNEDKSGVSSSNNQQQQVVIELGEIESSSSTENQQQIVVVVEPTTSGDLDTRPCLE

Vertebrate olfactory receptors that had two significant Pfam HMM profile hits corresponding to srsx domain (PF10320) and 7tm_4 domain (PF13853), within their transmembrane spanning regions.

>Gg_F1NTA9 (ENSGALP00000005720)
MAEGNSTVVSQFVLLGLTGEPELQTPLFIVLSVIYLITLLGNVGLITLITTSPRLHTPMYFFLCNLSV
VDLCYSSVFSPRLLIGFLLDNKTISYSACFTQHFFFLVFVTTEVFLLAVMAYDRYVAICNPLLYTISM
PKSLCMKLVAGSYLGGILNSLTQTCCLLPLPFCGPNVINHYFCDTNPLLKLTCSDGRLNELLLVTFNG
TISMTVLLIIVISYVYILVSILSIRSARGRHKAFSTCASHLLTVTLFYVPAGLSHMQPGSKYSLDMEK
VTAVFYTLLVPMLNPLIYSLRNKEVKDTLRRARERIFSQA

>Mm_Olfr631 (ENSMUSP00000102476)
MVLSNITHFSPMFYLSGFPGLEAFEHWIFIPFFLMYLVAISGNCLILIIIKTNPRLHTPM
YYLLSLLALTDLGLSVSTLPTMVGIFWFNYHGIYFGACQIQMFCIHSFSFMESAVLLVMS
FDRFVAICHPLRYSSIITVQRVMRAGLCVILRGPVALIPIVLLLKDFPYCGPLVLSHSFC
LHQEVIHLACVDTTFNNLYGLSLVVFTVMLDLVLIALSYGFILYTVAGLASQEEQIRAFQ
TCTSHLCAVLVFFVPMMGLSLVHRFGKHAPPAVHLLMANIYLFVPPMLNPVIYSIKTKEI
RKAIIRFLGFRKVNSESWG

>Mm_Olfr1381 (ENSMUSP00000126003)
MEYLNTSSEEGFILVGFSDWPHLEPTLFAFISIFYSLTLFGNTVIIILSRLDLRLHTPMY
YFLCHLSFLDLCYTTSTVPQLLVNLSGLDRTISFGRCVAQLCIVLSLGGTECVLLVTMAI
DRYAAVCRPLHYTTIMHPVLCRALVVFSWVGGLVNSLIQTSLVMAMPLCGHQLNHFFCEL
PVLLKMACEDTGGTEVNLFVARVIILVCPLLLILGSYAHIARAVLNIRSMAGRRKAFGTC
ASHLIVVAMFYGSAISTYLQPVHRYSEKEGKFLALFYTIITPMLNPLIYTLRNKDVKGAL
WKVLGRGTDSR

>Mm_Olfr432 (ENSMUSP00000060341)
MDHVNYTWTRTFILAGFTTSGTLQHLAVFGTLCIYLLTLAGNLFIIVLIQADSGLSTPMY
FFISVLSFLELWYVSTTVPTLLHTLLHGPSPIPSSACFVQLYVFHSLGMTECYLLGVMAL
DRYLAICRPLHYHALMSRQVQKQLVGVTWLAGFSAALVPAGLTASLPYCLKEVAHYFCDL
APVMQLACVDTSWHARLYIAVIGMINTCNLTFILGLYGGIVRAVLKLPSAASRAKAFSTC
SSHITVVTLFFGSAFIVYVGPPEIRAEGRDKLIALVYTLLTPFFNPIIYTLRNKEVKEAF
KRVTQRINAVLK

>Mm_Olfr1344 (ENSMUSP00000130328)
MGPGTLNDSGTTEFLLLGLWAPPSLRPLLWASLLLAYLTTVLGNGALVGLIALDRRLHRP
MYRLLTHLALLDTAYVSTTLPQALAHMTMRSARLSLVRCGTQLYVGISLGSCEAILLAAM
ALDRCLAVCRPLHYATLVTAPRCAALAGASWTLGFALSVPNAVAALRLPFCPGRAAVDHF
FCELPAVLRTACADTTANYRLVYGLGVPILLVPLVLILASYTWILAAVRKLPSAGSRHKA
LSTCSSHLAVVGLFYGTVSAMYLRPKASSDLPARHHKLVAVFYLVVTPVLNPLIYSLRNR
EVHMAARYALARLRGTRIVLH

>Mm_Olfr117 (ENSMUSP00000073320)
MTINKSSGGDFILVGFSDQPQLEKILFVLVLISYLLTLVGNTAIILVSCLDSALQTPMYY
FLTNLSFVDICFSTSIVPQLLWNLHGPAKTITATGCAIQLYVSLALGSTECVLLAVMAFD
RYAAVCRPLHYATVMHPRLCQSLAGVAWLSGVGNTLIQGTITLRLPRCGNHKIYHFICEV
PAMIKLACVDIHANEVQLFMASLVLLLLPLTLILVSYGYIAQALMRLRSALTWGKALGTC
GSHLIVVVLFYGTSTAVYIHPNSSYAQSQGKFITLLYTVVIPTLNPLIYTLRNKDVKGAL
KRLVRKDSSTGKKILSR

>Mm_Olfr68 (ENSMUSP00000072329)
MIKFNGSVFMPSVLTLVGIPGLESVQCWIGIPFCVMYIIAMIGNSLILVIIKSEKSLHIP
MYIFLAILAVTDIALSTCILPKMLGIFWFHMPQIFFDACLLQMELIHSFQATESGILLAM
ALDRYVAICNPLRHATIFSPQLTTCLGAGALLRAFILVSPSILLIKCRLKYFQTTIISHS
YCEHMAIVKLAAQDIRINKICGLLVAFAILGFDIVFITFSYVRIFITVFQLPQKEARFKA
FNTCIAHICVFLQFYLLAFFSFFTHRFGAHIPPYVHILLSDLYLLVPPFLNPIVYGVKTK
QIRDQVLKMLFSKKH

>Mm_Olfr46 (ENSMUSP00000072445)
MNWILHSTMVSPNQTVVTEFVLQGFSEHPSLRLFLMGCFLSLYTVALMGNMVIIALITSS
TGLHSPMYFFLCNLATMDIICTSSVLPKALVGLLSEENTISFKGCMTQLFFLVWSGSSEL
LLLTVMAYDRYVAICLPLHYSSRMSPQLCGTFAVGVWSICALNASINTGLMTRLSFCGPK
VITHFFCEIPPLLLLSCSPTYINSVMTLVADAFYGGINFLLTLLSYGCIIASILRMRSAE
GKRKAFSTCSSHLIVVSVYYSSVFCAYISPGSSYSPERSKFTSVLYSVLSPTLNPLIYTL
RNKDVKLALRRLFPSFSN

>Mm_Olfr1361 (ENSMUSP00000062356)
MWLQVMEKENTSSFEGFILVGFSDRPHLELILFVVVLSFYLLTLLGNMTIILLSALDSRL
HTPMYFFLANLSFLDMCFTTGSIPQMLYNLWGPDKTISYVGCAIQLYFVLALGGVECVLL
AVMAYDRYAAVCKPLHYTVIMHPRLCGQLASVAWLSGFGNSLIMAPQTLMLPRCGHRRVD
HFLCEMPALIGMACVDTMALEALAFALAIFIILAPLILILISYGYIARAVFRIKSAAGRR
KAFNTCSSHLIVVSLFYGTIIYMYLQPANTYSQDQGKFLTLFYTIVTPSVNPLIYTLRNK
DVKEAVKKVLGKGSIEV

>Mm_Olfr554 (ENSMUSP00000095824)
MSTFHNVCSVPSSLWLTGIPGLETLHIWLSIPFGSMYLVAVVGNITILAVVRVERSLHQP
MYFFLCMLAVIDLVLSTSTMPKLLAIFWFGAGHIGLDACLCQMFLIHCFATVESGIFLAM
AFDRYVAICNPLRHSMVLTHTVVGRLGLAAVLRGVLYIGPLPLMIRLRLPLYKTRVISHS
YCEHMAVVALTCGDSRVNNVYGLSIGFLVLILDSAAIAASYVMIFRAVMGLATPEARLKT
LGTCGSHICAILIFYVPIAVSSLIHRFGHQVPPPIHTLLANFYLLIPPILNPIVYAVRTK
QIRDRLLQILKTGTKIR

>Mm_Olfr553 (ENSMUSP00000095825)
MYPTLPSMFVSNNACSVPSSFWLTGIPGLESLHMWLSIPFGSMYLVAVVGNITILAVVKT
ERSLHQPMYFFLCMLAVIDLVLSTSTMPKLLAIFWFGACSIGLDACLVQMFFVHCFATVE
SGIFLAMAFDRYVAICDPLHHTSVLTHAVVGRLGLAALLRGVFYIGPLPLLIRLRLPFFR
TQIIAHSYCEHMAVVTLACGDTKVNNLYGMGIGFLVLILDSIAITASYIMIFRAVLGLST
SDARFKTLGTCGSHICAILVFYIPIAVSSLTHRFGHNVPSHIHILLANFYLLIPPILNPV
VYAVRTKQIRERLLHIIKSGTQHKDM

>Mm_Olfr551 (ENSMUSP00000095827)
MQHTNHSHQNPSSFLLMGIPGLEASHFWIAFPFCSMYALAVLGNMAVLLVVRSEPSLHQP
MYLFLCMLSTIDLILCTSTVPKLLALFWANAAEIAFGACATQMFFIHGFSAVESGILLSM
AFDRYLAICRPLHYGSLLSSESVSKLGAAALLRGLGLMTPLTCLLARLSYCGRVVAHSYC
EHMAVVKLACGGTQPNNIYGITAATLVVGTDSICIAISYALILRAVLGLSSKEARAKTFG
TCGSHLGVILLFYTPGLFSFYTQRFGQHVPRHVHILLADLYLVVPPMLNPIIYGMKTKQI
RDGALRLLKRGPAQS

>Mm_Olfr550 (ENSMUSP00000095828)
MFCNTSTPGHSTFLLTGFPGLEASHHWVSIPINLICVVSILGNSVILFLIRTDPALHEPM
FIFLSMLAASDLGLCASTFPTMVRLFWLGTRELPFDFCAAQMFFIHAFTYVESGVLLAMA
FDRFIAIRDPLHYATILPHSAVAKVGAAVLVRAILLNLPGPILLRRLIFPQISTLSHCYC
LHCDLVGLACSDTRINSLVGLVSILLSLGLDSSLIMLSYALILRTVLGIASPGERLKALN
TCVSHLCIVLIFYLPKLGLSVLHRVEKHSYPALAVLMANLHFLVPPFMNPVVYCIKSKQI
RQGFLRRFQQKRVDIS

>Mm_Olfr10 (ENSMUSP00000073963)
MGTFNISLGGGFILVGFSDWPALELIFFIHILIFYSITLFGNTAIIALSRTDLRLHTPMY
FFLSHLSFLDLCFTTSTVPQLLINLHGQDRTISYGGCVAQVFIFLALGSTESVLLVVMAF
DRYAAVCRPLHYTTIMHPVLCQALAIASWVGGFLNSLIQTGLMMAMPLCGHRLNHFFCEM
PVFLKLVCEDTGGTEAKMFVARAVIVAVPTMLILGSYAQIARAVLKVKSVTARRKAAGTC
GSHLLVVSLFYGSAIYTYLQPKDSYSESKGKFVALFYTIITPMLNPLIYTLRNKDMKGAL
WKVLGRATVTG

>Mm_Olfr684 (ENSMUSP00000055880)
MALSNDSEAPISEFLLICFPNYQTWQHWLSLPLSLLFLLAMGANATLLITIRLEASLHEP
MYYLLSLLSLLDIVLCLTVIPKVLAIFWFDNKSIGFSSCFLQMFVMNSFLTMESCTFMVM
AYDRYVAICKPLQYPTVITDQFVVRAAIFIISRNALISLPVPILSARLKYCAQNIIKNCI
CTNLSVSRLSCDDITLNKLYQLVAGWTLLGSDLILIVLSYSFIFRVVLRIKAEGAVAKAL
STCGSHFILILFFSTVLLVLVITNLARERIPPDIPILLNILHHLIPPALNPIVYGVRTRE
IKQGIQNLLRRL

>Mm_Olfr94 (ENSMUSP00000055232)
MWLCNKTKTWACAEFIPYWRFLFVVVSGKTGFYYVALAGLELTEISGLCLPAQGPQHCLA
LNIFVSPSEPSWSFPPQANHSSAERFLLLGFSDWPSLQPVLFALVLLCYLLTLTGNAALV
LLAIRDPRLHTPMYYFLCHLALVDVGFTTSVVPPLLASLRGSMLQLPRAGCMAQLCSSLA
LGSAECVLLAVMALDRAAAVCNPLRYTSLASPLLCRTLAGVSWLGGLANSAAQTALLAAR
PLCAPRCLDHFICELPALLQLACRGGRSATERQMFAARVVILLVPSAVILASYIAVGRAV
WGMHSSSGWRKAASTCGSHLTAVCLFYGSATYTYLQPTHSYNQGRGKFVSLFYTVVTPAL
NPLIYTLRNKEVKGAALRLLRSLGRP

>Mm_Olfr1384 (ENSMUSP00000051954)
MGSFNLSLEGFLLVGFSDWPQLELILLVFISIFYSLTLCGNITIIVLTQQDLHLHTPMYF
FLAHLSFLDLCFTSSTVPKLLISLSRGDQTISYAGCMTQFFIALLLGGTECVLLVVMAFD
RYVAVCRPLHYTSIMHPLLCHALAISSWVGGLVNSLTQTSLIMTIPLCGHHLNHFFCEML
VLLKLACEDTVGTETYLFWAGAVILVCPVALILGTYAHIAHAVLKIKSRSGRRKALGTCG
SHLTVVFLFYGSAMYTYLQPIHTYSGSEGKFAALFYTIITPMLNPLIYTLRNKDVKGALC
KVLVKGKKETKTRRKMVE

>Mm_Olfr564 (ENSMUSP00000129376)
MSTFQNTTASSIIFLLTGVPGLEAFHTWISIPFCFLYATALSGNSLILFVIITQPSLHEP
MCYFLSMLSTTDLGLSISTLATMLGIFWFNAREISFNACLSQMFFIQLFTVMESSVLLAM
AFDRYVAISNPLRYASVLTDLKIAQIGVAIVTRGTLILTPMVVLLKRLSYCSSHVLHHSY
CFHPDVMKLSCTDTRINSAVGLTALISTAGVDSVFIILSYVLIIRTVLSIASPEERKKAF
STCISHIGAVAVFYIPLISLSFVHRFGKRAPPYVHTLIANAYLLIPPVMNPIIYSVKTKK
IRKAVLKVFHSSMTKS

>Mm_Olfr653 (ENSMUSP00000095776)
MMLSAAIPNETAFHPPTFVLLGIPGMQDQHVWIAIPFCSMYILALVGNGTILYIIITDRA
LHEPMYLFLCLLSITDLVLCSTTLPKMLAIFWLRSHVISYHGCLTQMFFVHAVFATESAV
LLAMAFDRYVAICRPLHYTSILNAVVIGKIGLACVTRGLLFVFPFVILIERLPFCGHHII
PHTYCEHMGIAKLACASIKPNTIYGLTVALSVTGMDVVLIATSYILILQAVLRLPSKDAQ
FRAFSTCGAHICVILVFYIPAFFSFFTHRFGHHVPPQVHIILANLYLLVPPVLNPLVYGI
NTKQIRLRILDFFVKRR

>Mm_Olfr53 (ENSMUSP00000081494)
MMFSPNQTEVTEFILEGFSEHATLRLLLTGCFLSLYTIALMGNIVIIALVTSSTGLHSPM
YFFLCNLATMDIVCTSSVIPKALIGLVSEENTISFKGCMAQLFFLLWSLSSELLLLTVMA
YDRYVAICFPLHYSSRMSPQLCGALAVGVWSICAVNASVHTGLMTRLSFCGPKVITHFFC
EIPPLLLLSCSPTYINSVMTLVADAFYGCINFVLTLLSYGCIIASVLRMRSAEGKRKAFS
TCSSHLIVVSVYYSSVFCAYVSPASSYSPERSKVTSVLYSILSPTLNPLIYTLRNKDVKL
ALGRLLPFFPK

>Mm_Olfr61 (ENSMUSP00000081493)
VDPTLSMMLLSLNQTGVTEFVLEGFSEHPGLRLFLTGCFLTLYMMALMGNIVIIALVTSS
TGLHNPMYFFLCNLATTDIVCTSSVIPKALIGLVSEENIITFKGCMAQLFFLAWATSAEL
LLLTVMAYDRYVAICFPLHYSSRMSPQLCGALAVGVWSISAVNASVHTGLMTRLSFCGPK
VITHFFCEIPPLLLLSCSSTYINSVMTLVADVFLGGINFMLTLLSYGFIIASILRMRSAE
GKRKAFSTCSSHLIVVSVYYSSVFCAYISPASSYSPERSKFTSVLYSVVSPTLNPLIYTL
RNKDVKLALGRMLASFSH

>Mm_Olfr45 (ENSMUSP00000081492)
MMLRLNQTEVTEFVLEGFSEHPDLRLFLIGCFLTLYIMALMGNILIIALVTSSTGLHNPM
YFFLCNLATTDILCTSSVIPKALVGLVSEENTISFKECMSQLFFLAWSASSELLLLTVMA
YDRYVAICCPLHYSSRMSPQMCGALAMGVWSISAVNASVHTGLMTRLSFCGPKVITHFFC
EIPPLLLLSCSPTYVNTIMTLLGDSFFGGVNFVLTLLSYGCIIASILRMRSAEGKRKAFS
TCSSHLIVVSVYYSSVFCAYVSPASSYSPERSKVTSVLYSIVSPTLNPLIYTLRNKDVKL
ALGRILASFSH

>Mm_Olfr577 (ENSMUSP00000059586)
MTPGPLGNGSMSSTFLLSGIPGLEHMHIWISLPLCLMYLVSILGNCTILFIIKTEPSLHE
PMYLFLSMLALTDLGLSLCTLPTVLGIFWVGARDISHDACFTQLFFIHCLSFLESSVLLS
MAFDRFVAICRPLHYASILTHTVIVRIGLASLGRSVALIFPLPFMLKRFPYCGSLVLSHS
YCLHQEVMKLACADIKANSIYGMFVIVSTVGVDSLLILFSYALILRTVLSIASRAERLKA
LNTCVSHISAVLLFYTPMIGLSVIHRFGKQAPHLVQVVMGFVYLLFPPVMNPIVYSVKTK
QIRDRVAHAFCN

>Mm_Olfr1383 (ENSMUSP00000059586)
MSSFNTTLKGGFILMGFSDWPQLEHIFFVFISMFYILTIFGNFTIIITISRMDQRLQTPMY
FFLNNLSFLDLCYTTSIVPQLLVNISGIDKTMSYAGCMTQFFIVLLLGGTECMLLVVMAF
DRYVAVCHPLHYTSIMHPLLCHALAISSWVGGLVNSLTQTSLIMTIPLCGHHLNHFFCEM
LVLLKLACEDTGGTEANLFVAGAVILVCPVALILGTYAHIAHAVLKIKSRSGRRKALGTC
GSHLTVVFLFYGSAMYTYLQPVHVYSGSEGKFAALFYTIITPMLNPLIYTLRNKDVKGAL
CKVLGRDTSTT

>Mm_Olfr123 (ENSMUSP00000058678)
MINSSVSSDFILVGFSDQPQLERRLFIVVLISYLLTLVGNTIIILISSIDSKLKTPMYFF
LTHLSFVDICFTTSIVPQLLWNLKGPAKTITAVGCAVQLYVSLTLGSTECILLAVMAFDR
YAAVCKPLHYVAVMNPQLCRALAGISWLSGIGNALIQGTITLWLPRCGHLWLHHFFCEVP
SMIKLACVDIHANEVQLFVASLVLLLLPLALILTSYGHIAKAVIRIKSSQAWRRALGTCG
SHLMVVSLFYGSITAIYIQPNSSYAHTHGKFISLFYTVMTPTLNPLIYTLRNKEVKGALG
RLFNRASGV

>Mm_Olfr1385 (ENSMUSP00000071710)
MDSFNATLEERFFLVGFLDWPQLELILFVFISIFYSLTIFGNTAIIALSQMDLRLHTPMY
YFLSHLSFLDLCYTTSTVPQLLINLHGLDRTISYGGCVAQLFISLALGSTECVLLVVMAF
DRYAAVCRPLHYMTIMHPLLCQALALASWVGGFLNSLIQTGLMMAMPLCGHRLNHFFCEM
PVFLKLACQDTGGTEAKMFVARAIILVFPATLILGSYGHIAKAVLKVKSTAGRRKAFGTC
GSHLLVVSLFYGSAIYTYLQPKSSYSESDGKFVALFYTIVTPMLNPLIYTLRNKDVKGAL
WKVLGRGTD

>Mm_Olfr1391 (ENSMUSP00000052092)
MGSFNASLGKGFILVGFSDWPQLELILFIYVLIFYSLTLFGNTTIIALSQLDIRLHTPMY
FFLSHLSFLDLCYTTSTVPQLLINIAAQDHTITYGRCVAQLFSVLALGSTESMLLVVMAF
DRYAAVCRPLHYTTIMHPLLCQALAISSWVAGLVNSLIQTGLMMAMPLCRYRLNHFFCEM
PVFLKLACKDTAGTEAKMFVARAIILVFPATLILGSYAHIARAVLKVKSTSGRRKAFGTC
GSHLLVVSMFYGSTIYTYLQPNDSYSENEGKFVALFYTIVTPMLNPLIYTLRNKDVKGAL
WKVLGRGTDSR

>Mm_Olfr685 (ENSMUSP00000061561)
MALSNSSWRQPQPPFFLVGVPGLEESQHWIALPLGILYLFALVGNVTIIFIIWTDSSLHQ
PMYLFLAMLAAIDLVLASSTAPKALTVLLAHAHEIGYIVCLTQMFFIHAFSSMESGILVA
MALDRYVAICHPLRHSTILHPGIIGRIGLVVLVRGLVLLFPFPILLQNVVFCRATVISHA
YCEHMAVVKLACSETTVNRAYGLSVALLVVGLDVLAIGISYALILQAVLKVPGGEARLKA
FSTCGSHVCVILIFYVPGMFSFLTHRFGHHVPHHVHVLLATLYLLVPPALNPLVYGVKTR
QIRQRVLRVFYTKASI

>Mm_Olfr107 (ENSMUSP00000076267)
MNCSKTPGFILLGLSSDPEKWQPLFNIFLCLYLLGLLGNLLLLLAIGTDVHLHTPMYFFL
SQLSLVDLCFITTTAPKMLEALWTGDGSISFSGCLTQFYFFAVFADMDNLLLAVMAIDRY
AAICHPLFYPFLMTPCRCEVLASGSWGIAHCVSLFYTLLLSQFYYHTNQGIPHFFCDSRP
LLLLSCSDTHLSEGLMMALSGVLGMSSVLCLVSSYGCIFYAVARVPSAQGKRKALATCSS
HLSVVLLFYSTVFATYLKPPSTSHSSAEVVAAVMYTLVTPTLNPFIYSLRNKDVKSSLRK
ILNMDKFQG

>Mm_Olfr615 (ENSMUSP00000095800)
MEISNLSMSEFNTTFQPSVFILTGLRGLVGARLWLGPLLSLMYITTLAGNCTVIYLVRTE
RSLQEPQYQFLSMLAGADIVLSVSTLFSVLKVFIFDLYEIAFDSCLAQLFFIHTSSSMGS
GILLAMAFDRFVAISHPLQYTTILTNSRVTRMGLAAFLRGVALMMPLPILLKRLPFCKGQ
LLSYSYCIHPNVMKLACGQVKINIFYGLVLVIFSFGVDFLLIAISYALIFQAVMGIASRE
GQMKALNTCLSHIFIVFIYYGPLLAITVMHRISRRSSPIAHAVLGNIYLFMPPMLNPIVY
SLKTKQIRSALRKSLKI

>Mm_Olfr606 (ENSMUSP00000095802)
MAPSNSSVSVSSTFYLTGIPGYEEFHHWISIPFCLIYIIGVTGNCSILHIVRTDPKLHEP
MYYFLAMLSLTDMAMSLPAMVSLFRVLWSISREIQFNICVVQMFLIHTFSFTESSVLLAM
ALDRYVAICHPLRYATILTPKLIAKIGIAALLRSAIPLIPLLVRLAFFSFCRSHVLSHSY
CLHQDIIRLACADIRFNVIYGMVVILMLWGMDSLGILITYVFILHSVLRIASREGRLKAL
NTCASHICAVLILYVPMIGLSIVHRFAKHSSPFVHIFMAHIYLMVPPVLNPIIYSVKTKQ
IRQGIFHLICPHKINSSAM

>Mm_Olfr608 (ENSMUSP00000095801)
MVNDTTHHYISFFYLVGIPGFENFHYLISIPVCLLFVLTLLGNSIVIATIKLEPSLHQPM
YFFLCMLAMNDILLTCSTSLKMLGIFWFNEHWIEFDVCLTQMYFIHTLCIFESAILVAMA
FDRFVAICIPLHYATILTTAMVIKLGVVGLSRALLMVLPCPLLIKRLPYYTGYIIPHTYC
EHMAVVKLASANTFINRAYGISAALSVITLDVWLIAASYIKILQAVFRLSSQNARSKALG
TCAAHVCTILAFYTPALFSFLTHRIGKNVPPSVHIILASMYLLVPPTVNPLVYGVKTKQI
RDRVLSLFSHLKIAEY

>Mm_Olfr978 (ENSMUSP00000060106)
MSNTSIVTTFFLSGLPHPPVLDSMLFGIFLVIYILTVLGNLLILTVIRVDSHLHTPMYYF
LTNLSFIDMWFSTVTVPKMLMTLVSTGGGAISFHSCVAQLYCFHFLGSTECFLYTVMSYD
RYLAISYPLRYSSMMGGRMCALLAAGTWFTGSLHSAVQTTLTFHLPYCGPNQIQHYFCDA
PPILKLACADTSANEMVIFVNIGVVASGCFFLISLSYVSIVCSILRIRTSEGRHRAFQTC
ASHCIVVLCFFGPGLFIYLRPGSRDAVDGIVAVFYTVLTPLLNPVVYTLRNKEVKKALLK
IKYGSVLPQDK

>Mm_Olfr541 (ENSMUSP00000079508)
MMMLRLNQTEVTEFVLEGFSEHPDLRLFLIGCFLSLYMMALMGNIVIIALVTSSTGLHSP
MYFFLCNLATMDIVCTSSVIPKALVGLVSGENTISFKGCMAQLFFLVWSASSELLLLTVM
AYDRYVAICFPLHYSSRMSPQLCGALAVGVWSICALNASVHTGLMTRLSFCGPKIITHFF
CEIPPLLLLSCSPTYINSVMTLVADAFYGGINFVLTLLSYGYIIGSILRMRSAEGKRKAF
STCSSHLIVVSVYYSSVFCAYVSPASSYSPERSKVSSVLYSVLSPTLNPLIYTLRNKDVK
LALGRILPSFSH

>Mm_Olfr686 (ENSMUSP00000049601)
MIFSNNSHLLPHTFFLTGIPGLTAAHVWISLPFCFMFVLSLTGNAVLLSLIWIEHRLHQP
MFLFLAMLSFVDLVLSLSTLPKMLAIFWFGATAISSYACLSQMFLIHAFSAMESGVLVAM
ALDRFVAICNPLHYATILTPEVVAKIGGLVALRGVGLTIFFPSLACRLSYCGSHTIAYTY
CEHMSVVKLACGAITVDSLYAFAVAIFLGVGDMAFIAYSYGQIVKTVMRFPSPEARGKAG
STCTAHVCVILFFYGPGFLSVVMQRFGPSTASAAKVILANLYLLFPPALDPIVYGVKTKQ
IRECLFTIIGSKKIEPT

>Mm_Olfr611 (ENSMUSP00000077246)
MLHVNITNSIFSTFLVTGIPGLEAVYIWIAIPFCAMFLITMVGNMTIIIVIWHEQTLHVP
MYLFLAMLASSDLGLSLFTFPTLLRIFLLNDRELTTTACFTQMFFIHTFQDLESAIILAM
AFDRYVAISHPLHYHSILTDTVIAKIGLAIVVRTLTLQVPAPILLRRLYFCRSNVLSHSY
CLHPDIIKLSCSSTTVNSIFGLFVVLSTLGLDFLLILLSYALILKTVLSMASHSGRLKAL
NTCISHLCAVVLFFTPMICLSMLHRFGPRLPSHVYVTLANMHFLIPPVMNPIVYVVKTKQ
IRDKIQKLFIRKATKKAQAASIT

>Mm_Olfr632 (ENSMUSP00000095791)
MKVSIPPRANFSYAIFLLTGFPGLEWAHHWISLPIFMGYFVAIMGNATILHLVRTDPSLH
QPMYYFLAILAVTDLGLCMSTLPSVLGVLWFDARMVGLVPCVLQQHFLHSFSFMESAVLF
AMALDRLIAIRFPLRYASVLTGPRVALIGTVLGMRSAAITAAPSLHLLTFDYCHPGALSH
AYCLHQDMIRLACSDTRFNRLYGLCIIMLAMGSDVLFILLSYAVILRTVLAIASAGERLK
ALNTCVSHILAVLCFYVPVLGLSIVHRFGQHTSPLVHILMGTVSVLFPPVMNPVIYSIKT
QQIRRAIVKVISLGKIQ

>Mm_Olfr633 (ENSMUSP00000095790)
MFNSSQFTPKYFLLTGFPGLEAWYPWLIFPFCFTYTIGIMGNTLILAVIRKNSSLHQPMF
LFLAMLAFSELGVSASTLPTVLSIFLLGANKICFEACLLQMFSIHSFSIMESGVLLAMSV
DRFVAIYNPLRYTAILTLPRIAGTSLTLGLKSLLLMFPLPFLLKRLPFCGHNVLSHSYCL
HSDLIQLPCGDTRPNSILGLCIVTSTFGLDSLLIVVSYVLILYTVLNITSGEGRRKALNT
CVSHMCAVLVYYVPMISVALVHRFMKHAVPAVRLLLANIYLLVPPMLNPIIYSAKNRQIR
QGLIQLFLQRKY

>Mm_Olfr60 (ENSMUSP00000074916)
MMSRLNQTVVTEFILQGFSEHPSLRLFLTGCFLSLYVMALMGNILIIALVTFSTGLHSPM
YFFLCNLATMDIICTSSVLPKALVGLLSEENTISFKGCMAQLFFLVWSLSSELLLLTVMA
YDRYVAICFPLHYSSRMSPQLCGALAMGVWSICALNASINTGLMTRLSFCGPKVITHFFC
EIPPLLLLSCSPTYVNSIMTLIADVFYGGINFVLTLLSYGCIIASILRMRSAEGKRKAFS
TCSSHLIVVSVYYSSVFCAYVSPASSYSPERSKVTSVLYSFLSPTLNPLIYTLRNKDVKL
AIGRLLPSFSH

>Mm_Olfr97 (ENSMUSP00000094937)
MMNCSQAPGFILLGLSSNSEKWQPLFSIFLVLYLLGLLGNLLLLLAIGTDVHLHTPMYFF
LSQLSLVDLCFITTTAPKMLETLWTGDGSISFSGCLTQLYFFAVFADMDNLLLAVMAIDR
YAAICHPLLYPLLMTPCRCRVLVSGSWGVAHCVSLTHILLLSQLYFHTNQEIPHFFCDFG
PLLLLSCSDAHLNESLMMALAGVLGISALLCIVSSYGCIFYAVAKVPSAQGKRKALATCS
SHLSVVLLFYSTVFATYLKPPSSSRSSGEVVAAVMYTLVTPTLNPFIYSLRNKDVKSSLR
RILNMVKSQD

>Mm_Olfr1367 (ENSMUSP00000055870)
MSVNRISADFPEDFILMGFTKYPWLDLPLFFVLLTSYMFTLLGNIAIILVSQLDSQLQSP
MYFFLTSLSFLDLCFTTTTVPQMLFNLQGPNKNITYIGCMAQAYVFHWLGCTECVLLGIM
ALDRYVAVCKPLRYSVIMDHRLCLQLSGAAWLTGLANSLLQSTLTIQLPLCGNRMLDHFF
CELPGLIKMSCGDTTVNEVTLAVVATFFIMGPLSMILVSYSYIAQTVFRMPSAAGRLKAF
NTCSSHLLVVSLFYGPGIYIYMQPSEDGSQDLIKVLTLFYCVITPMANPFIYTLRNKDVI
GALKRLLRKAISTKGI

>Mm_Olfr1387 (ENSMUSP00000073494)
MGNFNTSTQESFILVGFSDWPQLQAFLFVIILIFYSLTIFGNTTIIVLARLDLRLHKPMY
FFLSHLSFLDLCYTTSTVPQLLINLRGLDRTISYGGCVAQLFIFLALASTECLILVAMAF
DRYAAVCHPLHYTSIMNPILCRALAISSWVGGLVNSLIQTGLVMAMRLCGHQINHFFCEM
PIFLKLACEDTEGTEAKMFVARTIVLVCPAVLILGSYVHIAKAVLKVKSMAGRRKAFGTC
GSHLMVVSLFYGSGIYTYLQPVHRYSESKGKFVALFYTIVTPMFNPLIYTLRNKDVKGAL
WKLLGRGTDSG

>Mm_Olfr609 (ENSMUSP00000051749)
MSYSNHSSTSFFLTGLPGLETVYLWLSIPLCTMYIASLAGNGLILWVVKSEPSLHQPMYY
FLSMLAVTDLGLSVSTLPTMLTIYMMGVSEVALDMCLAQLFFIHTFSIMESSVLLTMAFD
RVVAISSPLHYATILTNPRVASLGMVILVRSIGLHIPAPIMLKKLPYCQKRHLSHSYCLH
PDVMKLACTDTRINSAYGLFVVLSTLGVDSVLIVLSYGLILYTVLSIASKTERLKALNTC
VSHICSVLLFYTPMIGLSMIHRFGKWASPCSRVLLSYLHFLTPPVLNPVVYTIKTKQIRQ
RIWRIFRCGGRSIGHIQGH

>Mm_Olfr1388 (ENSMUSP00000053834)
MGTFNFSSDRAFFLVGFSDWPHLELVFFVAISIFYSLTLFGNSSIIALSRLDLRLQTPMY
FFLCHLSFLDLCYTTSTVPQLLINLHGQDRTISYERCVAQLLIFLALASTECVLLGVMAF
DRYAAVCRPLHYTTIMHPQLCHTLAISSWVGGLVNSLTQTGLMMVMPLCGYRLNHFFCEM
PIFLKLACEETKRTEAKMFVARTIVLVCPAALILGSYAHITRAVLKVKSTAGRRKAFGTC
GSHILVVSLFYGSAIYTYLQPTHTYSESEGKFVALFYTIITPMLNPLIYTLRNKDVKGAL
WKVLGRGTDSE

>Hs_OR51C1P (ENSP00000350408)
MTNNITSTSIIFLLTGVPGLEAFHTWISIPFCFLSVTALLGNSLILFATITQPSLHEPMY
YFLSMLSATDLGLSISTLVTMLSIFWFNVREISFNACLSHMFFIKFFTVMESSVLLAMAF
DRFVAVSNPLRYAMILTDSRIAQIGVASVIRGLLMLTPMVALLIRLSYCHSQVLHHSYCY
HPDVMKLSCTDTRINSAVGLTAMFSTVGVDLLLILLSYVLIIRTVLSVASPEERKETFST
CVSHIVAFAIYYIPLISLSIVHRFGKQAPAYVHTMIANTYLLISPLMNPVIYSVKTKQIR
RAVIKILHSKET

>Hs_OR6V1 (ENSP00000396085)
MANLSQPSEFVLLGFSSFGELQALLYGPFLMLYLLAFMGNTIIIVMVIADTHLHTPMYFF
LGNFSLLEILVTMTAVPRMLSDLLVPHKVITFTGCMVQFYFHFSLGSTSFLILTDMALDR
FVAICHPLRYGTLMSRAMCVQLAGAAWAAPFLAMVPTVLSRAHLDYCHGDVINHFFCDNE
PLLQLSCSDTRLLEFWDFLMALTFVLSSFLVTLISYGYIVTTVLRIPSASSCQKAFSTCG
SHLTLVFIGYSSTIFLYVRPGKAHSVQVRKVVALVTSVLTPFLNPFILTFCNQTVKTVLQ
GQMQRLKGLCKAQ

>Hs_OR51F2 (ENSP00000323952)
MTETSLSSQCFPMSVLNNTIAEPLIFLLMGIPGLKATQYWISIPFCLLYVVAVSGNSMIL
FVVLCERSLHKPMYYFLSMLSATDLSLSLCTLSTTLGVFWFEAREINLNACIAQMFFLHG
FTFMESGVLLAMAFDRFVAICYPLRYTTILTNARIAKIGMSMLIRNVAVMLPVMLFVKRL
SFCSSMVLSHSYCYHVDLIQLSCTDNRINSILGLFALLSTTGFDCPCILLSYILIIRSVL
SIASSEERRKAFNTCTSHISAVSIFYLPLISLSLVHRYGHSAPPFVHIIMANVFLLIPPV
LNPIIYSVKIKQIQKAIIKVLIQKHSKSNHQLFLIRDKAIYE

>Hs_OR51H1P (ENSP00000322724)
MTNLNASQANHRNFILTGIPGTPDKNPWLAFPLGFLYTLTLLGNGTILAVIKVEPSLHEP
TYYFLSILALTDVSLSMSTLPSMLSIYWFNAPQIVFDACIMQMFFIHVFGIVESGVLVSM
AFDRFVAIRNPLHYVSILTHDVIRKTGIAVLTRAVCVVFPVPFLIKCLPFCHSNVLSHSY
CLHQNMMRLACASTRINSLYGLIVVIFTLGLDVLLTLLSYVLTLKTVLGIVSRGERLKTL
STCLSHMSTVLLFYVPFMGAASMIHRFWEHLSPVVHMVMADIYLLLPPVLNPIVYSVKTK
QI

>Hs_OR10H2 (ENSP00000306095)
MLGLNHTSMSEFILVGFSAFPHLQLMLFLLFLLMYLFTLLGNLLIMATVWSERSLHTPMY
LFLCVLSVSEILYTVAIIPRMLADLLSTQRSIAFLACASQMFFSFSFGFTHSFLLTVMGY
DRYVAICHPLRYNVLMSPRGCACLVGCSWAGGSVMGMVVTSAIFQLTFCGSHEIQHFLCH
VPPLLKLACGNNVPAVALGVGLVCIMALLGCFLLILLSYAFIVADILKIPSAEGRNKAFS
TCASHLIVVIVHYGFASVIYLKPKGPHSQEGDTLMATTYAVLTPFLSPIIFSLRNKELKV
AMKRTFLSTLYSSGT

>Hs_OR52A5 (ENSP00000303469)
MPTFNGSVFMPSAFILIGIPGLESVQCWIGIPFSAMYLIGVIGNSLILVIIKYENSLHIP
MYIFLAMLAATDIALNTCILPKMLGIFWFHLPEISFDACLFQMWLIHSFQAIESGILLAM
ALDRYVAICIPLRHATIFSQQFLTHIGLGVTLRAAILIIPSLGLIKCCLKHYRTTVISHS
YCEHMAIVKLATEDIRVNKIYGLFVAFAILGFDIIFITLSYVQIFITVFQLPQKEARFKA
FNTCIAHICVFLQFYLLAFFSFFTHRFGSHIPPYIHILLSNLYLLVPPFLNPIVYGVKTK
QIRDHIVKVFFFKKVT

>Hs_OR52M1 (ENSP00000353343)
MLTFHNVCSVPSSFWLTGIPGLESLHVWLSIPFGSMYLVAVVGNVTILAVVKIERSLHQP
MYFFLCMLAAIDLVLSTSTIPKLLGIFWFGACDIGLDACLGQMFLIHCFATVESGIFLAM
AFDRYVAICNPLRHSMVLTYTVVGRLGLVSLLRGVLYIGPLPLMIRLRLPLYKTHVISHS
YCEHMAVVALTCGDSRVNNVYGLSIGFLVLILDSVAIAASYVMIFRAVMGLATPEARLKT
LGTCASHLCAILIFYVPIAVSSLIHRFGQCVPPPVHTLLANFYLLIPPILNPIVYAVRTK
QIRESLLQIPRIEMKIR

>Hs_OR2Y1 (ENSP00000312403)
MGSFNTSFEDGFILVGFSDWPQLEPILFVFIFIFYSLTLFGNTIIIALSWLDLRLHTPMY
FFLSHLSLLDLCFTTSTVPQLLINLCGVDRTITRGGCVAQLFIYLALGSTECVLLVVMAF
DRYAAVCRPLHYMAIMHPHLCQTLAIASWGAGFVNSLIQTGLAMAMPLCGHRLNHFFCEM
PVFLKLACADTEGTEAKMFVARVIVVAVPAALILGSYVHIAHAVLRVKSTAGRRKAFGTC
GSHLLVVFLFYGSAIYTYLQSIHNYSEREGKFVALFYTIITPILNPLIYTLRNKDVKGAL
WKVLWRGRDSG

>Hs_OR51M1 (ENSP00000333196)
MSVQYSLSPQFMLLSNITQFSPIFYLTSFPGLEGIKHWIFIPFFFMYMVAISGNCFILII
IKTNPRLHTPMYYLLSLLALTDLGLCVSTLPTTMGIFWFNSHSIYFGACQIQMFCIHSFS
FMESSVLLMMSFDRLVAICHPLRYSVIITGQQVVRAGLIVIFRGPVATIPIVLLLKAFPY
CGSVVLSHSFCLHQEVIQLACTDITFNNLYGLMVVVFTVMLDLVLIALSYGLILHTVAGL
ASQEEQRRAFQTCTAPLCAVLVFFVPMMGLSLVHRFGKHAPPAIHLLMANVYLFVPPMLN
PIIYSIKTKEIHRAIIKFLGLKKASK

>Hs_OR1I1 (ENSP00000209540)
MEPEKQTEISEFFLQGLSEKPEHQTLLFTMFLSTYLVTIIGNALIILAIITDSHLHTPMY
FFLFNLSLVDTLLSSTTVPKMLANIQAQSRAIPFVGCLTQMYAFHLFGTMDSFLLAVMAI
DRFVAIVHPQRYLVLMCSPVCGLLLGASWMITNLQSLIHTCLMAQLTFCAGSEISHFFCD
LMPLLKLSGSDTHTNELVIFAFGIVVGTSPFSCILLSYIRIFWTVFKIPSTRGKWKAFST
CGLHLTVVSLSYGTIFAVYLQPTSPSSSQKDKAAALMCGVFIPMLNPFIYSIRNKDMKAA
LGKLIGKVAVPCPRPEQLLDVYHVPGSLLAARDTEMHPIPYPGGVQSLAGNRDME

>Hs_OR52L1 (ENSP00000330338)
MTLVSFFSFLSKPLIMLLSNSSWRLSQPSFLLVGIPGLEESQHWIALPLGILYLLALVGN
VTILFIIWMDPSLHQSMYLFLSMLAAIDLVLASSTAPKALAVLLVHAHEIGYIVCLIQMF
FIHAFSSMESGVLVAMALDCYVAICHPLHHSTILHPGVIGCIGMVVLVRGLLLLIPFPIL
LGKLIFCQATIIGHAYCEHMAVVKLACSETTVNRAYGLTMALLVIGLDVLAIGVSYAHIL
QAVLKVPGSEARLKAFSTCGSHICVILVFYVPGIFSFLTHRFGHHVPHHVHVLLATWYLL
MPPALNPLVYGVKTQQIRQRVLRVFTQKD

>Hs_OR56B4 (ENSP00000321196)
MDTSTSVTYDSSLQISQFILMGLPGIHEWQHWLSLPLTLLYLLALGANLLIIITIQHETV
LHEPMYHLLGILAVVDIGLATTIMPKILAIFWFDAKAISLPMCFAQIYAIHCFFCIESGI
FLCMAVDRYIAICRPLQYPSIVTKAFVFKATGFIMLRNGLLTIPVPILAAQRHYCSRNEI
EHCLCSNLGVISLACDDITVNKFYQLMLAWVLVGSDMALVFSSYAVILHSVLRLNSAEAM
SKALSTCSSHLILILFHTGIIVLSVTHLAEKKIPLIPVFLNVLHNVIPPALNPLACALRM
HKLRLGFQRLLGLGQDVSK

>Hs_OR51D1 (ENSP00000350222)
MQKPQLLVPIIATSNGNLVHAAYFLLVGIPGLGPTIHFWLAFPLCFMYALATLGNLTIVL
IIRVERRLHEPMYLFLAMLSTIDLVLSSITMPKMASLFLMGIQEIEFNICLAQMFLIHAL
SAVESAVLLAMAFDRFVAICHPLRHASVLTGCTVAKIGLSALTRGFVFFFPLPFILKWLS
YCQTHTVTHSFCLHQDIMKLSCTDTRVNVVYGLFIILSVMGVDSLFIGFSYILILWAVLE
LSSRRAALKAFNTCISHLCAVLVFYVPLIGLSVVHRLGGPTSLLHVVMANTYLLLPPVVN
PLVYGAKTKEICSRVLCMFSQGGK

```
>Xt_or51g2 (ENSXETP00000032492)
QADVTMAPPTNMSWTFTLSGIPELGGNQQWFAPPLCALYVTAFLGNCTILFLIRTERGLHQPMFLLLA
MLAVTDVGVSLTTLPTMLGIFCFNHHEIYSELCLTQMYFLHTFAAMESGVLVAMAVDRLVAICAPLRY
ASVLTNSAVGRMGLVIVARGVCVVLPVPLLTRRFPFCKTRVLSHSYCLHQDVIRLACADTTVNSVYGL
VAVLLTKGVDSMFILISYGLILRAVINMRANDARLKAFSTCVAHMCAVLLFYIPLIGLSVLHRVGTHA
SPLLAIVMADVYLLLPPVVNPVIYSVNTKQIRHKMTRLFRRRRVGVPA
```

***N. vectensis* sequence that had the highest alignment score against the 7tm_GPCR_srw domain.**

```
>Nv_205247 (jgi|Nemve1|205247|fgenesh1_pg.scaffold_57000099)
MAVNSSNVPLCWGVDFSSLAANKSFLIFICVLNNSAAMSSVFGNLTVIVSIWRTPSIHSPSFVLISIR
CVLDMSIGLFMQGLCSPVLLLLYVDNLQGFCSAAGPWLQMGLFINGAILFMVMLTAMDIFSAIKLKLR
YRQLVTTRRMIIAVVLMVLLSAALSALSVTLPLVEHFLVMTVVVGIALNMTFFFYGLSYNELRRHQRT
VQPCQQTTTSIAKYRKTFTTLVLILGFLLLCYLPADLTFLVVAIYGFTEASIQAVADVFSLQDWETRC
IRMFTTLGP
```

***T. adhaerens* sequence that had two significant Pfam HMM profile hits corresponding to Srsx (7tm_GPCR_Srsx) domain and 7tm_1 domain**

```
>Ta_58780 (jgi|Triad1|58780|fgeneshTA2_pg.C_scaffold_8000328)
MNASKLASCIGNNCRNFSNATDNIRQRPANPIQLLLTIAGCMGIIPNIILVIGFYTKKSFKKPTYCLL
ANLAVSDMILSLGSLSNVIVNSLEASASIQTLIMISNERYLAIFRPTSELTRKKANFLCLIAWIISLS
ISFPFIITTSVTNVIPKQCVAFEKFTNWTAIIFVVIFIFQFALPTVVIITLYSLILHRLRKTVQGQTD
SSKSKKLKRRTIYMLLTTTIIFLAFSTPWAISLAIMAITRSLPAKIVVDPNYPIAGGIVRISRFVVPF
TAIYNPIIYCIFNKHIRQLYFSCCFNFIKNSAVVPSVYTNSDNGQIDTVNSTAAANSNTIIVSNTAAG
IVANSTTTEFADLNTTATADLNSTVVQKYISKDNEAMSTD
```
